# Identifications of immune-responsive genes for adaptative traits by comparative transcriptome analysis of spleen tissue from Kazakh and Suffolk sheep

**DOI:** 10.1038/s41598-021-82878-x

**Published:** 2021-02-04

**Authors:** Hua Yang, Yong-Lin Yang, Guo-Qing Li, Qian Yu, Jinzeng Yang

**Affiliations:** 1 State Key Laboratory of Sheep Genetic Improvement and Healthy Production, Shihezi, 832000 China; 2 Institute of Animal Husbandry and Veterinary Medicine, Xinjiang Academy of Agricultural and Reclamation Science, Shihezi, 832000 China; 3 Department of Human Nutrition, Food and Animal Sciences, University of Hawaii, Honolulu, HI 96822 USA

**Keywords:** Animal breeding, Gene expression profiling

## Abstract

Aridity and heat are significant environmental stressors that affect sheep adaptation and adaptability, thus influencing immunity, growth, reproduction, production performance, and profitability. The aim of this study was to profile mRNA expression levels in the spleen of indigenous Kazakh sheep breed for comparative analysis with the exotic Suffolk breed. Spleen histomorphology was observed in indigenous Kazakh sheep and exotic Suffolk sheep raised in Xinjiang China. Transcriptome sequencing of spleen tissue from the two breeds were performed via Illumina high-throughput sequencing technology and validated by RT-qPCR. Blood cytokine and IgG levels differed between the two breeds and IgG and IL-1β were significantly higher in Kazakh sheep than in Suffolk sheep (*p* < 0.05), though spleen tissue morphology was the same. A total of 52.04 Gb clean reads were obtained and the clean reads were assembled into 67,271 unigenes using bioinformatics analysis. Profiling analysis of differential gene expression showed that 1158 differentially expressed genes were found when comparing Suffolk with Kazakh sheep, including 246 up-regulated genes and 912 down-regulated genes. Utilizing gene ontology annotation and pathway analysis, 21 immune- responsive genes were identified as spleen-specific genes associated with adaptive traits and were significantly enriched in hematopoietic cell lineage, natural killer cell-mediated cytotoxicity, complement and coagulation cascades, and in the intestinal immune network for IgA production. Four pathways and up-regulated genes associated with immune responses in indigenous sheep played indispensable and promoting roles in arid and hot environments. Overall, this study provides valuable transcriptome data on the immunological mechanisms related to adaptive traits in indigenous and exotic sheep and offers a foundation for research into adaptive evolution.

## Introduction

Sheep are one of the most important livestock species in the world, providing meat, milk and wool^1^. Sheep were domesticated in the Fertile Crescent and subsequently dispersed across the globe around 9500–10,000 years ago, eventually becoming critical to the household income of the world’s poorest people^1^. Sheep have a range of unique adaptive traits, which evolved through long-term natural and artificial selection, that enable them to survive and reproduce in extreme environments. However, many stressors impact sheep productivity and welfare, including parasites, seasonally poor nutrition, as well as environmental conditions such as aridity, humidity, heat, and cold. Environmental stressors have some of the most harmful impacts on natural immunity, survival, growth, production performance, and fertility^2^. Aridity and extreme temperatures compromise immune function, resulting in poor reproduction and production performance^3^. In order to adapt to extremely harsh environments, a series of immune responses are regulated by immune factors in the body^4^.

Previous research on adaptation and innate immunity of sheep has focused on genes including *HSP-70*^5^, *ENOX2*^6^, *MHC*^7^, *TLR*^8^ and *NRAMP1*^9^. For example, HSP-70 mediates cell viability of blood mononuclear cells and helps Pelibuey sheep regulate body temperature more efficiently under conditions of environmental hyperthermia^5^. High-level expression of the *ENOX2* gene has been found to improve long-term heat stress resistance in goats^6^. MHC is a central molecule for antigen presentation, which codes for specialized antigen presenting glycoproteins and has been used as a candidate gene for research into disease resistance in farm animals^7,10^. In recent years, high-throughput SNP technologies, whole-genome sequencing and omics have been performed on a wide range of organisms that live in harsh or extreme environments. Using 600 K high density SNP genotypes, *HERC2* and *CYFIP1* genes associated with regulating innate and adaptive immunity have been identified in five sheep breeds from different geographical locations that have extremely dry or humid conditions^11^. Whole genome sequences have been used to identify HBB and RXFP2 genes as adaptive introgression from Argali in Tibetan sheep in high-altitude^12^. A variety of novel genes, pathways, and GO categories associated with hypoxia responses at high altitudes and water reabsorption in arid environments were detected in native sheep^13^. RNA-seq has recently been used to investigate the differentially expressed genes (DEGs) and novel transcript units in many types of organisms including some major organs of sheep, such as muscles^14^, bone^15^, skin^16^, ovaries^17^, and uterine epithelia^18^. However, transcriptome information associated with adaptive immunity traits in the sheep spleen is limited, although a number of immune-related genes and pathways have been identified in beef cattle selected divergently selected for body weight gain and feed intake, and Chinese native cattle breed-Yunnan Humped cattle in comparison with Holstein and Yunnan^4,19^.

**Figure 1:**
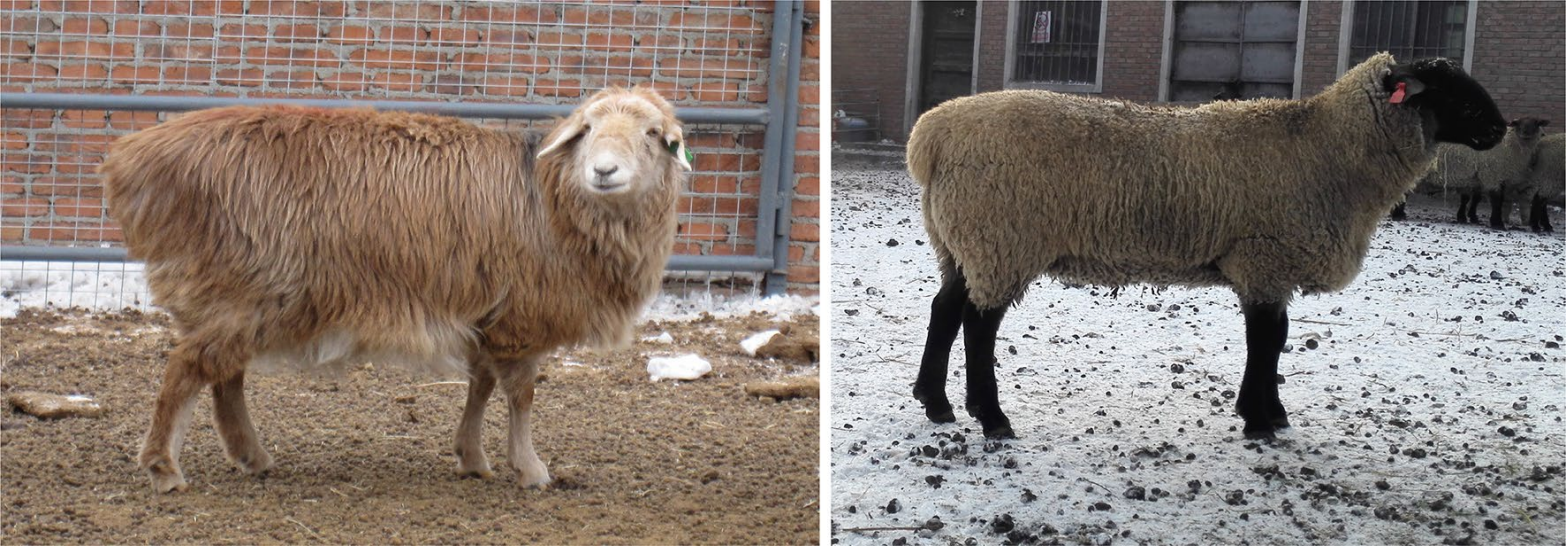
Kazakh (left) and Suffolk (right) sheep at 7 months of age, photographed by Hua Yang.

Kazakh sheep (Fig. 1) are indigenous sheep that have a range of unique adaptive traits, which enable them to survive and be productive in dry, hot, and cold environments on the edge of the desert in northwestern Xinjiang China. They are very hardy to the environment and diseases as the sheep are bred to conform to nomadic life in the semi-deserts and deserts of Kazakhstan and Xinjiang China. They are well adapted to severe winter frosts and droughts in summer. Their body frame and grazing ability are strong, they can travel over long distances and thrive on poor feed conditions. Kazakh sheep are mostly bred for their meat and tail fat. At four months of age, carcass weight is around 20 kg with tail fat weight is around 3–4 kg. Suffolk is a British breed of domestic sheep, resulted from historical crossbreeding between Norfolk Horn ewes and Southdown rams. It is a polled, black-faced breed, and is raised primarily for its meat. It has been exported to many countries^20^. Suffolk sheep were imported to the temperate continental climate of Xinjiang China from the Mediterranean climate of Western Australia for a breeding program to improve the genetics of mutton production. The aim of this study was to investigate immune differences between the indigenous Kazakh and exotic Suffolk sheep breeds by transcriptome sequencing. The spleen transcriptomes were characterized and the immune-response genes associated with adaptive traits were identified in order to ascertain the genetic differences responsible for variations in the levels of resistance and adaptive traits between the two breeds of sheep.

## Results

### Serum cytokines analysis

Higher levels of seven cytokines, including interferon (IFN)-α, IFN-γ, interleukin (IL)-2, IL-5, IL-6, IL-8, and IL-12, were found in the serum of Kazakh sheep than in Suffolk sheep, thought the differences were not statistically significant. However, IgG and IL-1β were significantly higher in Kazakh sheep than in Suffolk sheep (*p* < 0.05). There were no differences in any of the nine immune parameters tested between rams and ewes (*p* > 0.05) in either sheep species (Table 1).

**Table 1:** Contents of IgG and 8 Cytokines in serum of Suffolk and Kazakh sheep*.

Cytokines	Serum content			
	Kazakh (n = 94)	Suffolk (n = 56)	Rams (n = 65)	Ewes (n = 94)
IgG (mg/ml)	3.43 ± 0.33^a^	2.63 ± 0.19^b^	2.91 ± 0.28	2.99 ± 0.23
IFN-α (pg/ml)	387.04 ± 38.15	318.11 ± 23.76	330.50 ± 33.07	357.20 ± 27.47
IFN-γ (pg/ml)	337.52 ± 36.39	310.40 ± 20.71	316.30 ± 28.23	325.07 ± 26.14
IL-1β (pg/ml)	270.32 ± 22.27^a^	217.57 ± 9.29^b^	214.35 ± 14.98	256.27 ± 14.84
IL-2 (pg/ml)	481.83 ± 38.08	447.42 ± 26.98	477.81 ± 34.68	450.20 ± 29.11
IL-5 (pg/ml)	458.32 ± 34.95	383.89 ± 27.76	382.95 ± 31.26	436.01 ± 29.98
IL-6 (pg/ml)	944.71 ± 86.25	881.00 ± 54.06	964.86 ± 77.48	867.06 ± 59.85
IL-8 (pg/ml)	310.67 ± 27.21	255.14 ± 14.45	252.96 ± 18.62	295.05 ± 20.05
IL-12 (pg/ml)	379.75 ± 40.16	300.90 ± 24.66	334.06 ± 33.73	332.50 ± 29.36

*Means ± SEM marked with different lower-case letter either a or b in the same line were significant at *P* < 0.05.

### Histomorphology observation of spleen

**Figure 2:**
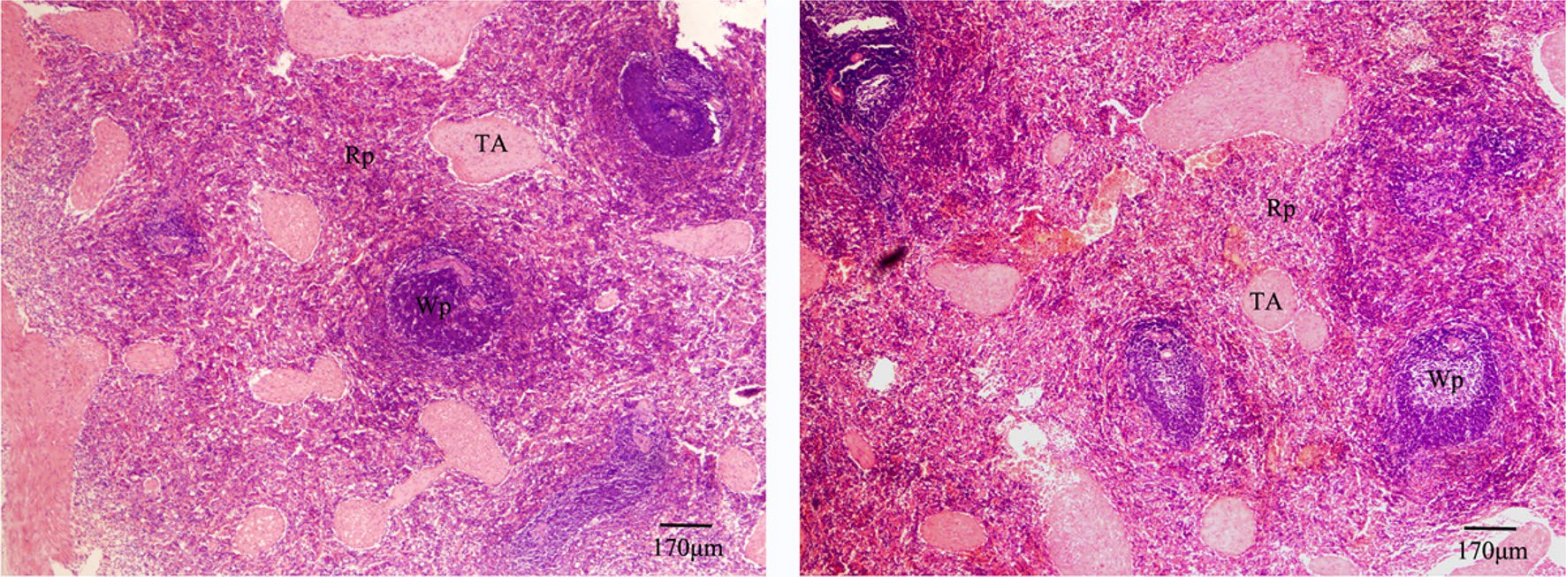
Representative HE-stained histology images of spleen tissue sections of Kazakh (left) and Suffolk (right) sheep under 40X magnification. The trabeculae splenicae (TA), white pulps (Wp), and red pulps (Rp) are clear and normal with no difference between these two breeds.

HE-stained histology images of spleen tissue sections from Kazakh (left) and Suffolk sheep (right) (Fig. 2). The trabeculae splenicae, white pulp and red pulp were clear and normal in sheep from both species. There were no apparent differences between the two breeds, when under the same microscopic scale and amplification.

### Overview of transcriptome sequencing and functional annotation

A total of 52,043,368 clean reads and 4,683,903,120 nucleotides (nt) were obtained from the libraries. The final assembly length of 60,843,689 nt and 242,485 contigs were obtained via SOAPdenovo2. A total of 93,608 unigenes with a final assembly length of 46,448,371 nt were obtained with an average contig length of 496 nt and N50 equal to 594 nt. A total of 67,271 unigenes were annotated by aligning with the NR database, NT database, Swiss-Prot database, KEGG database, COG database and GO database. Output statistics, assembly quality statistics and length distribution of the unigenes indicated substantial transcriptome sequencing quality of the sheep spleen.

### Analysis of gene expression profiling in spleen

A total of 417,696 raw data of distinct tags and 5,995,238 raw data of total tags were acquired for digital gene expression profiling (DGE) libraries of Kazakh sheep, and 353,896 raw data of distinct tags and 5,756,578 raw data of total tags were acquired for DGE libraries of Suffolk sheep. The total clean tags were 95.54% and 96.01% (> 95%) in Kazakh sheep and Suffolk sheep, respectively. All of the clean tags were aligned onto the transcriptome data and genes (93,608 unigenes) of reference sheep spleen. Unambiguous tag mapping was used to map 43.55% of distinct tags, 70.65% of total tags, and 28.31% of genes in Kazakh sheep and 46.55% of distinct tags, 66.86% of total tags, and 25.79% of genes in Suffolk sheep to reference genes. In addition, 32.51% of distinct tags in Kazakh sheep and 28.60% of distinct tags in Suffolk sheep were mapped to the sheep genome assembly (OAR3.1) (Table 2). Saturation analysis results for sequencing in Kazakh and Suffolk sheep showed that when the sequencing counts reached 2 M or higher, the number of detected genes nearly ceased to increase, validating the integrity of the library for use in further analysis.

**Table 2:** Summary of the sequencing tags alignment to the reference genes and genome.

	Kazakh sheep			Suffolk sheep		
	Distinct tag	Total tag number	Number of gene	Distinct tag	Total tag number	Number of gene
Raw data	417,696	5,995,238		353,896	5,756,578	
Clean tag	157,130	5,727,944	93,608	131,821	5,527,151	93,608
All tag mapping to sense gene	43,275 (27.54%)	2,783,854 (48.60%)	26,210 (28.00%)	38,598 (29.28%)	2,960,765 (53.57%)	24,271 (25.93%)
Unambiguous tag mapping to sense gene	35,724 (22.74%)	2,305,489 (40.25%)	19,724 (21.07%)	31,948 (24.24%)	2,109,766 (38.17%)	17,997 (19.23%)
All tag mapping to anti-Sense gene	38,274 (24.36%)	2,055,280 (35.88%)	23,957 (25.59%)	34,501 (26.17%)	1,885,756 (34.12%)	22,107 (23.62%)
Unambiguous tag mapping to anti-Sense gene	32,709 (20.82%)	1,741,196 (30.40%)	18,593 (19.86%)	29,415 (22.31%)	1,585,436 (28.68%)	16,910 (18.06%)
All tag mapping to gene	81,549 (51.90%)	4,839,134 (84.48%)	33,608 (35.90%)	73,099 (55.45%)	4,846,521 (87.69%)	31,084 (33.21%)
Unambiguous tag mapping to gene	68,433 (43.55%)	4,046,685 (70.65%)	26,497 (28.31%)	61,363 (46.55%)	3,695,202 (66.86%)	24,142 (25.79%)
All clean tag mapping to genome	51,086 (32.51%)	569,332		37,701 (28.60%)	406,040	

### Identification of DEGs in spleen

**Figure 3:**
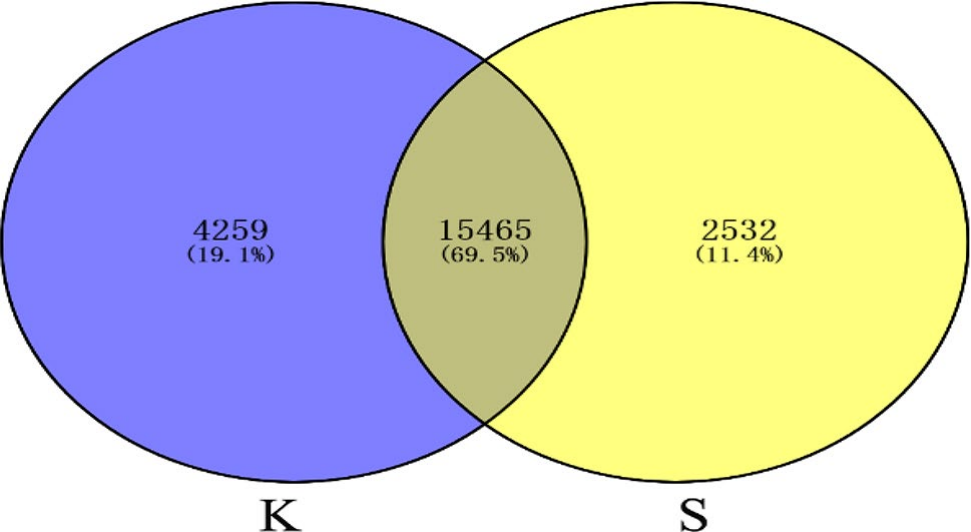
The number of expressed genes between two libraries. Venn diagram showing the number of reference genes specifically expressed in one library and those expressed in Kazakh sheep library (K) and Suffolk sheep library (S).

**Figure 4:**
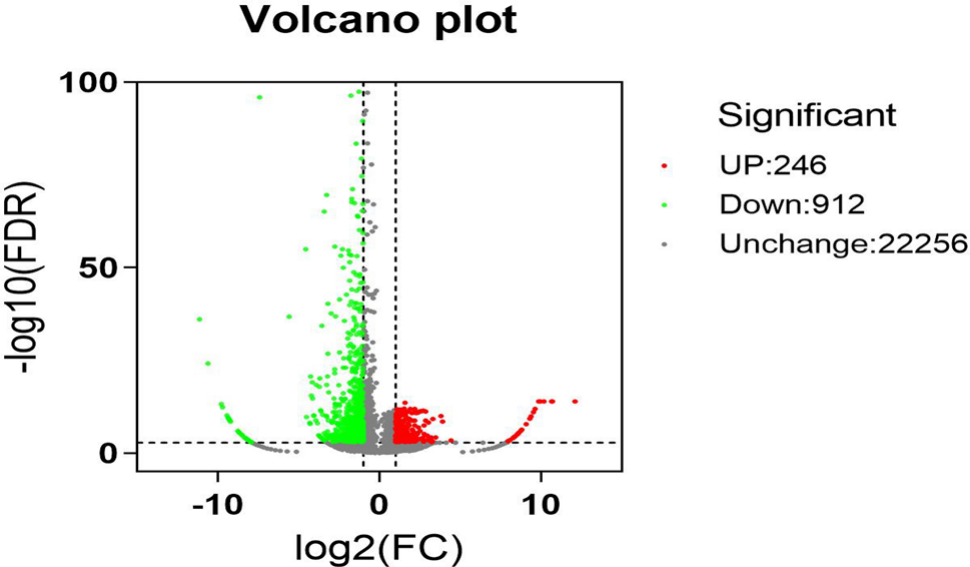
Volcano plot of differentially expressed gene distribution trends between indigenous Kazakh sheep and exotic Suffolk sheep. Each dot represents a gene. The up-regulated genes are marked in red and down-regulated genes are marked in green. Similar gene expression levels are marked in gray.

A Venn diagram was constructed showing expressed genes in the two libraries. The TPM method was used to identify expression of 19,724 and 17,997 reference genes in the Kazakh and Suffolk sheep libraries, respectively, and 15,465 shared genes (Fig. 3). A total of 1,158 significantly DEGs were identified between the two libraries, of which 246 genes were up-regulated and 912 genes were down-regulated in Suffolk sheep (Fig. 4). The significant DEGs between two libraries were listed in Supplementary Table S1.

### Functional enrichment of DEGs

**Figure 5:**
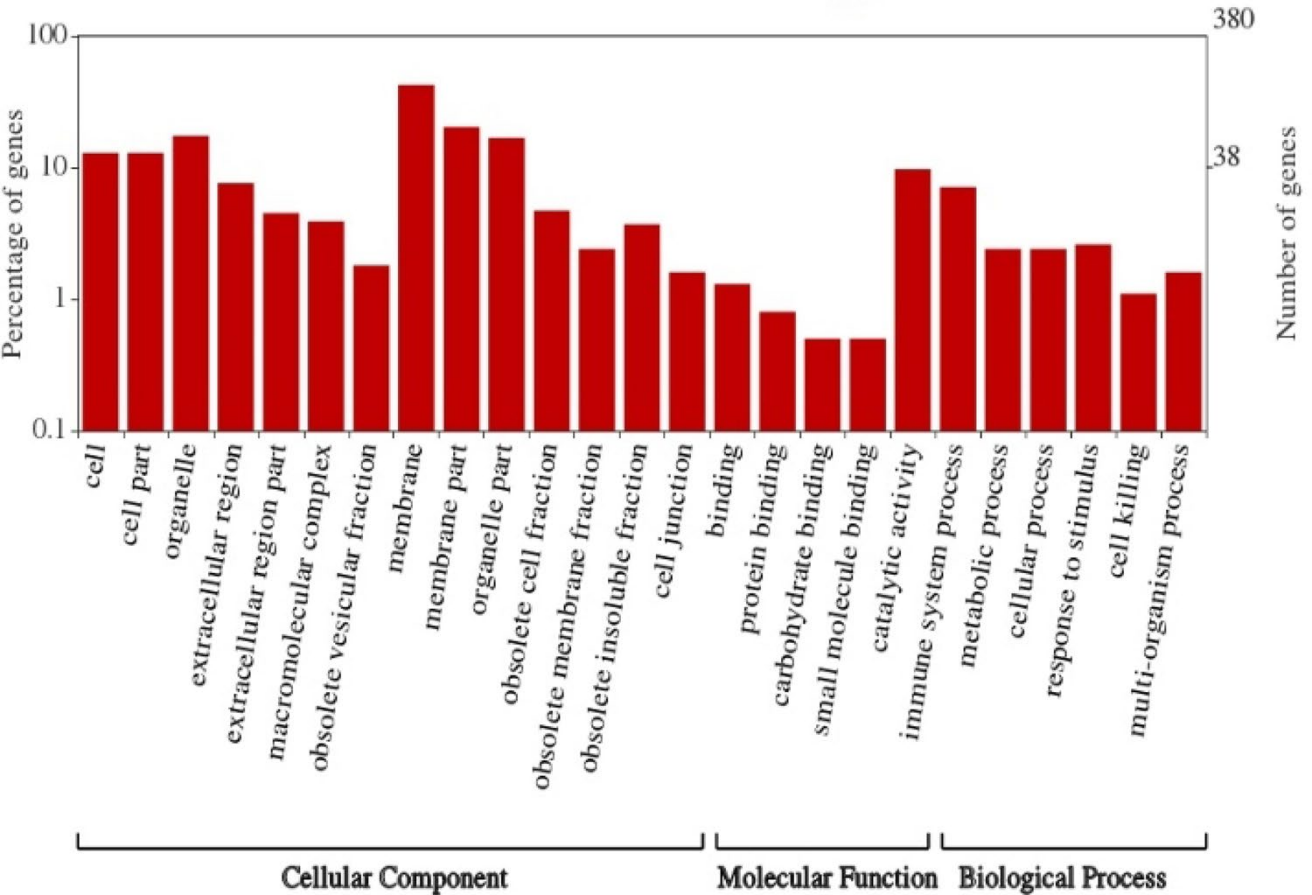
Percentages of DEGs identified in cellular components, molecular functions and biological processes. The left axis shows the percentage of genes in a category and the right axis shows the number of genes.

To gain insights into biological implications, the enrichment of DEGs in terms of GO were tested in this study (Fig. 5). The summary of all significantly enriched GO assignments of DEGs are shown in the results. In the cellular component category, a significant percentage of clusters were assigned to membrane (42.4%), membrane part (20.3%), organelle (17.4%), and organelle part (16.8%). The main functional groups of DEGs in molecular function category were assigned to catalytic activity (9.7%) and binding (1.3%). In the biological process category, the terms were related to immune system process (7.1%), metabolic process (2.4%), cellular process (2.4%), response to stimulus (2.6%), multi-organism process (1.6%), and cell killing (1.1%).

**Figure 6:**
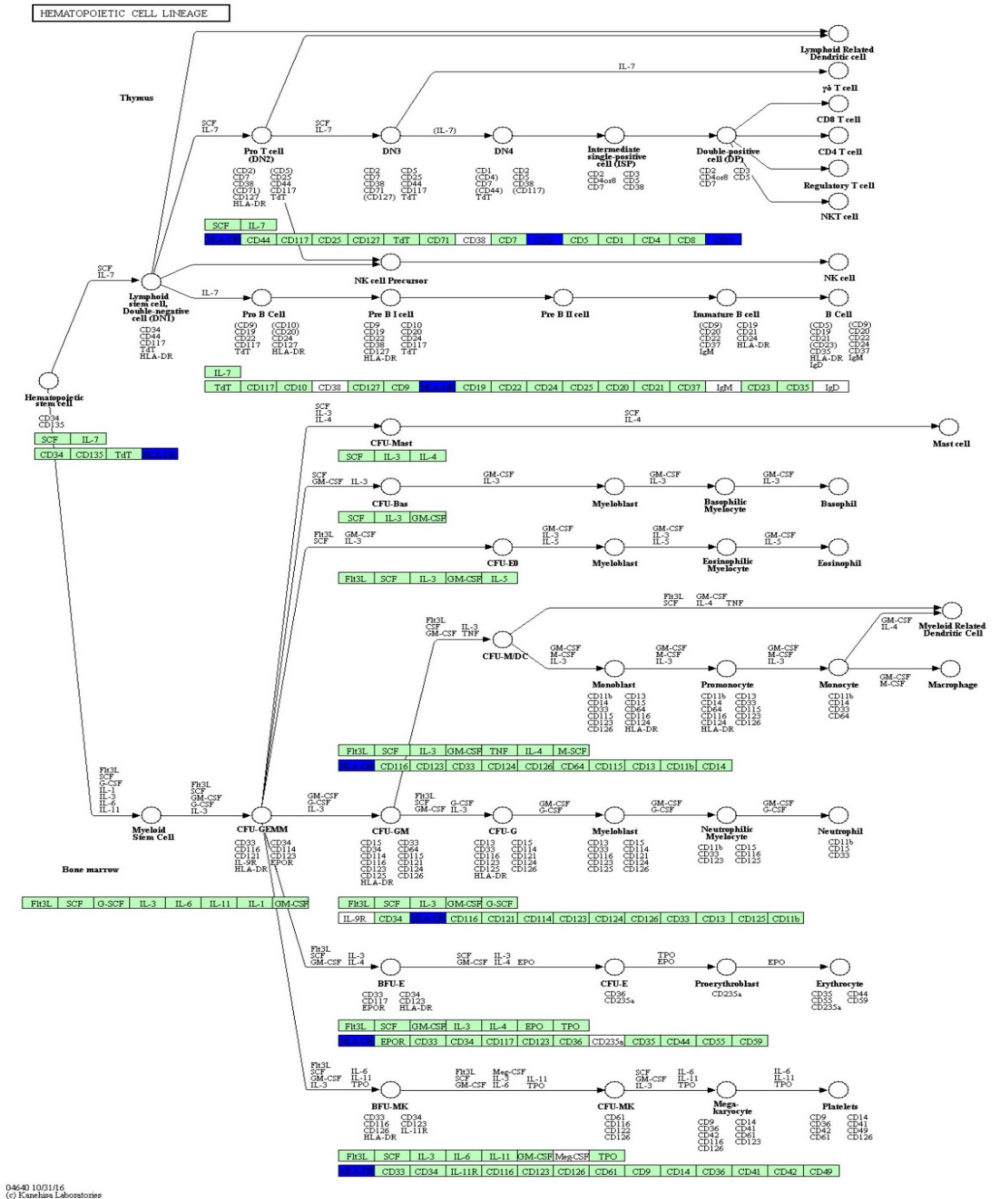
Differentially expressed genes involved in hematopoietic cell lineage in the spleens of two sheep breeds. The blue-labelled genes were significantly down-regulated (*p* < 0.05). The green-labelled genes were expressed in both sheep spleens. KEGG pathway annotation was conducted using the DAVID website (http://david.abcc.ncifcrf.gov/) and searching the KEGG database.

**Figure 7:**
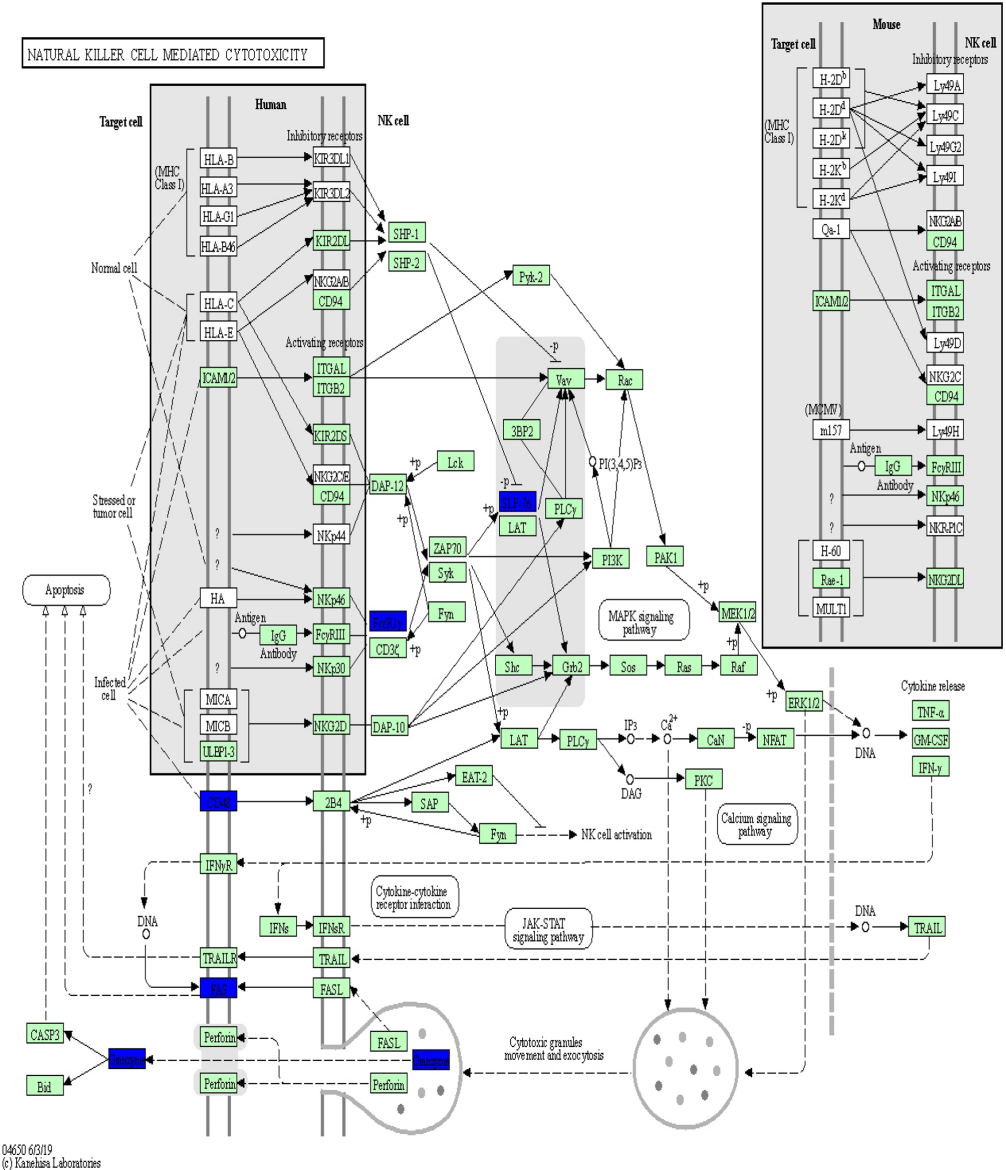
Differentially expressed genes involved in natural killer cell mediated cytotoxicity in the spleen of two sheep breeds. The blue-labelled genes were significantly down-regulated (*p* < 0.05). The green-labelled genes were expressed in both sheep spleens. KEGG pathway annotation was conducted using the DAVID website (http://david.abcc.ncifcrf.gov/) and searching the KEGG database.

**Figure 8:**
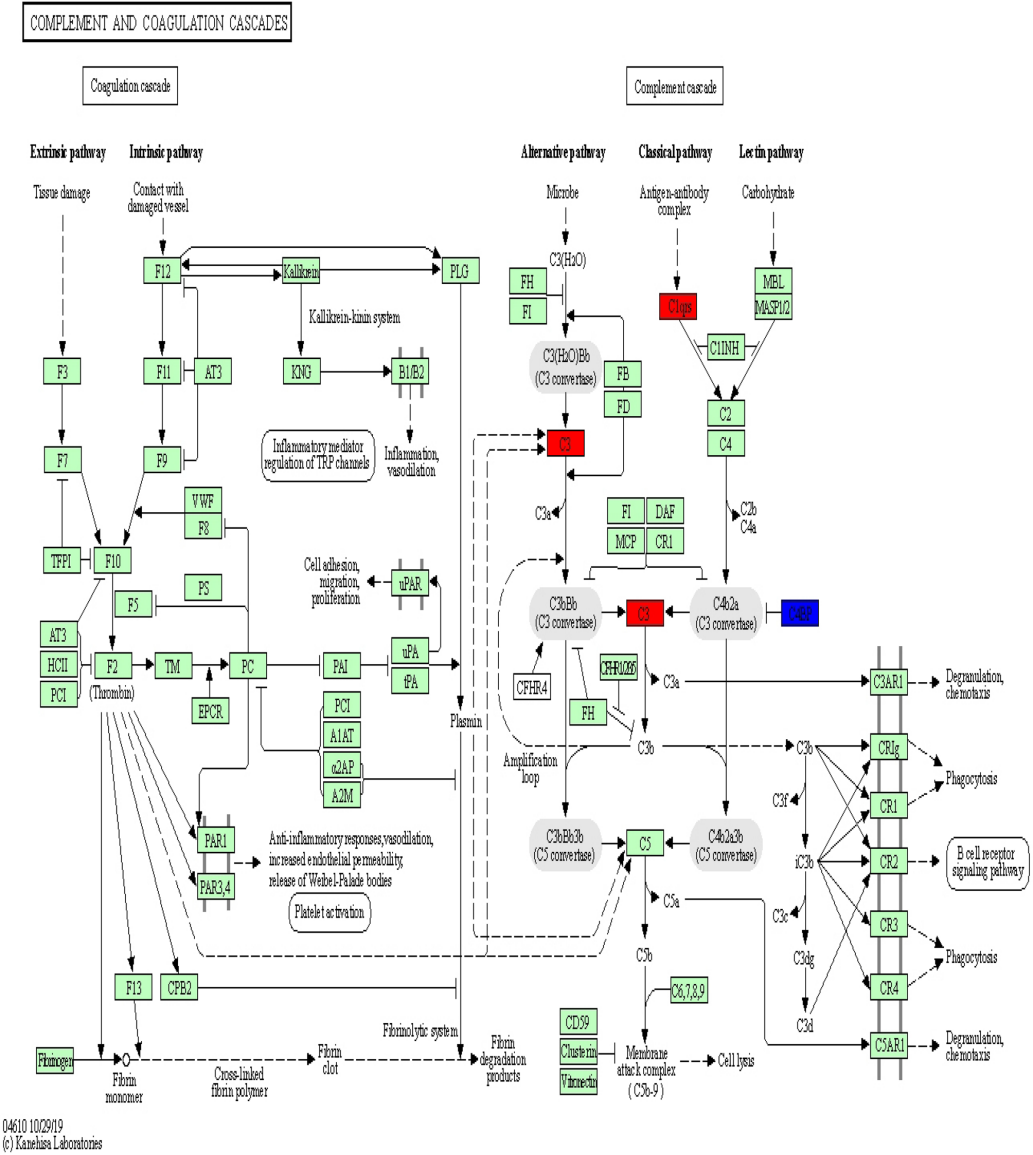
Differentially expressed genes involved in complement and coagulation cascades in the spleen of two sheep breeds. The red-labelled genes were significantly up-regulated (*p* < 0.05). The blue-labelled genes were significantly down-regulated (*p* < 0.05). The green-labelled genes were expressed in both sheep spleens. KEGG pathway annotation was conducted using the DAVID website (http://david.abcc.ncifcrf.gov/) and searching the KEGG database.

**Figure 9:**
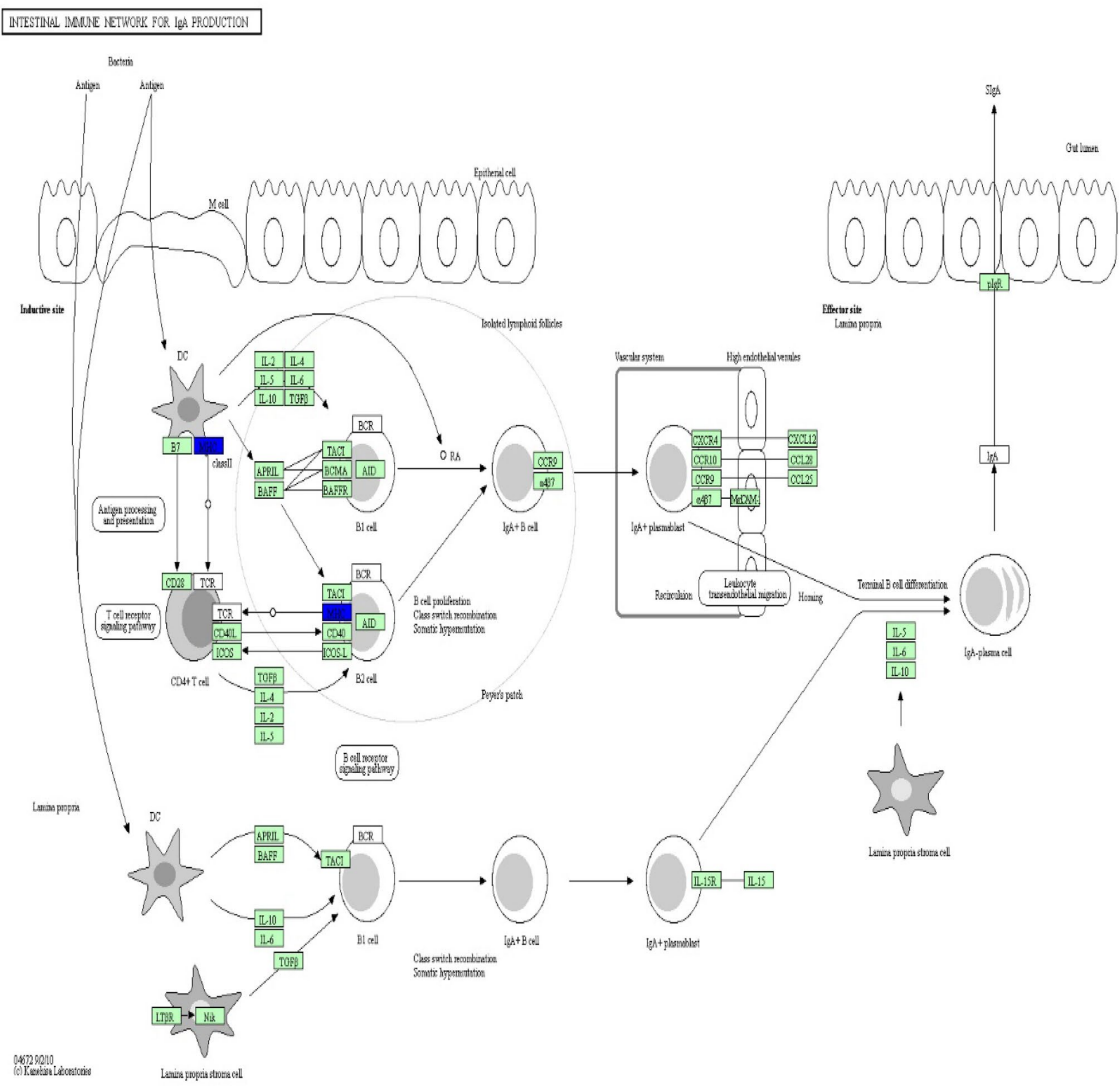
Differentially expressed genes involved in the intestinal immune network for IgA production in the spleen of two sheep breeds. The blue-labelled genes were significantly down-regulated (*p* < 0.05). The green-labelled genes were expressed in both sheep spleens. KEGG pathway annotation was conducted using the DAVID website (http://david.abcc.ncifcrf.gov/) and searching the KEGG database.

KEGG pathway analysis was conducted to categorize and annotate 384 DEGs. Further comparisons in the KEGG pathway database identified four significantly enriched pathways that were associated with the immune system (Table 3), including hematopoietic cell lineage, natural killer cell mediated cytotoxicity, complement and coagulation cascades and intestinal immune network for IgA production. The most significant difference was in the hematopoietic cell lineage. Ten hematopoietic cell lineage genes (*IgD*, *DQA2*, *DQB*, *DOB*, *CD3D*, *MS4A1*, *CSF1R*, *CD2*, *ITGA2B*, and *CD3E*) were significantly enriched by comparing Suffolk with Kazakh sheep (*P* value = 0.0004). The genes *DQA2* (HLA-DR), *DQB* (HLA-DR), *CD3D* (CD3), *CD3E* (CD3), and *CD2* were significantly down-regulated (Fig. 6). Eight natural killer cell-mediated cytotoxicity genes (*IgD*, *GRB10*, *GZMB*, *FAS*, *FCER1G*, *TYROBP*, *LCP2*, and *CD48*) were significantly enriched by comparing Suffolk with Kazakh sheep (*P* value = 0.0075). The genes *GZMB* (Granzyme), *FAS*, *FCER1G* (FcεR1y), *LCP2* (SLP-76), and *CD48* were significantly down-regulated (Fig. 7). Four complement and coagulation cascade genes (*C1s*, *HP*, *C3*, and *C4BPA*) were significantly enriched by comparing Suffolk with Kazakh (*P* value = 0.0112). The genes *C1s* (C1qrs) and *C3* were significantly up-regulated, and gene *C4BPA* (C4BP) was significantly down-regulated (Fig. 8). Four intestinal immune networks for IgA production genes (*IgD*, *DQA2*, *DQB*, and *DOB*) were significantly enriched by comparing Suffolk with Kazakh sheep (*P* value = 0.0158). The genes *DQA2* (MHC) and *DQB* (MHC) were significantly down-regulated (Fig. 9).

**Table 3:** Significantly enriched pathways of DEGs associated with immune system.

Pathway ID	Pathway	Genes	*P* value
ko04640	Hematopoietic cell lineage	IgD, DQA2, DQB, DOB, CD3D, MS4A1, CSF1R, CD2, ITGA2B, CD3E	3.77E−04
ko04650	Natural killer cell mediated cytotoxicity	IgD, GRB10, GZMB, FAS, FCER1G, TYROBP, LCP2, CD48	7.52E−03
ko04610	Complement and coagulation cascades	C1s, HP, C3, C4BPA	1.12E−02
ko04672	Intestinal immune network for IgA production	IgD, DQA2, DQB, DOB	1.58E − 02

The results from Table 4 show that a total of 21 immune-responsive genes were identified in the spleen according to their KEGG assigned pathways including five up-regulated genes and 16 down-regulated genes in the Suffolk sheep spleen. Furthermore, two up-regulated genes (*DQA2* and *C1s*) and three down-regulated genes (*DQB*, *DOB* and *GZMB*) showed the most significant differential expression with an absolute value of log2 ratio set at least twofold higher. *DQA2* and *C1s* had the highest expressions in the spleen tissue of Suffolk sheep, while *DQB*, *DOB* and *GZMB* had the highest expressions in the spleen tissue of Kazakh sheep.

**Table 4:** List of differentially expressed genes associated with immune system.

Gene	Gene name	log_2_ Ratio(S/H)	*P* value	FDR	Expression
DQA2	MHC class II, DQ alpha 2	2.86	1.50E−13	6.30E−12	Up-regulated
C1s	Complement C1s	2.75	1.81E−05	2.48E−04	Up-regulated
HP	Haptoglobin	1.44	2.22E−10	6.45E−09	Up-regulated
GRB10	Growth factor receptor-bound protein 10	1.37	3.70E−05	4.74E−04	Up-regulated
C3	Complement C3	1.37	2.04E−3	8.35E−2	Up-regulated
DQB	MHC class II, DQ beta 2	− 13.12	2.51E−150	5.93E−148	Down-regulated
DOB	MHC class II, DO beta chain	− 3.20	5.79E−08	1.23E−06	Down-regulated
GZMB	Granzyme B	− 2.07	4.20E−05	5.31E−04	Down-regulated
CD3D	CD3d molecule	− 1.71	7.54E−06	1.10E−04	Down-regulated
MS4A1	Membrane spanning 4-domains A1	− 1.53	2.37E−25	1.93E−23	Down-regulated
FAS	Tumor necrosis factor receptor superfamily member 6	− 1.53	6.47E−16	3.38E−14	Down-regulated
IgD	Immunoglobulin delta heavy chain constant region	− 1.49	5.11E−29	4.76E−27	Down-regulated
FCER1G	Fc fragment of IgE receptor Ig	− 1.43	3.13E−147	7.34E−145	Down-regulated
CSF1R	Colony stimulating factor 1 receptor	− 1.41	5.73E−22	4.21E−20	Down-regulated
TYROBP	TYRO protein tyrosine kinase-binding protein	− 1.40	4.39E−56	6.51E−54	Down-regulated
LCP2	Lymphocyte cytosolic protein 2	− 1.34	4.94E−06	7.49E−05	Down-regulated
CD48	CD48 molecule	− 1.30	8.85E−06	1.28E−04	Down-regulated
C4BPA	Complement component 4 binding protein, alpha	− 1.25	2.95E−05	3.84E−04	Down-regulated
CD2	CD2 molecule	− 1.23	4.16E−63	6.71E−61	Down-regulated
ITGA2B	Integrin subunit alpha 2b	− 1.22	9.49E−07	1.67E−05	Down-regulated
CD3E	CD3e molecule	− 1.08	1.21E−77	2.21E−75	Down-regulated

### Protein—protein interaction network of DEGs

**Figure 10:**
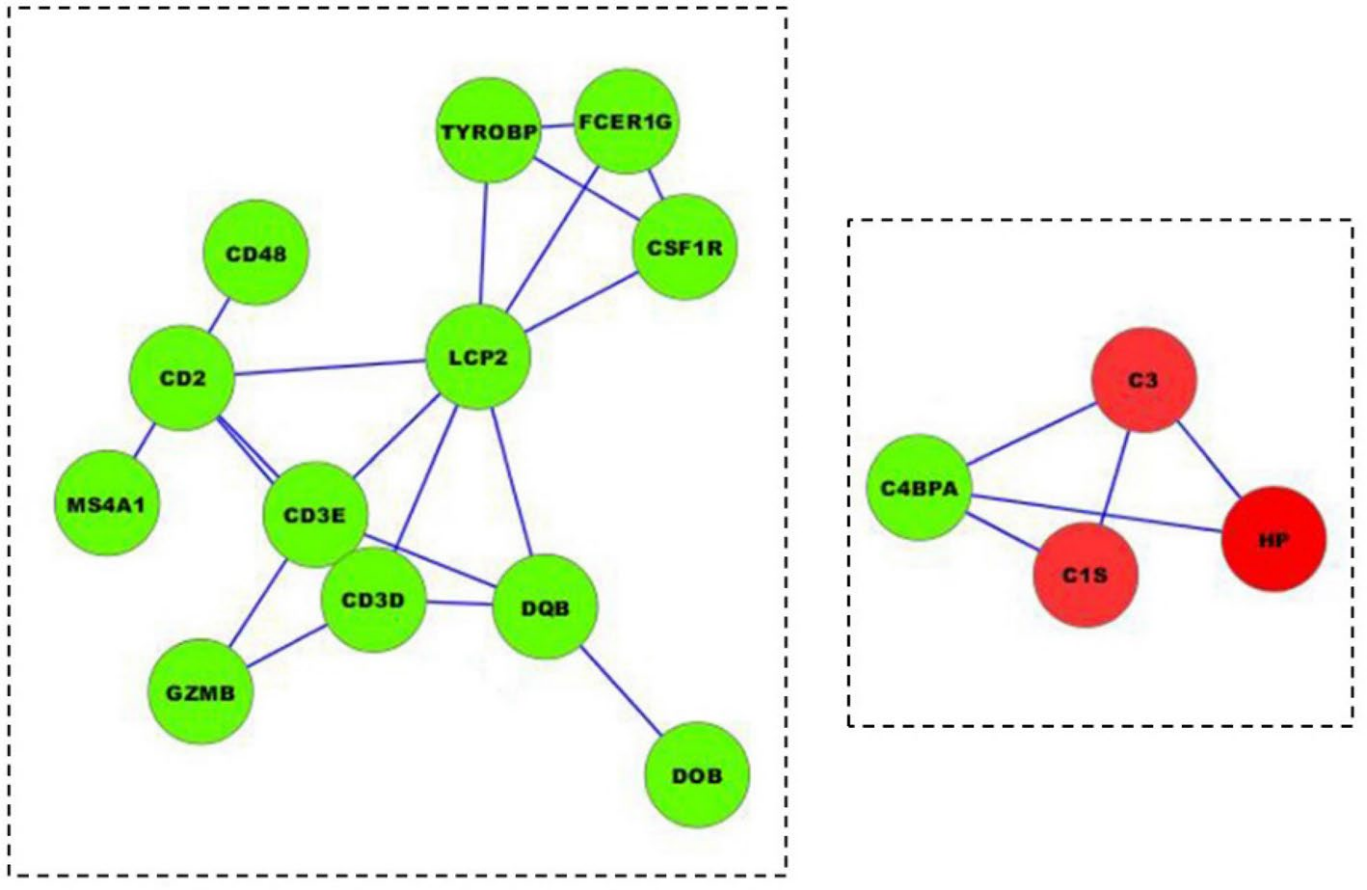
The protein–protein interaction networks of the significantly differentially expressed genes associated with the immune system. The sketch represents the interaction network constructed for protein-coding DEGs. Green nodes indicate down-regulated genes, and red nodes indicate up-regulated genes. The lines indicate the interactions between the gene products.

In this study, the STRING online tool was used to construct the PPI of 21 DEGs associated with the immune system. In total, 16 immune-responsive genes were built into two interaction networks using Cytoscape 2.8.2 software (Fig. 10). Of these 12 down-regulated genes (*TYROBP*, *FCER1G*, *CSF1R*, *LCP2*, *MS4A1*, *CD2*, *CD48*, *CD3D*, *CD3E*, *GZMB*, *DQB*, and *DOB*) existed in one interaction network related to hematopoietic cell lineage, natural killer cell mediated cytotoxicity, and the intestinal immune network for IgA production pathway, and four genes (*C2*, *HP*, *C1s*, and *C4BPA*) existed in the network related to the complement and coagulation cascade pathway. *C3*, *HP*, and *C1s* were up-regulated, and *C4BPA* was down-regulated.

### Validation of RNA-seq results by RT-qPCR

**Figure 11:**
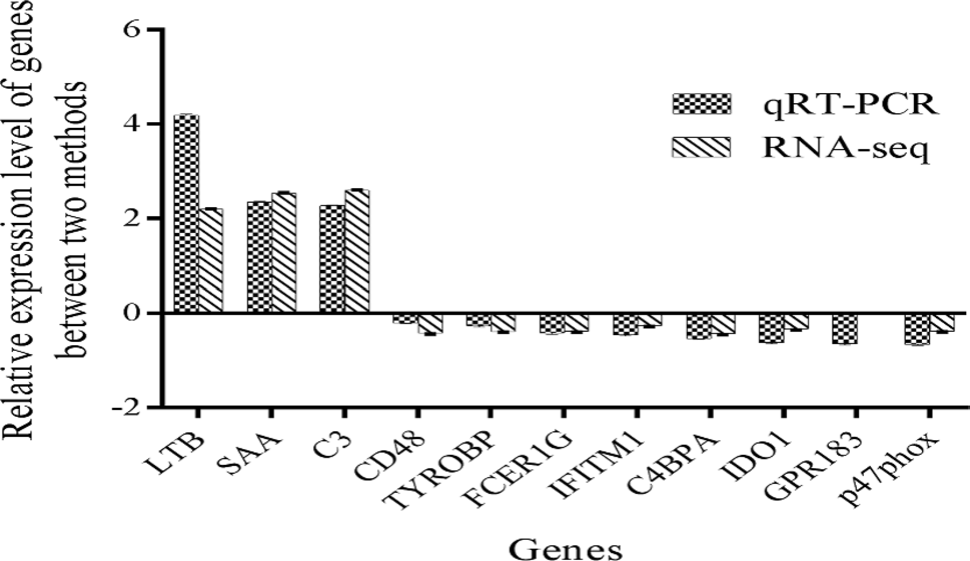
Validation of gene expression data using RT-qPCR.

To validate the RNA-seq results, the expression fold changes (2^−ΔΔCt^) of 11 DEGs were measured via RT-qPCR (Fig. 11). These genes belong to divergent functional categories. The serum amyloid A protein (*SAA*) are associated with the extracellular region; eight genes, including lymphotoxin beta (*LTB*), complement C3 (*C3*), CD48 antigen precursor (*CD48*), Fc fragment of IgE receptor Ig (*FCER1G*), complement component 4 binding protein, alpha chain precursor (*C4BPA*), indoleamine 2,3-dioxygenase 1 (*IDO1*), G-protein coupled receptor 183 (*GPR183*), and NADPH oxidase cytosolic protein p47phox (*p47phox*) are associated with immune system processes. Two genes, TYRO protein tyrosine kinase-binding protein (*TYROBP*) and interferon-induced transmembrane protein 1 (9–27) isoform 1 (*IFITM1*) are associated with membrane. The expression of 10 genes (*LTB*, *SAA*, *C3*, *CD48*, *TYROBP*, *FCER1G*, *IFITM1*, *C4BPA*, *IDO1,* and *p47phox*) obtained by RT-qPCR was similar to RNA-seq. The expression of *GPR183* found by RT-qPCR was similar, but not identical to RNA-seq (Fig. 11). These results suggest that the gene expression data from the RNA-seq experiments were valid.

## Discussion

Adaptation can be defined as the level of tolerance to survive and reproduce under extreme living conditions^21^. Sheep are an excellent model for researching the genetic mechanisms underlying the adaptation of livestock to extreme environments^13^, because they have spread and adapted to a wide range of agroecological conditions with different climates, including plateaus and desert regions, since their domestication in the Fertile Crescent^22^. Research shows that most adaptive traits are at least moderately heritable^23^, suggesting that animal breeding to improve adaptation is feasible.

In general, immune responses can be regulated by the immune factors related to animal adaptation to extreme environments^4^. According to previous studies, comparisons between exotic and local breeds of European sheep showed that the local Lacaune and Merino breeds had more resistance to natural infections with *T. circumcincta* than the imported, highly prolific Romanov sheep from the south of France^24^. Red Maasai sheep were more resistant (low FEC) and more resilient (high PCV) to gastro-intestinal (GI) nematode parasites than Dorper sheep in African sub-humid coastal environment^25^. Pelibuey sheep have been found to regulate body temperature after heat stress more efficiently than Suffolk sheep via a mechanism related to HSP-70^5^. On the Qinghai–Tibetan Plateau, Tibetan sheep have introgressed genomic regions related to the oxygen transportation system, sensory perception and morphological phenotypes from Argali (*Ovis ammon*), including *HBB* and *RXFP2* genes, which have strong signs of adaptive introgression^12^. Previous research on sheep has mainly focused on specific immunity against parasitic infections and genetic mechanisms of adaption to different environments; however, research on the relationship between adaptation and immunity is lacking. In this study, immunological differences and immune-responsive genes for adaptive traits from indigenous and exotic sheep breeds were identified and compared. The Kazakh and Suffolk sheep appeared to be healthy as evidenced by histomorphology observation of spleens. ELISA results showed that the levels of eight cytokines and IgG were higher in the blood of Kazakh compared with Suffolk sheep, and the levels of IgG and IL-1β were significantly higher in Kazakh than in Suffolk sheep. However, they grow slowly and have excessive fat-deposition. Suffolk sheep grow quickly and have excellent meat quality, but do not adapt to harsh environments and have lower levels of IgG and cytokines. Thus, Kazakh sheep may have better immunity and adapt better to arid and extremely hot environments compared with Suffolk sheep. These results indicate that indigenous adapted breeds tend to be small with poor production characteristics, whereas high-performing breeds often have poor disease-resistance characteristics. Native or indigenous livestock breeds are well adapted to their regions and are of historical and economic importance^26^, but intensive farming practices widely use improved or cross-bred animals for increased productivity and profit, which results in significant declines of native breeds^27^. Native breeds may be more adapted to a more stringent environment and climate, such as that of high mountains and northern coastal areas that are relatively marginal for agriculture^26^. Therefore, preservation of genetic diversity of native breeds can play key evolutionary roles in smart agriculture practices to dealing with global climate change and sustainable farming in the long term^26^.

To better understand the molecular mechanisms related to the immunity of adaptive traits in sheep, RNA-Seq techniques were employed and transcriptome sequencing for the DEG identifications was performed in spleen tissues from Suffolk and Kazakh sheep. A total of 1,158 significantly DEGs were identified between the two libraries, thus providing a valuable resource for immunogenetics and genomic research on sheep spleen tissue. Furthermore, 25 GO terms of DEGs were enriched in the cellular components, biological processes and molecular functions. Immune-related proteins were included in the biological processing category with higher percentages in immune system processes and response to stimuli. Within the molecular function category, the spleen transcripts were mainly associated with catalytic activity and binding, which represent some immune-related processes. The GO analysis followed a similar pattern to that obtained using pyrosequencing for defense mechanisms by injection with viral stimuli to increase the expression level of immune-related genes in Turbot^28^. These percentages suggest that a few immune-related genes were identified in this study.

Previous studies have reported that signaling pathways, such as toll-like receptor signaling pathway, Imd pathway, JAK/STAT pathway, JNK pathway^29^, TNF signaling pathway, B cell receptor signaling pathway, T cell receptor signaling pathway^30^, and NF-κB signaling pathway^31^, play an important role in immunity. In this study, comparison with the KEGG pathway database, showed that the toll-like receptor signaling pathway, JAK/STAT pathway, B cell receptor signaling pathway and T cell receptor signaling pathway were not significantly enriched; however, four immune system pathways were significantly enriched. Hematopoietic cell lineage refers to the differentiation and developmental processes of hematopoietic stem cells (HSCs), which divide into common myeloid progenitor cells by stimulation with IL-1, IL-3, IL-6, GM-CSF, and SCF. Cellular cytotoxicity is an important effector mechanism of the immune system to combat viral infections and is majorly mediated by cytotoxic T-cells and natural killer (NK) cells^32^. The complement system is a mediator of innate immunity and a nonspecific defense mechanism against pathogens. Complement and coagulation cascades may be implicated in the protective strategy of the brain for resistance to cold and environmental adaptation in the Himalayan marmot^33^. The intestine is the largest lymphoid tissue in the body, and intestinal immunity can generate many noninflammatory IgA antibodies, which are the first line of defense against microorganisms. Multiple cytokines, including TGF-β, IL-10, IL-4, IL-5, and IL-6, are necessary factors for IgA class switching and the terminal differentiation process in B cells^34^. The protein–protein interaction network of DEGs, the hematopoietic cell lineage, natural killer cell mediated cytotoxicity, and the intestinal immune network for IgA production pathway include 12 genes that coregulate the immune system in the spleen. The complement and coagulation cascades pathway includes four genes that regulate the immune system alone. These results indicate that hematopoietic cell lineage, natural killer cell mediated cytotoxicity, and the intestinal immune network for IgA production participate together in the regulation of the immune response. Results of this study suggest that the complement and coagulation cascade pathway likely plays an important role in the biological process of immune response and adaptation to arid and hot environments.

*DQA2*, *C1s*, *DQB*, *DOB*, and *GZMB* genes of the 21 DEGs associated with immunity were listed by the threshold of FDR ≤ 0.001 and fold change ≥ 2. *DQA2*, *DQB* and *DOB* showed significant enrichment in hematopoietic cell lineage and intestinal immune network for IgA production. *DQA* and *DQB* located in the MHC class IIa region, and *DOB*, located in MHC class IIb region, control self/non-self-recognition of the immune system^35^, and play important roles in immune response to adaptive trait. Granzyme B (*GZMB*) is significantly enriched in natural killer cell- mediated cytotoxicity and play an important role in the ability of NK cells and CD8+ T cells to kill their targets and modulate inflammation^36^. GZMB levels in plasma might reflect the degree of pruritus and dermatitis in patients with Alzheimer’s disease^37^. C1s is significantly enriched in complement and coagulation cascades, and can initiate the classical signaling pathway of complement activation and prevent of the formation and precipitation of large immune complexes^38^. In the current study, some genes were significantly down-regulated in Suffolk sheep, but up-regulated in Kazakh sheep, which had higher levels of blood IgG and cytokines, indicating that these genes might be conducive to adapting to arid and hot environment. *DQB*, *DOB*, *GZMB*, *CD3D*, *MS4A1*, *FCER1G*, *CSF1R*, *TYROBP*, *LCP2*, *CD48*, *CD2*, and *CD3E* (the 12 genes in the PPI network of hematopoietic cell lineage, natural killer cell- mediated cytotoxicity and the intestinal immune network for IgA production pathway), play roles in immune regulation. *C1s*, *HP,* and *C3* in PPI network of the complement and coagulation cascades were significantly up-regulated in Suffolk sheep, but down-regulated in Kazakh sheep, suggesting that living in harsh environments for a long time might be not conducive to growth and development due to a suppressed immune pathway. In summary, these findings, combined with previous results, clearly indicate that many spleen-specific DEGs play roles in immune function in indigenous and exotic sheep breeds with different adaptabilities. Future studies are needed to determine functional characterization of the immune-response genes associated with adaptation in sheep.

## Conclusion

In this study, the first comprehensive transcriptome sequencing and gene expression profiling analysis in spleen tissues based on content differences of IgG and cytokines between indigenous Kazakh sheep and exotic Suffolk sheep were performed. The results revealed the immunological and genetic differences associated with adaptation between indigenous and exotic sheep, with higher levels of blood IgG and cytokines in indigenous Kazakh sheep and 1,158 significantly differentially expressed genes in the spleens between two sheep breeds. Twenty-one immune-responsive genes were identified as spleen-specific genes associated with adaptive traits. Four pathways (hematopoietic cell lineage, natural killer cell mediated cytotoxicity, complement and coagulation cascades, and intestinal immune network for IgA production pathway) and up-regulated genes (including *DQB*, *DOB*, and *GZMB*) associated with immune response in indigenous sheep were helpful for adapting to harsh environments. Transcriptome and gene expression profiling data are valuable resources for future comparative genomic analysis on immunological mechanisms of adaptive trait and offer a foundation for research into genetic adaptive evolution.

## Materials and methods

### Animals and climatic conditions

The study was carried out in compliance with the ARRIVE guidelines. All animal experiments were carried out in accordance with ARRIVE guidelines published in 2010 (https://arriveguidelines.org), and were approved by the Institutional Animal Care and Use Committee of Xinjiang Academy of Agricultural and Reclamation Science. In this study, 159 sheep were randomly selected from a large population. The 94 imported Suffolk sheep consisted of 38 males and 56 females and the 65 indigenous Kazakh sheep consisted of 27 males and 38 females. All sheep were healthy, 7-months-old, fed a semi-grazing diet ad libitum and maintained under the same environmental, feeding, and management conditions for breeding farm flocks in the south edge of the Gurbantunggut desert in Xinjiang Uygur Autonomous Region of China (Latitude: 43° 26′–45° 20′ N; Longitude: 84° 58′–86° 24′ E; Altitude: 450.8 m above sea level. The region had an annual precipitation of 180–270 mm, annual evaporation of 1000–1500 mm, and annual temperature of –32.5–34.1 °C. The temperature range was 25–34.1 °C and average precipitation was 21.5 mm in July.

### Samples

Peripheral blood was collected by jugular venipuncture. Serum was collected after centrifugation (2500 rpm for 10 min), and transported to the laboratory at 4 °C for ELISA assays. Three male Suffolk sheep and three male Kazakh sheep were slaughtered, and samples of the parenchyma of the spleen were collected. One part of each sample was immediately frozen in liquid nitrogen and stored at  − 80 °C for subsequent total RNA extraction and another part was used for paraffin sections.

### Hematoxylin–eosin (HE) staining of spleen tissues

The spleen tissues were treated for histological imaging using standard H&E staining procedures. Spleens were fixed in freshly prepared 4% paraformaldehyde solution in PBS for 18 h. Spleens were then washed with PBS, dehydrated in gradient ethanol, made transparent with xylene, and embedded in paraffin wax. The wax blocks were fixed on a slicer and continuously sectioned at a thickness of 4 μm using a Leica CM1900 cryostat (Leica Microsystems, Wetzlar, Germany). Sections were dried at 37 °C, stained with a neutral resin, and observed under a microscope.

### ELISA assays

Serum ELISA was conducted to determine levels of IgG and eight cytokines, including interferon α (IFN-α), interferon γ (IFN-γ), interleukin (IL)-1β, IL-2, IL-5, IL-6, IL-8, and IL-12 using a commercially available ovine specific enzyme-linked immunosorbent assay (ELISA; BlueGene Biotech, Shanghai, China), according to the manufacturer’s instructions (Catalogue number: E014I0058, E14I0343, E14I0345, E14I0010, E14I0308, E14I0024, E014I0006, E14I0056 and E14I0033). The levels of serum IgG and cytokines were statistically analyzed via Student’s *t* tests using SPSS 21.0 (IBM, Armonk, NY, USA). *P* value < 0.05 were considered significant.

### RNA extraction and quality assessment

Total RNA was isolated from spleen tissues using TRIzol reagent according to the manufacturer’s instructions (Invitrogen, Carlsbad, CA, USA). The extracted RNA was treated with RNase-free DNase I (Ambion, Inc., Austin, TX, USA) for 30 min at 37 °C to eliminate contamination by genomic DNA during RNA isolation. RNA purity and yield were determined by measuring the absorbance at A260/A280 with NanoDrop ND-2000 spectrophotometer (Nano-Drop Technologies, Wilmington, DE, USA). Total RNA quality was evaluated by electrophoresis through a 1.2% (w/v) agarose gel. The RNA integrity number (RIN) was evaluated by using an Agilent RNA Nano Chip kit on an Agilent 2100 Bioanalyzer (Agilent Technologies, Inc., Santa Clara, CA, USA), and RNA samples with a RIN > 7.5 were used for sequencing.

### Transcriptome sequencing and bioinformatics analysis

Illumina sequencing libraries were performed at Beijing Genomics Institute (BGI, Shenzhen, China) using the Illumina Truseq RNA Sample Preparation Kit (Illumina, San Diego, CA, USA). The mRNA was enriched from total RNA using oligo (dT) magnetic beads (Invitrogen, Carlsbad, CA, USA). The purified mRNA was pooled, and fragmentation buffer was added to disrupt the mRNA into short fragments of about 200 bp. A random hexamer primer was added to synthesize the first strand cDNA using the short fragments as templates. Buffer, dNTPs, RNase H, and DNA polymerase I were sequentially added to synthesize the second strand cDNA. The double-stranded cDNA was subsequently purified using a QiaQuick PCR extraction kit and resolved with elution buffer for end repair and addition of a poly (A) tail. Illumina sequencing adaptors were then ligated to the short fragments. The suitable fragments were purified by agarose gel electrophoresis and enriched using PCR amplification as templates. The average insert size for the paired-end libraries was 200 bp. Finally, two paired-end cDNA libraries were constructed and used for sequencing analysis on the Illumina HiSeq 2000 system.

The imaging data output was transformed by base calling into raw reads and stored in FASTQ format. All clean reads went through a stringent filtering process. For obtaining clean reads, the program removes raw reads, reads with adaptors, and reads in which unknown bases were more than 10%, and low-quality reads (i.e. the percentage of the low quality bases of quality value ≤ 5 was more than 50% in a read). Transcriptome de novo assembly was carried out with short reads using Trinity software packages^39^. After combined reads with overlaps formed contigs, Trinity was used to connect the contigs to obtain sequences that could not be extended on either end. These contigs are subjected to further processing of sequence clustering to form longer sequences without N. Such sequences were defined as unigenes. Basically, unigenes are a collection of expressed sequences that are aligned or located to same position on the genome. The unigenes were annotated using the NR, NT, SwissProt, KEGG, COG, and Gene ontology (GO) databases and the number of annotated unigenes counted.

### Expression profiling and analysis of DEGs

Digital gene expression profiling (DGE) was performed at Beijing Genomics Institute (BGI, Shenzhen, China) using the Illumina Gene Expression Sample Prep Kit and Illumina sequencing chip (Illumina, San Diego, CA, USA) according to the manufacturer’s instructions.

The procedure was modified from a published method^40^. The Illumina Cluster Station and Illumina HiSeq 2000 System were used. Experimental processes were as follows: mRNA was purified from 6 µg total RNA by using Oligo(dT) magnetic beads and the first and second-strand cDNA were synthesized using Oligo(dT) as primer. The 5′ ends of tags generated the NlaIII endonuclease site and the bead-bound cDNA was subsequently digested with restriction enzyme NlaIII, which recognized and cut off the CATG sites. The fragments separated from the 3′ cDNA fragments and connected to Oligo(dT) beads were washed away and the Illumina adaptor 1 was ligated to the sticky 5′ end of the digested bead-bound cDNA fragments. The junction of the adaptor 1 and CATG site was the recognition site of MmeI, which was cut at 17 bp downstream of the CATG site, producing tags with adaptor 1. After removing the 3′ fragments via magnetic bead precipitation, the Illumina adaptor 2 was ligated to the 3′ ends of the tags to acquire tags with different adaptors of both ends to form a tag library. After 15 cycles of linear PCR amplification, 105 bp fragments were purified using 6% TBE PAGE gel electrophoresis. After denaturation, the single-chain molecules were fixed onto the Illumina sequencing chip. Each molecule grew into a single-molecule cluster sequencing template through situ amplification. Then, four types of nucleotides labeled using four colors, were added, and the sequencing by synthesis (SBS) method was performed. Each tunnel generated millions of raw reads with sequencing lengths of 49 bp.

Sequencing-received raw image data were transformed via base calling into raw data, which were stored in FASTQ format. Raw sequences were transformed into clean tags by a stringent filtering process: 3′ adaptor sequences, empty reads, low quality tags with unknown sequences (N), and tags with a copy number of 1 were removed leaving tags 21 nt long. Sequence saturation analyses and experimental repeatability analysis of the two libraries were performed to evaluate whether the number of detected genes increased as the amount of sequencing and operational stability increased. De novo transcriptome and sheep genome (OARv3.1) were used as the reference sequence, in which all clean tags were mapped to the reference sequences using SOAP2^41^. The remaining clean tags were designed as unambiguous clean tags by filtering the clean tags mapped to reference sequences from multiple genes. The number of unambiguous clean tags for each gene was calculated and then normalized to TPM (number of transcripts per million clean tags)^42^. TPM of ≥ 0.1 was used as the threshold value of gene expression. The DEGs between the two libraries were identified using a rigorous algorithm^43^, and the false discovery rate (FDR) of ≤ 0.001 and the absolute value of log_2_Ratio of ≥ 1 (fold change between Suffolk and Kazakh library) were used as the significance threshold of the *P* value to judge the significance of differences in gene expression^44^.

### GO and KEGG enrichment analysis of DEGs

BLAST2GO Ver.2.6.5 was used for functional annotation and analysis of DEGs according to molecular function, biological process and cellular component ontologies. WEGO Ver.2.0 was employed to perform GO functional classifications for all genes and plotting of GO annotations^45^. The GO distributions were statistically analyzed using a threshold of Bonferroni-corrected *P* value of ≤ 0.05. Kyoto Encyclopedia of Genes and Genomes (KEGG) pathway annotation was conducted using the DAVID website (http://david.abcc.ncifcrf.gov/) and searching the KEGG database. DEGs were loaded to the KEGG Automatic Annotation Server (KAAS) Ver. 2.1 (https://www.genome.jp/tools/kaas/)^46^. The functional annotation of genes was carried out by BLAST against the manually curated KEGG GENES database, including immune system and environmental adaptation pathways. The KEGG pathway with *P* ≤ 0.05 was selected as the enrichment biological pathway. The immune molecules were marked in background and foreground colors in the Search & Color Pathway of the KEGG mapping tool^47,48^.

### Protein—protein interaction network

The STRING Ver.11.0 (http://string-db.org/) database was used to construct a potential protein–protein interaction (PPI) network between proteins encoded by DEGs related to immunity. The PPI data obtained from the STRING database was imported into the Cytoscape software, and the nodes were used as a network center node.

### Validation of RNA-Seq data using quantitative real-time PCR (RT-qPCR)

Eleven candidate genes were selected and detected by using RT-qPCR. The primers for the 11 genes were designed using Primer-BLAST^49^ (Table 5). The amplification product of the expected size for each gene was verified by electrophoresis on a 2.0% agarose gel and confirmed by sequencing. A quantity of 2 μg total RNA was used to synthesize first strand cDNA using the PrimeScript RT reagent Kit with gDNA Eraser (TaKaRa Biotech, Dalian, China), according to the manufacturer’s instructions. The RT-qPCR reactions were performed in a 20 μL volume containing 10 μL 2 × LightCycler 480 SYBR Green I Master (Roche Diagnostics, Mannheim, Germany), 2 μL cDNA, 0.5 μM of each primer, and 7 μL ddH2O. The amplification conditions were as follows: 95 °C for 5 min of initial stage, followed by 40 cycles at 95 °C for 10 s, annealing temperature for 10 s, and 72 °C for 20 s, performed on the LightCycler 480II system (Roche Diagnostics GmbH, Mannheim, Germany). The *GAPDH* gene was used as an internal control. All RT-qPCR reactions were carried out in triplicate, with both technical and biological replicates. RNA samples from six Suffolk sheep and six indigenous Kazakh sheep were used for this analysis. Values of RT-qPCR were calculated by 2^−ΔΔCt^ method and data were analyzed with IBM SPSS 24.0^50^.

**Table 5:** The primers of genes in RT-qPCR analysis.

Gene	Primer sequence (5′–3′)	Annealing temperature (°C)
LTB	F: CAGCGATTTGAGTCTGGGGA, R: GGTAGCCGACGTGACAGTAG	61
SAA	F: AGCAGGTCACAGCCAAAGGAT, R: TCCACATGTCTTTAGCCCCTTGAC	61
C3	F: GCAATCAGGCAGCGACATGG, R: CACGTACACCATGAGGTCGAA	61
CD48	F: ACCCTGTGTACAAGCCTCCT, R: TCTGTCCTGGTTAAGAGCCG	62
TYROBP	F: GACTGTGGGTGGTCTCAGC, R: AGTACACAGCCAGAGCGATG	62
FCER1G	F: TACTGCCGACTCAAGCTGC, R: AGCTACTGTGGCGGTTTCTC	50
IFITM1	F: TGTGGTCCCTGTTCAACACC, R: CCAGATGTTCAGGCACTTGG	57
C4BPA	F: TAGTCTCTAAAGGCTCGTGGT, R: ACACACCGTGCTTTACACTGA	60
IDO1	F: CGAATATACTTGTCTGGTTGG, R: GGAGAACATCAAAGCACTG	55
GPR183	F: CTGAGAGAGACCCTGAACAAC, R: CGATGCTGTAATGCAGTGGC	60
p47phox	F: GCCATCGCTGACTACGAGAA, R: GTAGGGCTCACCTGCATAGT	60
GAPDH	F: CTGACCTGCCGCCTGGAGAAA, R: GTAGAAGAGTGAGTGTCGCTGTT	61

### Ethics declarations

The Guidelines for the Care and Use of Laboratory Animals were carefully followed during this study, which received approval from the Experimental Animal Care and Use Committee of Xinjiang Academy of Agricultural and Reclamation Sciences (Shihezi, China, approval number: XJNKKXY-AEP-039, 22 January 2012), and was approved by the Institutional Animal Care and Use Committee of University of Hawaii under the protocol No. 10-1039-2 and the Animal Welfare Assurance No. A3423-01. All efforts were made to follow the animal care and sampling procedures to minimize animal suffering.

## Supplementary Information

Supplementary Table.

## Data Availability

The datasets generated or analyzed during the current study are available in the Genome Sequence Archive in National Genomics Data Center, Beijing Institute of Genomics (China National Center for Bioinformation), Chinese Academy of Sciences, under accession number CRA002739, https://bigd.big.ac.cn/gsa.
