# Supplementary material for: Identifications of immune-responsive genes for adaptative traits by comparative transcriptome analysis of spleen tissue from Kazakh and Suffolk sheep
Source: Sci Rep. 2021 Feb 4;11:3157. doi: 10.1038/s41598-021-82878-x (PMC7862382; doi:10.1038/s41598-021-82878-x)
Supplement: Supplementary file 1 — Supplementary Table. [file 41598_2021_82878_MOESM1_ESM.docx]

**I****d****e****ntifications of** **immune-responsive genes for adaptative traits by comparative** **transcriptome analysis of spleen tissue from Kazakh and Suffolk sheep**

Hua Yang^1,2^, Yong-Lin Yang^1,2^, Guo-Qing Li^1,2^, Qian Yu^1,2^, Jinzeng Yang^3*^

^1^State Key Laboratory of Sheep Genetic Improvement and Healthy Production, Shihezi, 832000, China; ^2^Institute of Animal Husbandry and Veterinary Medicine, Xinjiang Academy of Agricultural and Reclamation Science, Shihezi, 832000, China; ^3^Department of Human Nutrition, Food and Animal Sciences, University of Hawaii, Honolulu, HI 96822, USA

*Corresponding author: E-mail address: [jinzeng@hawaii.edu](mailto:jinzeng@hawaii.edu) （Jinzeng Yang）

**Supplemental Materials**

**Table S1. Significantly DEGs between two libraries of spleen tissue from Kazakh and Suffolk sheep**

| Gene | TPM-K | TPM-S | log2 Ratio(S/K) | P-Value | FDR | Gene name* |
| --- | --- | --- | --- | --- | --- | --- |
| Unigene26547_S2A | 0.01 | 479.99 | 15.55 | 2.22E-16 | 1.19E-14 | - |
| CL435.Contig2_S2A | 0.01 | 43.96 | 12.10 | 2.22E-16 | 1.18E-14 | - |
| CL85.Contig1_S2A | 0.01 | 16.65 | 10.70 | 2.22E-16 | 1.18E-14 | Proteoglycan 3 precursor |
| Unigene59282_S2A | 0.01 | 16.10 | 10.65 | 2.22E-16 | 1.19E-14 | - |
| CL3760.Contig1_S2A | 0.01 | 11.76 | 10.20 | 2.22E-16 | 1.18E-14 | - |
| Unigene14866_S2A | 0.01 | 9.95 | 9.96 | 2.22E-16 | 1.19E-14 | Serum albumin precursor |
| Unigene25227_S2A | 0.01 | 9.23 | 9.85 | 2.22E-16 | 1.19E-14 | - |
| Unigene80567_S2A | 0.01 | 7.96 | 9.64 | 2.53E-14 | 1.16E-12 | - |
| Unigene81015_S2A | 0.01 | 7.42 | 9.54 | 2.13E-13 | 8.71E-12 | - |
| Unigene11734_S2A | 0.01 | 6.69 | 9.39 | 3.67E-12 | 1.30E-10 | Asc-type amino acid transporter 1 |
| Unigene32593_S2A | 0.01 | 6.33 | 9.31 | 1.52E-11 | 5.03E-10 | - |
| Unigene37264_S2A | 0.01 | 5.43 | 9.08 | 5.33E-10 | 1.46E-08 | - |
| CL4797.Contig2_S2A | 0.01 | 4.52 | 8.82 | 1.87E-08 | 4.28E-07 | - |
| Unigene27064_S2A | 0.01 | 4.34 | 8.76 | 3.80E-08 | 8.28E-07 | - |
| Unigene13331_S2A | 0.01 | 3.98 | 8.64 | 1.58E-07 | 3.15E-06 | - |
| Unigene76655_S2A | 0.01 | 3.80 | 8.57 | 3.21E-07 | 6.18E-06 | Reductase SDR family member 12 |
| Unigene41953_S2A | 0.01 | 3.80 | 8.57 | 3.21E-07 | 6.17E-06 | - |
| Unigene37503_S2A | 0.01 | 3.44 | 8.43 | 1.33E-06 | 2.27E-05 | - |
| CL270.Contig1_S2A | 0.01 | 3.26 | 8.35 | 2.71E-06 | 4.35E-05 | - |
| Unigene75774_S2A | 0.01 | 3.26 | 8.35 | 2.71E-06 | 4.35E-05 | 28S ribosomal protein S35, mitochondrial precursor |
| Unigene80021_S2A | 0.01 | 3.08 | 8.27 | 5.52E-06 | 8.28E-05 | - |
| Unigene25554_S2A | 0.01 | 2.71 | 8.08 | 2.29E-05 | 3.07E-04 | - |
| Unigene17972_S2A | 0.01 | 2.53 | 7.98 | 4.66E-05 | 5.83E-04 | Long-chain fatty acid transport protein 6 |
| Unigene26688_S2A | 0.01 | 2.53 | 7.98 | 4.66E-05 | 5.83E-04 | NADH dehydrogenase [ubiquinone] 1 beta subcomplex subunit 8, mitochondrial |
| Unigene62044_S2A | 0.01 | 2.53 | 7.98 | 4.66E-05 | 5.83E-04 | - |
| Unigene11605_S2A | 0.87 | 19.00 | 4.45 | 3.02E-05 | 3.92E-04 | - |
| Unigene25703_S2A | 4.54 | 68.39 | 3.91 | 1.08E-10 | 3.23E-09 | Alpha globin chain |
| Unigene25289_S2A | 5.59 | 78.34 | 3.81 | 2.85E-12 | 1.02E-10 | Mannosyl (alpha-1,3-)-glycoprotein beta-1,2-N-acetylglucosaminyltransferase-like, partial |
| Unigene35180_S2A | 1.57 | 17.73 | 3.50 | 3.42E-06 | 5.38E-05 | ARV1 protein |
| CL3480.Contig1_S2A | 733.25 | 7242.79 | 3.30 | 1.95E-11 | 6.35E-10 | Immunoglobulin lambda-2b light chain variable region |
| Unigene73956_S2A | 0.70 | 6.88 | 3.30 | 4.93E-05 | 6.13E-04 | Pentraxin-related protein PTX3 precursor |
| Unigene51982_S2A | 0.52 | 4.88 | 3.23 | 8.04E-05 | 9.44E-04 | - |
| CL3025.Contig1_S2A | 1.57 | 14.47 | 3.20 | 3.42E-06 | 5.38E-05 | - |
| Unigene26974_S2A | 19.38 | 172.96 | 3.16 | 0 | 0 | Immunoglobulin alpha heavy chain |
| Unigene10067_S2A | 0.87 | 7.24 | 3.06 | 3.02E-05 | 3.92E-04 | HCG1647254-like |
| CL944.Contig1_S2A | 4.36 | 35.82 | 3.04 | 2.01E-10 | 5.84E-09 | - |
| Unigene41981_S2A | 1.22 | 9.95 | 3.03 | 1.05E-05 | 1.49E-04 | Gamma-aminobutyric acid receptor subunit delta precursor |
| CL15.Contig1_S2A | 1.92 | 14.84 | 2.95 | 1.07E-06 | 1.87E-05 | Helicase SRCAP |
| Unigene26623_S2A | 48.88 | 360.76 | 2.88 | 0 | 0 | Plasma cell-induced resident endoplasmic reticulum protein precursor |
| Unigene922_S2A | 7.16 | 52.11 | 2.86 | 1.50E-13 | 6.30E-12 | MHC class II alpha domain |
| Unigene76566_S2A | 4.71 | 34.19 | 2.86 | 5.79E-11 | 1.79E-09 | - |
| Unigene57035_S2A | 1.92 | 13.57 | 2.82 | 1.07E-06 | 1.87E-05 | - |
| Unigene35311_S2A | 11.00 | 76.89 | 2.81 | 0 | 0 | - |
| Unigene81246_S2A | 3.84 | 26.05 | 2.76 | 1.30E-09 | 3.44E-08 | - |
| Unigene11864_S2A | 2.27 | 15.38 | 2.76 | 3.27E-07 | 6.29E-06 | B-cell lymphoma 3 protein |
| Unigene26333_S2A | 1.05 | 7.06 | 2.75 | 1.81E-05 | 2.48E-04 | Complement C1s subcomponent |
| Unigene19225_S2A | 0.70 | 4.70 | 2.75 | 7.15E-05 | 8.52E-04 | - |
| Unigene71971_S2A | 7.68 | 50.12 | 2.71 | 7.93E-14 | 3.45E-12 | - |
| Unigene66614_S2A | 1.40 | 8.68 | 2.63 | 6.04E-06 | 8.97E-05 | - |
| Unigene888_S2A | 26.01 | 159.39 | 2.62 | 1.06E-13 | 4.59E-12 | - |
| CL3431.Contig1_S2A | 34.39 | 210.42 | 2.61 | 0 | 0 | Angiogenin-2 precursor |
| Unigene66268_S2A | 7.51 | 43.78 | 2.54 | 1.14E-13 | 4.88E-12 | - |
| Unigene26216_S2A | 2.79 | 15.92 | 2.51 | 5.32E-08 | 1.14E-06 | Spermidine synthase-like |
| Unigene26793_S2A | 1.22 | 6.69 | 2.46 | 1.23E-05 | 1.73E-04 | ATPase inhibitor, mitochondrial precursor |
| Unigene393_S2A | 6.46 | 34.56 | 2.42 | 2.54E-13 | 1.03E-11 | TAF4 RNA polymerase II, TATA box binding protein (TBP)-associated factor |
| Unigene56273_S2A | 3.49 | 18.64 | 2.42 | 4.52E-09 | 1.13E-07 | - |
| Unigene11537_S2A | 2.27 | 11.58 | 2.35 | 3.28E-07 | 6.29E-06 | Solute carrier family 35 member B1 |
| Unigene26921_S2A | 1.40 | 7.06 | 2.33 | 7.87E-06 | 1.14E-04 | DnaJ homolog subfamily C member 3 precursor |
| CL1400.Contig1_S2A | 2529.35 | 12670.72 | 2.32 | 0 | 0 | IGK protein |
| Unigene12999_S2A | 1.22 | 5.97 | 2.29 | 2.59E-05 | 3.42E-04 | Fructose-bisphosphate aldolase B |
| Unigene11673_S2A | 6.28 | 30.58 | 2.28 | 3.73E-13 | 1.49E-11 | Protein ERGIC-53 precursor |
| Unigene66223_S2A | 7.33 | 35.28 | 2.27 | 1.10E-13 | 4.73E-12 | - |
| Unigene60071_S2A | 2.27 | 10.86 | 2.26 | 3.33E-07 | 6.36E-06 | Resistin precursor |
| Unigene26229_S2A | 2.09 | 9.95 | 2.25 | 6.22E-07 | 1.13E-05 | Zinc finger protein 343 |
| Unigene73532_S2A | 1.75 | 8.32 | 2.25 | 2.31E-06 | 3.74E-05 | - |
| Unigene69369_S2A | 1.22 | 5.79 | 2.25 | 3.67E-05 | 4.72E-04 | - |
| Unigene26371_S2A | 2.44 | 11.58 | 2.25 | 1.81E-07 | 3.59E-06 | - |
| Unigene66862_S2A | 1.22 | 5.61 | 2.20 | 5.48E-05 | 6.74E-04 | Tubulin polyglutamylase TTLL4 |
| Unigene26959_S2A | 10.30 | 46.86 | 2.19 | 2.80E-14 | 1.28E-12 | Pancreatic trypsin inhibitor |
| Unigene10430_S2A | 4.71 | 21.35 | 2.18 | 5.79E-11 | 1.79E-09 | - |
| Unigene11399_S2A | 3.84 | 17.37 | 2.18 | 1.30E-09 | 3.44E-08 | Centromere protein B-like |
| Unigene43186_S2A | 1.92 | 8.68 | 2.18 | 1.45E-06 | 2.45E-05 | - |
| CL694.Contig1_S2A | 88.86 | 394.96 | 2.15 | 0 | 0 | Anti-testosterone antibody |
| Unigene36125_S2A | 8.55 | 37.99 | 2.15 | 3.97E-14 | 1.79E-12 | DnaJ homolog subfamily B member 9 precursor |
| Unigene63494_S2A | 1.40 | 6.15 | 2.14 | 3.15E-05 | 4.08E-04 | - |
| Unigene13222_S2A | 1.40 | 5.97 | 2.09 | 4.86E-05 | 6.06E-04 | Hypothetical protein EGM_15683 |
| Unigene3550_S2A | 2.44 | 10.31 | 2.08 | 2.53E-07 | 4.91E-06 | Endonuclease reverse transcriptase |
| Unigene25383_S2A | 2.97 | 12.30 | 2.05 | 3.40E-08 | 7.46E-07 | Nucleoside diphosphate kinase A 1 |
| Unigene25486_S2A | 483.24 | 1995.78 | 2.05 | 0 | 0 | Cochlin precursor |
| CL630.Contig1_S2A | 3.14 | 12.85 | 2.03 | 1.86E-08 | 4.27E-07 | - |
| Unigene11202_S2A | 4.71 | 19.18 | 2.03 | 5.83E-11 | 1.80E-09 | - |
| Unigene35347_S2A | 1.75 | 7.06 | 2.01 | 1.56E-05 | 2.16E-04 | Coiled-coil domain containing 75 |
| Unigene11773_S2A | 6.28 | 25.33 | 2.01 | 3.73E-13 | 1.49E-11 | - |
| CL1830.Contig1_S2A | 13.97 | 56.27 | 2.01 | 0 | 0 | U6 snRNA-associated Sm-like protein LSm6 |
| CL4671.Contig1_S2A | 2.44 | 9.77 | 2.00 | 5.05E-07 | 9.38E-06 | Phospholipid transfer protein precursor |
| Unigene18252_S2A | 1.75 | 6.88 | 1.98 | 2.44E-05 | 3.24E-04 | - |
| Unigene35270_S2A | 11.35 | 44.51 | 1.97 | 0 | 0 | - |
| Unigene65823_S2A | 2.27 | 8.68 | 1.94 | 2.81E-06 | 4.49E-05 | Dipeptidase 3-like |
| Unigene81501_S2A | 1.75 | 6.69 | 1.93 | 3.87E-05 | 4.94E-04 | - |
| Unigene59194_S2A | 2.09 | 7.96 | 1.93 | 7.73E-06 | 1.12E-04 | - |
| Unigene25672_S2A | 7.86 | 29.49 | 1.91 | 2.55E-14 | 1.17E-12 | - |
| CL1648.Contig1_S2A | 1.75 | 6.51 | 1.90 | 6.16E-05 | 7.50E-04 | Ig kappa chain |
| Unigene4404_S2A | 1.75 | 6.51 | 1.90 | 6.16E-05 | 7.49E-04 | - |
| Unigene36666_S2A | 5.06 | 18.82 | 1.90 | 2.34E-11 | 7.59E-10 | Cartilage-associated protein precursor |
| CL3161.Contig1_S2A | 11.17 | 41.25 | 1.88 | 0 | 0 | - |
| Unigene60250_S2A | 17.98 | 65.49 | 1.86 | 0 | 0 | Transferrin |
| Unigene73439_S2A | 5.06 | 18.27 | 1.85 | 4.35E-11 | 1.37E-09 | - |
| Unigene55038_S2A | 1.92 | 6.88 | 1.84 | 5.47E-05 | 6.73E-04 | Spondin 2, extracellular matrix protein |
| CL614.Contig1_S2A | 4.36 | 15.56 | 1.84 | 1.24E-09 | 3.27E-08 | Zinc finger protein with KRAB and SCAN domains 1 isoform 1 |
| Unigene77167_S2A | 4.89 | 17.01 | 1.80 | 3.29E-10 | 9.26E-09 | - |
| Unigene26296_S2A | 38.06 | 131.53 | 1.79 | 0 | 0 | Von Willebrand factor |
| Unigene25328_S2A | 5.76 | 19.90 | 1.79 | 1.26E-11 | 4.21E-10 | Pre-B lymphocyte 3-like |
| Unigene11311_S2A | 31.25 | 107.65 | 1.78 | 0 | 0 | Transcription initiation factor TFIID subunit 4 |
| CL3489.Contig2_S2A | 2.79 | 9.59 | 1.78 | 2.95E-06 | 4.71E-05 | Serologically defined breast cancer antigen 84 isoform b |
| Unigene27050_S2A | 3.32 | 11.40 | 1.78 | 3.31E-07 | 6.33E-06 | - |
| Unigene60106_S2A | 2.27 | 7.78 | 1.78 | 2.68E-05 | 3.53E-04 | - |
| Unigene58859_S2A | 7.51 | 25.33 | 1.75 | 1.51E-13 | 6.35E-12 | - |
| Unigene11294_S2A | 2.44 | 8.14 | 1.74 | 2.35E-05 | 3.13E-04 | Endoplasmic reticulum-Golgi intermediate compartment protein 1-like, partial |
| Unigene71705_S2A | 4.02 | 13.21 | 1.72 | 7.99E-08 | 1.67E-06 | Signal recognition particle 54 kDa protein-like |
| Unigene59480_S2A | 3.14 | 10.31 | 1.72 | 2.26E-06 | 3.68E-05 | Chromobox protein homolog 5 |
| CL2055.Contig2_S2A | 7.68 | 25.15 | 1.71 | 2.06E-13 | 8.41E-12 | Galectin-14 |
| Unigene11962_S2A | 4.71 | 15.38 | 1.71 | 7.96E-09 | 1.94E-07 | Nucleobindin-2 precursor |
| Unigene36543_S2A | 3.84 | 12.48 | 1.70 | 2.22E-07 | 4.34E-06 | - |
| Unigene25654_S2A | 6.46 | 20.99 | 1.70 | 1.68E-11 | 5.51E-10 | Signal peptidase complex catalytic subunit SEC11C |
| Unigene65254_S2A | 2.79 | 8.68 | 1.64 | 2.76E-05 | 3.62E-04 | - |
| Unigene25366_S2A | 56.56 | 175.68 | 1.64 | 0 | 0 | - |
| Unigene66476_S2A | 3.32 | 10.31 | 1.63 | 4.75E-06 | 7.24E-05 | Midkine |
| Unigene26046_S2A | 5.76 | 17.73 | 1.62 | 2.23E-09 | 5.74E-08 | Apolipoprotein E |
| Unigene65892_S2A | 11.00 | 33.83 | 1.62 | 0 | 0 | Oncoprotein-induced transcript 3 protein |
| CL2002.Contig1_S2A | 4.19 | 12.85 | 1.62 | 4.00E-07 | 7.54E-06 | - |
| Unigene54107_S2A | 8.03 | 24.61 | 1.62 | 2.09E-12 | 7.69E-11 | - |
| Unigene6062_S2A | 2.62 | 7.96 | 1.60 | 7.69E-05 | 9.07E-04 | - |
| Unigene66257_S2A | 33.35 | 101.32 | 1.60 | 0 | 0 | Gamma-glutamyl hydrolase precursor |
| Unigene60277_S2A | 7.51 | 22.80 | 1.60 | 1.71E-11 | 5.63E-10 | - |
| Unigene26981_S2A | 13.44 | 40.71 | 1.60 | 0 | 0 | Metallothionein-2 |
| Unigene9322_S2A | 4.54 | 13.75 | 1.60 | 1.93E-07 | 3.80E-06 | - |
| Unigene58247_S2A | 17.28 | 51.56 | 1.58 | 4.44E-16 | 2.34E-14 | - |
| CL5148.Contig2_S2A | 8.90 | 26.23 | 1.56 | 1.47E-12 | 5.49E-11 | Cytochrome b561 domain-containing protein 2 |
| Unigene12100_S2A | 10.47 | 30.21 | 1.53 | 2.09E-14 | 9.64E-13 | Insulin-like growth factor-binding protein 6 precursor |
| Unigene71942_S2A | 17.81 | 51.38 | 1.53 | 0 | 0 | 39S ribosomal protein L4, mitochondrial |
| Unigene3752_S2A | 4.54 | 13.03 | 1.52 | 1.04E-06 | 1.82E-05 | - |
| Unigene65477_S2A | 8.03 | 22.80 | 1.51 | 1.30E-10 | 3.86E-09 | - |
| Unigene71191_S2A | 4.02 | 11.40 | 1.50 | 5.90E-06 | 8.79E-05 | P53-induced protein-like |
| Unigene26286_S2A | 16.93 | 47.58 | 1.49 | 3.91E-14 | 1.77E-12 | Ribosome-binding protein 1 |
| Unigene11266_S2A | 3.67 | 10.31 | 1.49 | 1.89E-05 | 2.58E-04 | - |
| CL4539.Contig1_S2A | 4.71 | 13.21 | 1.49 | 1.33E-06 | 2.27E-05 | Sugar transporter SWEET1-like |
| CL4894.Contig2_S2A | 3.49 | 9.77 | 1.49 | 3.38E-05 | 4.36E-04 | GTP-binding protein SAR1a |
| Unigene10826_S2A | 12.74 | 35.28 | 1.47 | 0 | 0 | Transmembrane protein 176A |
| Unigene14852_S2A | 4.19 | 11.58 | 1.47 | 7.51E-06 | 1.09E-04 | - |
| Unigene66182_S2A | 11.17 | 30.76 | 1.46 | 1.62E-13 | 6.77E-12 | Protein FAM3 |
| CL615.Contig2_S2A | 21.82 | 60.07 | 1.46 | 0 | 0 | Lysozyme 1a precursor |
| Unigene11123_S2A | 3.49 | 9.59 | 1.46 | 5.09E-05 | 6.31E-04 | - |
| Unigene11169_S2A | 6.46 | 17.73 | 1.46 | 3.26E-08 | 7.16E-07 | THAP domain-containing protein 3 |
| CL46.Contig1_S2A | 6.81 | 18.64 | 1.45 | 1.56E-08 | 3.62E-07 | Up-regulated during skeletal muscle growth protein 5-like |
| Unigene66652_S2A | 3.84 | 10.49 | 1.45 | 2.39E-05 | 3.18E-04 | - |
| Unigene65258_S2A | 5.06 | 13.75 | 1.44 | 1.41E-06 | 2.40E-05 | PCAF associated factor 65 beta-like |
| Unigene35889_S2A | 24.79 | 67.30 | 1.44 | 1.39E-13 | 5.87E-12 | Tetraspanin-13 |
| Unigene71902_S2A | 8.73 | 23.70 | 1.44 | 2.22E-10 | 6.45E-09 | Haptoglobin |
| CL4814.Contig1_S2A | 112.61 | 305.04 | 1.44 | 2.96E-13 | 1.20E-11 | - |
| Unigene71512_S2A | 32.47 | 87.39 | 1.43 | 4.29E-13 | 1.71E-11 | Interferon stimulated exonuclease-like |
| Unigene59041_S2A | 8.21 | 22.07 | 1.43 | 1.21E-09 | 3.21E-08 | 26S proteasome non-ATPase regulatory subunit 6 |
| Unigene26449_S2A | 6.11 | 16.28 | 1.41 | 2.25E-07 | 4.39E-06 | Clusterin preproprotein |
| Unigene25330_S2A | 6.81 | 18.09 | 1.41 | 5.09E-08 | 1.09E-06 | Translocator protein |
| Unigene72937_S2A | 5.59 | 14.84 | 1.41 | 8.37E-07 | 1.49E-05 | - |
| Unigene21033_S2A | 7.33 | 19.36 | 1.40 | 2.04E-08 | 4.65E-07 | - |
| Unigene26099_S2A | 20.78 | 54.82 | 1.40 | 0 | 0 | V-type proton ATPase subunit d 1 |
| Unigene4240_S2A | 8.03 | 21.17 | 1.40 | 4.64E-09 | 1.16E-07 | - |
| Unigene4327_S2A | 9.95 | 26.23 | 1.40 | 6.67E-11 | 2.04E-09 | Four and a half LIM domains protein 2 |
| Unigene26402_S2A | 7.51 | 19.72 | 1.39 | 1.71E-08 | 3.95E-07 | Dolichyl-diphosphooligosaccharide--protein glycosyltransferase subunit DAD1 |
| Unigene26151_S2A | 28.98 | 75.99 | 1.39 | 4.11E-14 | 1.84E-12 | - |
| Unigene65350_S2A | 9.43 | 24.61 | 1.38 | 3.58E-10 | 1.00E-08 | POU domain class 2-associating factor 1 |
| Unigene65238_S2A | 8.55 | 22.25 | 1.38 | 2.74E-09 | 7.01E-08 | - |
| Unigene26632_S2A | 37.36 | 96.98 | 1.38 | 0 | 0 | SEC31 homolog A |
| Unigene59408_S2A | 4.19 | 10.86 | 1.37 | 3.70E-05 | 4.75E-04 | Z-DNA binding protein 1-like |
| Unigene14130_S2A | 4.19 | 10.86 | 1.37 | 3.70E-05 | 4.74E-04 | Growth factor receptor-bound protein 10 |
| Unigene26853_S2A | 30.55 | 78.88 | 1.37 | 2.04E-13 | 8.35E-12 | Complement C3 |
| Unigene70593_S2A | 24.09 | 61.88 | 1.36 | 5.13E-14 | 2.27E-12 | Zinc transporter SLC39A7 precursor |
| Unigene11624_S2A | 56.39 | 144.56 | 1.36 | 0 | 0 | Probable C->U-editing enzyme APOBEC-2 |
| Unigene77451_S2A | 4.54 | 11.58 | 1.35 | 2.56E-05 | 3.38E-04 | CTD small phosphatase-like protein-like |
| Unigene26486_S2A | 16.06 | 40.71 | 1.34 | 0 | 0 | FBL protein |
| CL3300.Contig1_S2A | 7.68 | 19.36 | 1.33 | 6.58E-08 | 1.39E-06 | Serum amyloid A protein |
| Unigene72739_S2A | 18.51 | 46.32 | 1.32 | 0 | 0 | - |
| Unigene26071_S2A | 219.28 | 547.12 | 1.32 | 3.05E-12 | 1.09E-10 | - |
| CL3566.Contig1_S2A | 28.98 | 72.01 | 1.31 | 4.11E-14 | 1.84E-12 | Cysteine-rich protein 2 |
| Unigene59869_S2A | 5.41 | 13.39 | 1.31 | 1.01E-05 | 1.44E-04 | HCG2040801 |
| Unigene42576_S2A | 10.82 | 26.78 | 1.31 | 3.82E-10 | 1.06E-08 | - |
| Unigene77622_S2A | 18.33 | 45.23 | 1.30 | 0 | 0 | - |
| Unigene36016_S2A | 6.28 | 15.20 | 1.28 | 3.93E-06 | 6.08E-05 | WD repeat-containing protein 38-like |
| Unigene66643_S2A | 9.08 | 21.89 | 1.27 | 3.14E-08 | 6.92E-07 | - |
| CL4078.Contig1_S2A | 10.13 | 24.42 | 1.27 | 4.96E-09 | 1.23E-07 | Malignant T cell-amplified sequence 1 isoform 1 |
| Unigene25767_S2A | 26.89 | 64.77 | 1.27 | 4.15E-14 | 1.86E-12 | HSPC307 |
| Unigene26414_S2A | 38.23 | 91.19 | 1.25 | 1.67E-13 | 6.95E-12 | - |
| Unigene25278_S2A | 37.71 | 89.92 | 1.25 | 1.11E-13 | 4.76E-12 | Sequestosome-1 |
| Unigene26304_S2A | 4.89 | 11.58 | 1.24 | 7.84E-05 | 9.24E-04 | EMILIN-1 precursor |
| Unigene70875_S2A | 19.55 | 46.14 | 1.24 | 0 | 0 | - |
| Unigene65792_S2A | 8.03 | 18.82 | 1.23 | 5.87E-07 | 1.08E-05 | Chromobox-like protein 4 |
| Unigene73718_S2A | 8.38 | 19.54 | 1.22 | 4.01E-07 | 7.55E-06 | Mitochondrial fission process protein 1 |
| Unigene80102_S2A | 20.43 | 47.40 | 1.21 | 1.49E-13 | 6.28E-12 | TKL protein kinase |
| Unigene6329_S2A | 7.51 | 17.37 | 1.21 | 2.12E-06 | 3.47E-05 | - |
| Unigene11328_S2A | 15.01 | 34.56 | 1.20 | 2.56E-11 | 8.26E-10 | - |
| CL389.Contig1_S2A | 8.73 | 20.08 | 1.20 | 3.88E-07 | 7.35E-06 | WD repeat-containing protein 33 isoform 2 |
| Unigene66864_S2A | 9.78 | 22.25 | 1.19 | 1.23E-07 | 2.48E-06 | Ceruloplasmin (ferroxidase) isoform 1 |
| Unigene66508_S2A | 30.73 | 69.48 | 1.18 | 0 | 0 | - |
| Unigene10796_S2A | 5.94 | 13.39 | 1.17 | 4.95E-05 | 6.15E-04 | Canopy 2 homolog |
| Unigene10852_S2A | 25.14 | 56.63 | 1.17 | 0 | 0 | 26S protease regulatory subunit 6A |
| CL3660.Contig1_S2A | 12.22 | 27.50 | 1.17 | 5.96E-09 | 1.47E-07 | Guanine nucleotide-binding protein subunit beta-2-like 1-like isoform 4 |
| Unigene11468_S2A | 11.35 | 25.51 | 1.17 | 2.18E-08 | 4.95E-07 | - |
| Unigene73492_S2A | 10.82 | 24.24 | 1.16 | 5.46E-08 | 1.16E-06 | F-box only protein 38-like isoform 2 |
| Unigene11258_S2A | 70.53 | 156.32 | 1.15 | 2.16E-13 | 8.80E-12 | Dipeptidyl peptidase 1 |
| CL2947.Contig1_S2A | 10.47 | 23.16 | 1.15 | 1.58E-07 | 3.16E-06 | Natural killer cells antigen CD94-like |
| CL2755.Contig1_S2A | 11.87 | 26.23 | 1.14 | 2.41E-08 | 5.41E-07 | Chain A, Structure Of Cdk2CYCLIN A WITH PNU-292137 |
| Unigene25400_S2A | 9.95 | 21.89 | 1.14 | 3.95E-07 | 7.46E-06 | 26S proteasome complex subunit DSS1 |
| Unigene11868_S2A | 14.32 | 31.48 | 1.14 | 1.17E-09 | 3.11E-08 | ORM1-like protein 2 |
| Unigene26307_S2A | 8.73 | 19.18 | 1.14 | 2.16E-06 | 3.53E-05 | 60S ribosomal protein L27 |
| Unigene26213_S2A | 8.73 | 19.18 | 1.14 | 2.16E-06 | 3.53E-05 | Dolichyl-diphosphooligosaccharide--protein glycosyltransferase subunit STT3A |
| Unigene65924_S2A | 7.86 | 17.19 | 1.13 | 8.03E-06 | 1.16E-04 | - |
| Unigene10299_S2A | 6.46 | 14.11 | 1.13 | 5.44E-05 | 6.70E-04 | Endonuclease reverse transcriptase |
| Unigene26522_S2A | 8.38 | 18.27 | 1.12 | 4.46E-06 | 6.83E-05 | Lymphotoxin beta (TNF superfamily, member 3) |
| Unigene26012_S2A | 6.81 | 14.84 | 1.12 | 3.66E-05 | 4.71E-04 | ADP/ATP translocase 2 |
| Unigene71887_S2A | 69.83 | 151.62 | 1.12 | 0 | 0 | - |
| Unigene26726_S2A | 9.43 | 20.44 | 1.12 | 1.38E-06 | 2.35E-05 | Factor XIIa inhibitor precursor |
| Unigene80801_S2A | 8.73 | 18.82 | 1.11 | 4.20E-06 | 6.47E-05 | - |
| Unigene10525_S2A | 20.25 | 43.60 | 1.11 | 2.48E-12 | 8.91E-11 | - |
| Unigene26425_S2A | 9.78 | 20.99 | 1.10 | 1.30E-06 | 2.22E-05 | GG15699 |
| Unigene73041_S2A | 11.00 | 23.52 | 1.10 | 3.32E-07 | 6.34E-06 | - |
| CL628.Contig5_S2A | 20.43 | 43.60 | 1.09 | 3.91E-12 | 1.38E-10 | Tubulin alpha-1B chain-like |
| Unigene65439_S2A | 18.33 | 39.08 | 1.09 | 5.17E-11 | 1.61E-09 | Inositol(myo)-1(or 4)-monophosphatase 2-like |
| Unigene60391_S2A | 12.22 | 26.05 | 1.09 | 8.50E-08 | 1.77E-06 | - |
| Unigene26691_S2A | 7.16 | 15.20 | 1.09 | 4.79E-05 | 5.98E-04 | Mesencephalic astrocyte-derived neurotrophic factor precursor |
| Unigene21238_S2A | 23.39 | 49.57 | 1.08 | 3.29E-14 | 1.49E-12 | - |
| Unigene26585_S2A | 15.54 | 32.93 | 1.08 | 2.15E-09 | 5.56E-08 | - |
| Unigene70988_S2A | 20.08 | 42.52 | 1.08 | 1.04E-11 | 3.51E-10 | - |
| Unigene25553_S2A | 38.93 | 82.14 | 1.08 | 0 | 0 | Translocon-associated protein subunit delta-like |
| Unigene26480_S2A | 98.99 | 208.43 | 1.07 | 0 | 0 | Cytochrome c oxidase subunit 4 isoform 1, mitochondrial precursor |
| Unigene26190_S2A | 6.98 | 14.65 | 1.07 | 8.10E-05 | 9.49E-04 | T-complex protein 1 subunit gamma |
| Unigene41937_S2A | 9.43 | 19.72 | 1.06 | 5.09E-06 | 7.69E-05 | - |
| Unigene11299_S2A | 22.17 | 46.32 | 1.06 | 2.70E-12 | 9.66E-11 | Esophageal cancer related gene 4 protein precursor |
| Unigene10681_S2A | 14.32 | 29.85 | 1.06 | 2.16E-08 | 4.90E-07 | DnaJ homolog subfamily B member 11 precursor |
| Unigene25433_S2A | 168.82 | 351.90 | 1.06 | 0 | 0 | Regakine 1-like protein precursor |
| Unigene26290_S2A | 38.58 | 80.15 | 1.05 | 0 | 0 | Jun D proto-oncogene |
| Unigene35744_S2A | 20.60 | 42.70 | 1.05 | 2.83E-11 | 9.07E-10 | Ankyrin repeat domain-containing protein 39 |
| Unigene71897_S2A | 10.13 | 20.99 | 1.05 | 3.19E-06 | 5.04E-05 | Fibrosin-1 |
| CL4752.Contig2_S2A | 44.69 | 92.45 | 1.05 | 0 | 0 | Alpha-enolase |
| CL437.Contig1_S2A | 123.60 | 254.92 | 1.04 | 8.43E-13 | 3.24E-11 | - |
| Unigene25887_S2A | 196.93 | 406.00 | 1.04 | 4.72E-12 | 1.65E-10 | Vigilin-like |
| Unigene70489_S2A | 97.77 | 201.01 | 1.04 | 0 | 0 | Quiescin Q6 sulfhydryl oxidase 2 |
| Unigene70831_S2A | 8.03 | 16.46 | 1.04 | 4.67E-05 | 5.83E-04 | P450c17 |
| Unigene25549_S2A | 29.33 | 60.07 | 1.03 | 0 | 0 | Stabilin-2 precursor |
| Unigene26335_S2A | 18.68 | 38.18 | 1.03 | 5.97E-10 | 1.63E-08 | Collagen |
| CL2243.Contig1_S2A | 16.41 | 33.29 | 1.02 | 9.99E-09 | 2.39E-07 | Ornithine decarboxylase antizyme |
| Unigene71089_S2A | 8.03 | 16.28 | 1.02 | 6.39E-05 | 7.76E-04 | Cleavage and polyadenylation specificity factor subunit 5 |
| Unigene4615_S2A | 11.52 | 23.34 | 1.02 | 1.70E-06 | 2.83E-05 | - |
| Unigene16631_S2A | 20.95 | 42.34 | 1.02 | 1.22E-10 | 3.64E-09 | Protein BTG3 |
| Unigene26918_S2A | 16.93 | 34.01 | 1.01 | 1.03E-08 | 2.45E-07 | DDOST 48 kDa subunit |
| Unigene71266_S2A | 12.57 | 25.15 | 1.00 | 9.71E-07 | 1.71E-05 | - |
| Unigene35638_S2A | 9.95 | 19.90 | 1.00 | 1.35E-05 | 1.89E-04 | - |
| CL2371.Contig1_S2A | 89.04 | 0.01 | -13.12 | 2.51E-150 | 5.93E-148 | DQB*0101 beta chain precursor |
| Unigene57791_S2A | 22.70 | 0.01 | -11.15 | 7.45E-39 | 8.46E-37 | - |
| CL4566.Contig1_S2A | 15.54 | 0.01 | -10.60 | 7.93E-27 | 6.84E-25 | Glutaredoxin-3 |
| Unigene80748_S2A | 8.90 | 0.01 | -9.80 | 1.11E-15 | 5.71E-14 | - |
| Unigene5230_S2A | 8.38 | 0.01 | -9.71 | 8.45E-15 | 4.07E-13 | - |
| Unigene32758_S2A | 6.98 | 0.01 | -9.45 | 1.88E-12 | 6.98E-11 | - |
| CL4430.Contig2_S2A | 6.63 | 0.01 | -9.37 | 7.25E-12 | 2.49E-10 | - |
| Unigene3101_S2A | 6.46 | 0.01 | -9.34 | 1.42E-11 | 4.73E-10 | - |
| Unigene77105_S2A | 6.28 | 0.01 | -9.29 | 2.80E-11 | 8.99E-10 | Lebercilin-like protein |
| Unigene12147_S2A | 6.11 | 0.01 | -9.26 | 5.50E-11 | 1.70E-09 | UBE2O protein |
| CL4157.Contig1_S2A | 5.94 | 0.01 | -9.21 | 1.08E-10 | 3.24E-09 | - |
| CL2579.Contig2_S2A | 4.36 | 0.01 | -8.77 | 4.72E-08 | 1.02E-06 | - |
| Unigene73326_S2A | 4.19 | 0.01 | -8.71 | 9.27E-08 | 1.91E-06 | - |
| Unigene59784_S2A | 4.19 | 0.01 | -8.71 | 9.27E-08 | 1.91E-06 | Peptidyl-tRNA hydrolase domain-containing protein 1 |
| Unigene62034_S2A | 4.02 | 0.01 | -8.65 | 1.82E-07 | 3.61E-06 | Double C2-like domain-containing protein gamma |
| Unigene22737_S2A | 4.02 | 0.01 | -8.65 | 1.82E-07 | 3.61E-06 | - |
| Unigene24169_S2A | 3.67 | 0.01 | -8.52 | 7.04E-07 | 1.27E-05 | Non-SMC condensin I complex, subunit H |
| Unigene210_S2A | 3.67 | 0.01 | -8.52 | 7.04E-07 | 1.27E-05 | Multidrug resistance-associated protein 4-like |
| Unigene73306_S2A | 3.67 | 0.01 | -8.52 | 7.04E-07 | 1.27E-05 | - |
| CL927.Contig1_S2A | 3.67 | 0.01 | -8.52 | 7.04E-07 | 1.27E-05 | Interferon alpha-inducible protein 27 |
| Unigene16619_S2A | 3.67 | 0.01 | -8.52 | 7.04E-07 | 1.27E-05 | - |
| Unigene23626_S2A | 3.32 | 0.01 | -8.38 | 2.72E-06 | 4.35E-05 | - |
| CL2579.Contig1_S2A | 3.14 | 0.01 | -8.29 | 5.34E-06 | 8.05E-05 | - |
| Unigene22271_S2A | 3.14 | 0.01 | -8.29 | 5.34E-06 | 8.04E-05 | - |
| Unigene78093_S2A | 3.14 | 0.01 | -8.29 | 5.34E-06 | 8.04E-05 | - |
| CL4231.Contig2_S2A | 3.14 | 0.01 | -8.29 | 5.34E-06 | 8.03E-05 | SLAM family member 9-like |
| Unigene72582_S2A | 3.14 | 0.01 | -8.29 | 5.34E-06 | 8.03E-05 | - |
| Unigene62284_S2A | 3.14 | 0.01 | -8.29 | 5.34E-06 | 8.02E-05 | - |
| Unigene36218_S2A | 2.97 | 0.01 | -8.21 | 1.05E-05 | 1.50E-04 | - |
| Unigene64380_S2A | 2.97 | 0.01 | -8.21 | 1.05E-05 | 1.50E-04 | Endonuclease reverse transcriptase |
| Unigene25042_S2A | 2.97 | 0.01 | -8.21 | 1.05E-05 | 1.49E-04 | - |
| Unigene71484_S2A | 2.97 | 0.01 | -8.21 | 1.05E-05 | 1.49E-04 | Epidermal growth factor receptor substrate 15-like 1 |
| Unigene76245_S2A | 2.97 | 0.01 | -8.21 | 1.05E-05 | 1.49E-04 | - |
| Unigene76352_S2A | 2.97 | 0.01 | -8.21 | 1.05E-05 | 1.49E-04 | - |
| Unigene36611_S2A | 2.97 | 0.01 | -8.21 | 1.05E-05 | 1.49E-04 | - |
| Unigene35993_S2A | 2.97 | 0.01 | -8.21 | 1.05E-05 | 1.49E-04 | - |
| Unigene916_S2A | 2.97 | 0.01 | -8.21 | 1.05E-05 | 1.49E-04 | RPGR protein |
| Unigene38649_S2A | 2.79 | 0.01 | -8.12 | 2.06E-05 | 2.79E-04 | - |
| Unigene17634_S2A | 2.79 | 0.01 | -8.12 | 2.06E-05 | 2.79E-04 | - |
| Unigene7131_S2A | 2.79 | 0.01 | -8.12 | 2.06E-05 | 2.79E-04 | - |
| Unigene40879_S2A | 2.79 | 0.01 | -8.12 | 2.06E-05 | 2.79E-04 | - |
| Unigene70956_S2A | 2.79 | 0.01 | -8.12 | 2.06E-05 | 2.79E-04 | - |
| Unigene43071_S2A | 2.62 | 0.01 | -8.03 | 4.05E-05 | 5.15E-04 | - |
| Unigene80786_S2A | 2.62 | 0.01 | -8.03 | 4.05E-05 | 5.14E-04 | - |
| Unigene24473_S2A | 2.62 | 0.01 | -8.03 | 4.05E-05 | 5.14E-04 | - |
| Unigene72811_S2A | 2.62 | 0.01 | -8.03 | 4.05E-05 | 5.14E-04 | Isthmin-1 |
| Unigene73294_S2A | 2.62 | 0.01 | -8.03 | 4.05E-05 | 5.14E-04 | - |
| CL4536.Contig2_S2A | 2.62 | 0.01 | -8.03 | 4.05E-05 | 5.13E-04 | G-protein coupled receptor 183 |
| Unigene38367_S2A | 2.62 | 0.01 | -8.03 | 4.05E-05 | 5.13E-04 | - |
| Unigene15767_S2A | 2.62 | 0.01 | -8.03 | 4.05E-05 | 5.13E-04 | Xanthine dehydrogenase |
| Unigene56339_S2A | 2.44 | 0.01 | -7.93 | 7.96E-05 | 9.37E-04 | - |
| Unigene22902_S2A | 2.44 | 0.01 | -7.93 | 7.96E-05 | 9.36E-04 | - |
| Unigene66357_S2A | 2.44 | 0.01 | -7.93 | 7.96E-05 | 9.36E-04 | - |
| Unigene58065_S2A | 2.44 | 0.01 | -7.93 | 7.96E-05 | 9.35E-04 | - |
| Unigene26362_S2A | 2.44 | 0.01 | -7.93 | 7.96E-05 | 9.35E-04 | Reverse transcriptase-like |
| Unigene3740_S2A | 60.93 | 0.36 | -7.40 | 6.37E-99 | 1.25E-96 | - |
| CL1369.Contig1_S2A | 26.01 | 0.54 | -5.59 | 1.41E-39 | 1.61E-37 | - |
| Unigene37819_S2A | 42.60 | 1.81 | -4.56 | 6.14E-58 | 9.49E-56 | - |
| Unigene77839_S2A | 8.21 | 0.36 | -4.51 | 5.12E-12 | 1.78E-10 | - |
| Unigene60385_S2A | 7.16 | 0.36 | -4.31 | 2.29E-10 | 6.61E-09 | - |
| Unigene67645_S2A | 6.98 | 0.36 | -4.28 | 4.30E-10 | 1.19E-08 | - |
| Unigene3826_S2A | 17.28 | 0.90 | -4.26 | 2.69E-23 | 2.05E-21 | - |
| Unigene36643_S2A | 13.27 | 0.72 | -4.20 | 5.27E-18 | 3.15E-16 | - |
| Unigene35694_S2A | 16.24 | 0.90 | -4.17 | 1.15E-21 | 8.36E-20 | Nitric oxide synthase, endothelial |
| Unigene4918_S2A | 12.74 | 0.72 | -4.15 | 3.44E-17 | 1.95E-15 | Endonuclease reverse transcriptase |
| Unigene30751_S2A | 9.08 | 0.54 | -4.07 | 1.97E-12 | 7.29E-11 | - |
| Unigene65904_S2A | 9.08 | 0.54 | -4.07 | 1.97E-12 | 7.28E-11 | Testis-expressed sequence 264 protein |
| Unigene36203_S2A | 8.21 | 0.54 | -3.93 | 4.37E-11 | 1.37E-09 | Uncharacterized protein LOC614600 precursor |
| Unigene11733_S2A | 16.41 | 1.09 | -3.91 | 5.14E-21 | 3.62E-19 | - |
| Unigene44450_S2A | 7.68 | 0.54 | -3.83 | 2.76E-10 | 7.91E-09 | - |
| Unigene42171_S2A | 4.89 | 0.36 | -3.76 | 7.47E-07 | 1.34E-05 | B4GALNT1 protein |
| Unigene2048_S2A | 4.71 | 0.36 | -3.71 | 1.38E-06 | 2.35E-05 | - |
| Unigene4066_S2A | 16.59 | 1.27 | -3.71 | 2.03E-20 | 1.37E-18 | - |
| CL3078.Contig1_S2A | 18.68 | 1.45 | -3.69 | 9.67E-23 | 7.32E-21 | Centrosomal protein 110kDa |
| Unigene36277_S2A | 4.54 | 0.36 | -3.66 | 2.53E-06 | 4.08E-05 | - |
| Unigene43460_S2A | 11.17 | 0.90 | -3.63 | 6.41E-14 | 2.81E-12 | - |
| Unigene71044_S2A | 11.00 | 0.90 | -3.61 | 1.17E-13 | 4.99E-12 | Cytosolic Fe-S cluster assembly factor NUBP1 |
| Unigene72401_S2A | 4.36 | 0.36 | -3.60 | 4.64E-06 | 7.09E-05 | - |
| Unigene72536_S2A | 32.12 | 2.71 | -3.57 | 3.99E-37 | 4.40E-35 | - |
| Unigene7186_S2A | 4.19 | 0.36 | -3.54 | 8.50E-06 | 1.23E-04 | - |
| Unigene60351_S2A | 10.30 | 0.90 | -3.52 | 1.29E-12 | 4.85E-11 | - |
| Unigene38152_S2A | 4.02 | 0.36 | -3.48 | 1.55E-05 | 2.15E-04 | - |
| Unigene71238_S2A | 62.15 | 5.79 | -3.42 | 4.20E-68 | 7.02E-66 | - |
| Unigene39140_S2A | 5.76 | 0.54 | -3.42 | 2.14E-07 | 4.19E-06 | - |
| Unigene38224_S2A | 3.84 | 0.36 | -3.42 | 2.82E-05 | 3.69E-04 | - |
| Unigene12155_S2A | 3.84 | 0.36 | -3.42 | 2.82E-05 | 3.69E-04 | - |
| CL2751.Contig1_S2A | 3.67 | 0.36 | -3.35 | 5.12E-05 | 6.35E-04 | - |
| Unigene80107_S2A | 3.67 | 0.36 | -3.35 | 5.12E-05 | 6.34E-04 | Ubiquitin carboxyl-terminal hydrolase 8 |
| Unigene66090_S2A | 5.41 | 0.54 | -3.32 | 6.99E-07 | 1.27E-05 | Regulator of G-protein signaling 2 |
| Unigene51171_S2A | 10.65 | 1.09 | -3.29 | 2.21E-12 | 8.09E-11 | - |
| Unigene1335_S2A | 5.24 | 0.54 | -3.28 | 1.26E-06 | 2.16E-05 | - |
| CL3288.Contig1_S2A | 10.47 | 1.09 | -3.26 | 3.96E-12 | 1.39E-10 | - |
| Unigene16499_S2A | 69.31 | 7.24 | -3.26 | 1.55E-72 | 2.78E-70 | - |
| Unigene37561_S2A | 19.03 | 1.99 | -3.26 | 6.13E-21 | 4.28E-19 | - |
| Unigene25985_S2A | 339.56 | 36.73 | -3.21 | 0 | 0 | - |
| Unigene4514_S2A | 21.65 | 2.35 | -3.20 | 2.73E-23 | 2.08E-21 | ATP-binding cassette, sub-family C, member 4 |
| CL1299.Contig1_S2A | 6.63 | 0.72 | -3.20 | 5.79E-08 | 1.23E-06 | MHC class II DO-beta chain |
| Unigene71189_S2A | 41.38 | 4.52 | -3.19 | 4.06E-43 | 5.08E-41 | Tenascin C |
| Unigene14164_S2A | 27.93 | 3.08 | -3.18 | 1.72E-29 | 1.60E-27 | - |
| Unigene45412_S2A | 4.89 | 0.54 | -3.18 | 4.06E-06 | 6.28E-05 | - |
| Unigene70668_S2A | 173.19 | 19.18 | -3.17 | 2.14E-174 | 5.18E-172 | - |
| Unigene14106_S2A | 9.78 | 1.09 | -3.17 | 4.05E-11 | 1.27E-09 | - |
| Unigene11706_S2A | 17.63 | 1.99 | -3.15 | 6.26E-19 | 3.95E-17 | - |
| Unigene55415_S2A | 4.71 | 0.54 | -3.12 | 7.25E-06 | 1.06E-04 | - |
| Unigene80305_S2A | 4.71 | 0.54 | -3.12 | 7.25E-06 | 1.06E-04 | Mariner transposase |
| Unigene53554_S2A | 5.94 | 0.72 | -3.04 | 5.82E-07 | 1.07E-05 | - |
| Unigene16778_S2A | 5.76 | 0.72 | -3.00 | 1.03E-06 | 1.80E-05 | - |
| Unigene72808_S2A | 5.76 | 0.72 | -3.00 | 1.03E-06 | 1.80E-05 | - |
| Unigene25311_S2A | 41.38 | 5.25 | -2.98 | 2.10E-40 | 2.43E-38 | - |
| Unigene80373_S2A | 5.59 | 0.72 | -2.96 | 1.82E-06 | 3.01E-05 | - |
| Unigene59527_S2A | 9.78 | 1.27 | -2.95 | 1.85E-10 | 5.46E-09 | - |
| Unigene60384_S2A | 9.78 | 1.27 | -2.95 | 1.85E-10 | 5.45E-09 | - |
| Unigene35443_S2A | 9.60 | 1.27 | -2.92 | 3.25E-10 | 9.18E-09 | Salt-inducible kinase 1-like |
| Unigene51418_S2A | 5.41 | 0.72 | -2.91 | 3.20E-06 | 5.05E-05 | - |
| Unigene13259_S2A | 4.02 | 0.54 | -2.90 | 7.16E-05 | 8.52E-04 | - |
| Unigene29702_S2A | 4.02 | 0.54 | -2.90 | 7.16E-05 | 8.52E-04 | - |
| Unigene60299_S2A | 4.02 | 0.54 | -2.90 | 7.16E-05 | 8.52E-04 | - |
| Unigene26093_S2A | 4.02 | 0.54 | -2.90 | 7.16E-05 | 8.51E-04 | IKAROS family zinc finger 2 (Helios) |
| Unigene46101_S2A | 10.65 | 1.45 | -2.88 | 4.76E-11 | 1.48E-09 | - |
| Unigene42200_S2A | 13.27 | 1.81 | -2.87 | 1.94E-13 | 8.01E-12 | - |
| Unigene49504_S2A | 5.24 | 0.72 | -2.86 | 5.61E-06 | 8.39E-05 | - |
| Unigene42554_S2A | 6.46 | 0.90 | -2.84 | 4.50E-07 | 8.41E-06 | - |
| Unigene70540_S2A | 17.81 | 2.53 | -2.82 | 3.08E-17 | 1.76E-15 | Hypoxia-inducible factor prolyl 4-hydroxylase |
| Unigene11644_S2A | 6.28 | 0.90 | -2.80 | 7.84E-07 | 1.40E-05 | Aspartate aminotransferase, mitochondrial |
| CL3273.Contig1_S2A | 26.01 | 3.80 | -2.77 | 3.22E-24 | 2.52E-22 | Sentrin-specific protease 6 |
| Unigene59036_S2A | 27.23 | 3.98 | -2.77 | 2.79E-25 | 2.25E-23 | - |
| Unigene71552_S2A | 65.47 | 9.59 | -2.77 | 1.36E-58 | 2.12E-56 | - |
| Unigene75694_S2A | 4.89 | 0.72 | -2.76 | 1.71E-05 | 2.35E-04 | - |
| Unigene51348_S2A | 12.22 | 1.81 | -2.76 | 5.30E-12 | 1.84E-10 | - |
| Unigene629_S2A | 16.76 | 2.53 | -2.73 | 8.19E-16 | 4.25E-14 | - |
| Unigene4568_S2A | 15.54 | 2.35 | -2.73 | 9.61E-15 | 4.59E-13 | UDP-N-acteylglucosamine pyrophosphorylase 1-like 1 |
| Unigene9982_S2A | 8.38 | 1.27 | -2.72 | 1.57E-08 | 3.65E-07 | - |
| Unigene21469_S2A | 16.59 | 2.53 | -2.71 | 1.41E-15 | 7.18E-14 | - |
| Unigene72778_S2A | 10.65 | 1.63 | -2.71 | 1.88E-10 | 5.51E-09 | Interferon-induced very large GTPase 1, partial |
| Unigene24237_S2A | 44.87 | 6.88 | -2.71 | 1.16E-39 | 1.34E-37 | - |
| Unigene5732_S2A | 14.14 | 2.17 | -2.70 | 1.94E-13 | 8.01E-12 | - |
| CL2059.Contig1_S2A | 12.92 | 1.99 | -2.70 | 2.29E-12 | 8.34E-11 | - |
| Unigene80008_S2A | 12.92 | 1.99 | -2.70 | 2.29E-12 | 8.32E-11 | - |
| Unigene20837_S2A | 12.92 | 1.99 | -2.70 | 2.29E-12 | 8.31E-11 | - |
| Unigene72738_S2A | 12.74 | 1.99 | -2.68 | 3.94E-12 | 1.38E-10 | - |
| Unigene14353_S2A | 4.54 | 0.72 | -2.66 | 5.12E-05 | 6.35E-04 | - |
| Unigene60731_S2A | 4.54 | 0.72 | -2.66 | 5.12E-05 | 6.35E-04 | - |
| Unigene39195_S2A | 16.93 | 2.71 | -2.64 | 1.78E-15 | 8.99E-14 | - |
| Unigene47172_S2A | 5.59 | 0.90 | -2.63 | 7.01E-06 | 1.03E-04 | - |
| Unigene76634_S2A | 5.59 | 0.90 | -2.63 | 7.01E-06 | 1.03E-04 | - |
| Unigene67675_S2A | 7.86 | 1.27 | -2.63 | 8.01E-08 | 1.68E-06 | - |
| CL215.Contig1_S2A | 7.86 | 1.27 | -2.63 | 8.01E-08 | 1.67E-06 | Sentrin specific peptidase 6 |
| Unigene65141_S2A | 6.63 | 1.09 | -2.60 | 9.75E-07 | 1.71E-05 | - |
| Unigene21198_S2A | 5.41 | 0.90 | -2.59 | 1.20E-05 | 1.70E-04 | KIAA1086 protein-like |
| Unigene25740_S2A | 5.41 | 0.90 | -2.59 | 1.20E-05 | 1.70E-04 | EH domain-binding protein 1-like protein 1 |
| CL1573.Contig3_S2A | 10.82 | 1.81 | -2.58 | 3.98E-10 | 1.10E-08 | Lymphoid-restricted membrane protein-like |
| Unigene60837_S2A | 6.46 | 1.09 | -2.57 | 1.67E-06 | 2.78E-05 | - |
| Unigene74031_S2A | 8.55 | 1.45 | -2.56 | 3.31E-08 | 7.27E-07 | Protein Wnt-11-like |
| Unigene11623_S2A | 10.65 | 1.81 | -2.56 | 6.77E-10 | 1.84E-08 | - |
| Unigene77568_S2A | 5.24 | 0.90 | -2.54 | 2.05E-05 | 2.79E-04 | 5-azacytidine induced 1 isoform 1 |
| Unigene36007_S2A | 5.24 | 0.90 | -2.54 | 2.05E-05 | 2.79E-04 | - |
| Unigene60447_S2A | 24.79 | 4.34 | -2.51 | 6.60E-21 | 4.56E-19 | - |
| Unigene9524_S2A | 11.35 | 1.99 | -2.51 | 2.80E-10 | 7.99E-09 | - |
| Unigene42031_S2A | 10.30 | 1.81 | -2.51 | 1.94E-09 | 5.05E-08 | - |
| Unigene25996_S2A | 18.33 | 3.26 | -2.49 | 1.10E-15 | 5.65E-14 | - |
| Unigene41462_S2A | 6.11 | 1.09 | -2.49 | 4.83E-06 | 7.34E-05 | - |
| Unigene20995_S2A | 6.11 | 1.09 | -2.49 | 4.83E-06 | 7.34E-05 | - |
| Unigene78427_S2A | 6.11 | 1.09 | -2.49 | 4.83E-06 | 7.33E-05 | - |
| Unigene80904_S2A | 14.14 | 2.53 | -2.48 | 2.42E-12 | 8.70E-11 | - |
| Unigene14551_S2A | 37.01 | 6.69 | -2.47 | 6.96E-30 | 6.65E-28 | - |
| Unigene46873_S2A | 17.98 | 3.26 | -2.46 | 3.11E-15 | 1.55E-13 | - |
| Unigene26239_S2A | 55.87 | 10.13 | -2.46 | 3.03E-44 | 3.87E-42 | - |
| Unigene70636_S2A | 15.89 | 2.89 | -2.46 | 1.45E-13 | 6.12E-12 | Fatty acid-binding protein |
| Unigene13469_S2A | 5.94 | 1.09 | -2.45 | 8.18E-06 | 1.18E-04 | - |
| Unigene64290_S2A | 5.94 | 1.09 | -2.45 | 8.18E-06 | 1.18E-04 | - |
| Unigene68969_S2A | 7.68 | 1.45 | -2.41 | 4.58E-07 | 8.55E-06 | - |
| CL2026.Contig1_S2A | 360.51 | 68.21 | -2.40 | 3.99E-268 | 1.06E-265 | HERV-K(C19) Pol protein |
| Unigene10431_S2A | 74.02 | 14.11 | -2.39 | 3.62E-56 | 5.41E-54 | - |
| Unigene49286_S2A | 6.63 | 1.27 | -2.38 | 3.24E-06 | 5.11E-05 | G-protein coupled receptor-associated sorting protein 1 |
| Unigene38869_S2A | 9.43 | 1.81 | -2.38 | 2.62E-08 | 5.83E-07 | - |
| Unigene71451_S2A | 21.47 | 4.16 | -2.37 | 3.63E-17 | 2.05E-15 | - |
| Unigene37177_S2A | 11.17 | 2.17 | -2.36 | 1.52E-09 | 3.97E-08 | - |
| CL2769.Contig1_S2A | 5.59 | 1.09 | -2.36 | 2.32E-05 | 3.10E-04 | N-acetylglucosamine-6-sulfatase precursor |
| Unigene7767_S2A | 10.13 | 1.99 | -2.35 | 1.05E-08 | 2.49E-07 | Protein SPHAR-like |
| Unigene5523_S2A | 13.79 | 2.71 | -2.35 | 2.16E-11 | 7.02E-10 | - |
| Unigene30224_S2A | 5.41 | 1.09 | -2.31 | 3.88E-05 | 4.95E-04 | - |
| Unigene49301_S2A | 5.41 | 1.09 | -2.31 | 3.88E-05 | 4.94E-04 | - |
| Unigene4321_S2A | 5.41 | 1.09 | -2.31 | 3.88E-05 | 4.94E-04 | - |
| Unigene26948_S2A | 7.16 | 1.45 | -2.30 | 2.13E-06 | 3.50E-05 | Regulator of G-protein signaling 3-like isoform 3 |
| Unigene66939_S2A | 8.03 | 1.63 | -2.30 | 5.06E-07 | 9.39E-06 | Hydrogen exchanger 7-like |
| Unigene37964_S2A | 80.48 | 16.46 | -2.29 | 6.55E-58 | 1.01E-55 | - |
| Unigene42853_S2A | 22.00 | 4.52 | -2.28 | 7.40E-17 | 4.09E-15 | - |
| Unigene17859_S2A | 13.09 | 2.71 | -2.27 | 1.62E-10 | 4.78E-09 | Reverse transcriptase |
| Unigene31306_S2A | 6.11 | 1.27 | -2.27 | 1.51E-05 | 2.09E-04 | - |
| CL2723.Contig2_S2A | 5.24 | 1.09 | -2.27 | 6.46E-05 | 7.81E-04 | DENN domain-containing protein 1C |
| Unigene23364_S2A | 5.24 | 1.09 | -2.27 | 6.46E-05 | 7.80E-04 | - |
| Unigene16607_S2A | 5.24 | 1.09 | -2.27 | 6.46E-05 | 7.80E-04 | Nucleoporin like 1 |
| Unigene66512_S2A | 12.92 | 2.71 | -2.25 | 2.66E-10 | 7.65E-09 | - |
| Unigene73657_S2A | 24.97 | 5.25 | -2.25 | 1.20E-18 | 7.44E-17 | - |
| Unigene66856_S2A | 75.25 | 15.92 | -2.24 | 6.98E-53 | 1.02E-50 | EGF-containing fibulin-like extracellular matrix protein 1 precursor |
| CL1300.Contig1_S2A | 8.55 | 1.81 | -2.24 | 3.30E-07 | 6.32E-06 | Amino-terminal enhancer of split |
| Unigene3959_S2A | 7.68 | 1.63 | -2.24 | 1.38E-06 | 2.35E-05 | - |
| Unigene65936_S2A | 35.61 | 7.60 | -2.23 | 1.08E-25 | 9.04E-24 | - |
| Unigene59277_S2A | 22.00 | 4.70 | -2.23 | 2.16E-16 | 1.16E-14 | - |
| CL2101.Contig2_S2A | 39.63 | 8.50 | -2.22 | 2.68E-28 | 2.43E-26 | - |
| Unigene71107_S2A | 6.63 | 1.45 | -2.19 | 9.64E-06 | 1.38E-04 | Glycogen phosphorylase, muscle form |
| Unigene71937_S2A | 14.84 | 3.26 | -2.19 | 2.75E-11 | 8.83E-10 | Sialic acid binding Ig-like lectin 1, sialoadhesin |
| Unigene80066_S2A | 5.76 | 1.27 | -2.18 | 4.11E-05 | 5.19E-04 | - |
| Unigene66426_S2A | 5.76 | 1.27 | -2.18 | 4.11E-05 | 5.19E-04 | - |
| Unigene71411_S2A | 10.65 | 2.35 | -2.18 | 2.01E-08 | 4.59E-07 | - |
| Unigene81703_S2A | 211.94 | 46.86 | -2.18 | 9.85E-141 | 2.28E-138 | - |
| Unigene71619_S2A | 15.54 | 3.44 | -2.18 | 1.09E-11 | 3.68E-10 | - |
| Unigene70488_S2A | 56.22 | 12.48 | -2.17 | 1.92E-38 | 2.16E-36 | Adipose most abundant gene transcript 2 protein |
| Unigene81007_S2A | 647.00 | 144.20 | -2.17 | 0 | 0 | Unnamed protein product |
| Unigene4078_S2A | 8.03 | 1.81 | -2.15 | 1.45E-06 | 2.45E-05 | Sarcoglycan, alpha (50kDa dystrophin-associated glycoprotein) |
| Unigene80455_S2A | 8.03 | 1.81 | -2.15 | 1.45E-06 | 2.45E-05 | - |
| Unigene15997_S2A | 24.09 | 5.43 | -2.15 | 3.90E-17 | 2.20E-15 | - |
| Unigene30076_S2A | 5.59 | 1.27 | -2.14 | 6.74E-05 | 8.10E-04 | - |
| Unigene62078_S2A | 5.59 | 1.27 | -2.14 | 6.74E-05 | 8.10E-04 | - |
| CL1135.Contig1_S2A | 5.59 | 1.27 | -2.14 | 6.74E-05 | 8.09E-04 | Inositol 1,4,5-trisphosphate receptor type 2 isoform 3 |
| Unigene80044_S2A | 5.59 | 1.27 | -2.14 | 6.74E-05 | 8.09E-04 | - |
| Unigene92_S2A | 8.73 | 1.99 | -2.13 | 5.66E-07 | 1.04E-05 | - |
| Unigene66912_S2A | 24.44 | 5.61 | -2.12 | 4.08E-17 | 2.29E-15 | Autophagy-related protein 16-2 |
| Unigene72602_S2A | 10.13 | 2.35 | -2.11 | 8.64E-08 | 1.80E-06 | - |
| Unigene77416_S2A | 8.55 | 1.99 | -2.10 | 9.20E-07 | 1.63E-05 | - |
| CL3945.Contig1_S2A | 12.40 | 2.89 | -2.10 | 3.22E-09 | 8.16E-08 | - |
| Unigene4097_S2A | 6.98 | 1.63 | -2.10 | 9.95E-06 | 1.42E-04 | - |
| Unigene70529_S2A | 9.25 | 2.17 | -2.09 | 3.58E-07 | 6.82E-06 | - |
| Unigene65709_S2A | 9.25 | 2.17 | -2.09 | 3.58E-07 | 6.81E-06 | - |
| Unigene58997_S2A | 38.93 | 9.23 | -2.08 | 1.05E-25 | 8.80E-24 | - |
| CL2200.Contig2_S2A | 6.11 | 1.45 | -2.08 | 4.20E-05 | 5.31E-04 | Granzyme B (granzyme 2, cytotoxic T-lymphocyte-associated serine esterase 1)-like |
| Unigene51272_S2A | 14.49 | 3.44 | -2.07 | 1.97E-10 | 5.73E-09 | Fc receptor-like protein 1-like |
| Unigene36555_S2A | 16.76 | 3.98 | -2.07 | 7.53E-12 | 2.59E-10 | - |
| CL1963.Contig1_S2A | 8.38 | 1.99 | -2.07 | 1.49E-06 | 2.50E-05 | Pformin-like 1 |
| Unigene36015_S2A | 8.38 | 1.99 | -2.07 | 1.49E-06 | 2.50E-05 | Glyoxylate reductase/hydroxypyruvate reductase |
| CL4838.Contig1_S2A | 9.78 | 2.35 | -2.06 | 2.25E-07 | 4.40E-06 | TKT protein |
| Unigene4777_S2A | 12.74 | 3.08 | -2.05 | 3.29E-09 | 8.31E-08 | - |
| Unigene36679_S2A | 18.68 | 4.52 | -2.05 | 7.47E-13 | 2.90E-11 | - |
| Unigene25827_S2A | 8.21 | 1.99 | -2.04 | 2.40E-06 | 3.88E-05 | - |
| Unigene35555_S2A | 20.08 | 4.88 | -2.04 | 1.17E-13 | 4.98E-12 | Trinucleotide repeat containing 18 |
| Unigene39129_S2A | 5.94 | 1.45 | -2.03 | 6.81E-05 | 8.16E-04 | - |
| Unigene3635_S2A | 5.94 | 1.45 | -2.03 | 6.81E-05 | 8.15E-04 | - |
| CL151.Contig1_S2A | 15.54 | 3.80 | -2.03 | 7.87E-11 | 2.40E-09 | - |
| Unigene9257_S2A | 73.15 | 17.91 | -2.03 | 1.34E-45 | 1.75E-43 | - |
| Unigene26215_S2A | 945.02 | 231.76 | -2.03 | 0 | 0 | T-cell receptor delta chain J-C region - sheep |
| Unigene25810_S2A | 432.27 | 106.38 | -2.02 | 1.64E-259 | 4.25E-257 | RCG19955,isoform CRA_a |
| Unigene65379_S2A | 22.00 | 5.43 | -2.02 | 1.16E-14 | 5.52E-13 | - |
| Unigene386_S2A | 7.33 | 1.81 | -2.02 | 1.00E-05 | 1.43E-04 | MYB-binding protein 1A-like isoform 1 |
| Unigene44358_S2A | 14.66 | 3.62 | -2.02 | 3.20E-10 | 9.05E-09 | - |
| CL3737.Contig1_S2A | 11.70 | 2.89 | -2.02 | 2.15E-08 | 4.89E-07 | Protein rogdi homolog |
| CL4910.Contig1_S2A | 340.44 | 84.13 | -2.02 | 5.03E-204 | 1.24E-201 | Interferon-induced transmembrane protein 1 (9-27) isoform 1 |
| Unigene19437_S2A | 8.03 | 1.99 | -2.01 | 3.86E-06 | 6.00E-05 | - |
| Unigene59749_S2A | 9.43 | 2.35 | -2.00 | 5.81E-07 | 1.07E-05 | - |
| CL4689.Contig1_S2A | 15.71 | 3.98 | -1.98 | 1.26E-10 | 3.75E-09 | Protein FAM92A1 |
| Unigene65987_S2A | 8.55 | 2.17 | -1.98 | 2.39E-06 | 3.87E-05 | - |
| Unigene46990_S2A | 8.55 | 2.17 | -1.98 | 2.39E-06 | 3.87E-05 | ADP-ribosylation factor-like protein 6-interacting protein 6 |
| Unigene66597_S2A | 9.25 | 2.35 | -1.98 | 9.28E-07 | 1.64E-05 | IKAROS family zinc finger 2 |
| Unigene42555_S2A | 9.25 | 2.35 | -1.98 | 9.28E-07 | 1.64E-05 | - |
| Unigene71523_S2A | 11.35 | 2.89 | -1.97 | 5.48E-08 | 1.17E-06 | HCG2004862, isoform CRA_b |
| Unigene26871_S2A | 55.34 | 14.11 | -1.97 | 1.22E-33 | 1.25E-31 | NADH dehydrogenase [ubiquinone] 1 beta subcomplex subunit 10 [Bos taurus] |
| Unigene30028_S2A | 9.08 | 2.35 | -1.95 | 1.48E-06 | 2.48E-05 | - |
| Unigene64305_S2A | 17.46 | 4.52 | -1.95 | 1.93E-11 | 6.32E-10 | - |
| Unigene76886_S2A | 8.38 | 2.17 | -1.95 | 3.82E-06 | 5.93E-05 | - |
| Unigene26598_S2A | 7.68 | 1.99 | -1.95 | 9.88E-06 | 1.42E-04 | - |
| Unigene17160_S2A | 14.66 | 3.80 | -1.95 | 8.05E-10 | 2.17E-08 | - |
| Unigene39573_S2A | 11.87 | 3.08 | -1.95 | 3.40E-08 | 7.46E-07 | - |
| Unigene15716_S2A | 6.28 | 1.63 | -1.95 | 6.70E-05 | 8.07E-04 | - |
| CL3650.Contig1_S2A | 59.18 | 15.38 | -1.94 | 3.05E-35 | 3.21E-33 | - |
| Unigene73726_S2A | 24.97 | 6.51 | -1.94 | 1.14E-15 | 5.82E-14 | - |
| Unigene81221_S2A | 11.70 | 3.08 | -1.93 | 5.39E-08 | 1.15E-06 | - |
| Unigene58948_S2A | 17.81 | 4.70 | -1.92 | 1.90E-11 | 6.23E-10 | Four and a half LIM domains protein 3 |
| Unigene66868_S2A | 8.21 | 2.17 | -1.92 | 6.06E-06 | 8.99E-05 | RCG61429 |
| Unigene71104_S2A | 53.95 | 14.29 | -1.92 | 1.13E-31 | 1.13E-29 | Inactive serine protease PAMR1 precursor |
| CL5323.Contig1_S2A | 7.51 | 1.99 | -1.92 | 1.57E-05 | 2.17E-04 | Zinc finger protein 33B |
| CL2101.Contig1_S2A | 19.73 | 5.25 | -1.91 | 1.84E-12 | 6.86E-11 | - |
| Unigene26080_S2A | 101.08 | 26.96 | -1.91 | 1.49E-57 | 2.27E-55 | - |
| Unigene59652_S2A | 10.82 | 2.89 | -1.90 | 2.18E-07 | 4.27E-06 | - |
| CL3467.Contig1_S2A | 10.13 | 2.71 | -1.90 | 5.60E-07 | 1.03E-05 | Interferon-tau-like |
| Unigene26572_S2A | 500.70 | 134.61 | -1.90 | 1.40E-275 | 3.83E-273 | Lymphocyte antigen 6E precursor |
| Unigene11762_S2A | 8.73 | 2.35 | -1.89 | 3.71E-06 | 5.79E-05 | - |
| Unigene65861_S2A | 8.73 | 2.35 | -1.89 | 3.71E-06 | 5.79E-05 | Prostaglandin E2 receptor EP4 subtype |
| Unigene70457_S2A | 48.36 | 13.03 | -1.89 | 4.17E-28 | 3.77E-26 | NFAT activation molecule 1-like |
| Unigene63225_S2A | 16.06 | 4.34 | -1.89 | 3.02E-10 | 8.59E-09 | - |
| Unigene37143_S2A | 8.03 | 2.17 | -1.89 | 9.59E-06 | 1.38E-04 | - |
| Unigene71378_S2A | 8.03 | 2.17 | -1.89 | 9.59E-06 | 1.38E-04 | - |
| Unigene4730_S2A | 11.35 | 3.08 | -1.88 | 1.34E-07 | 2.70E-06 | - |
| Unigene73620_S2A | 49.23 | 13.39 | -1.88 | 2.53E-28 | 2.31E-26 | - |
| Unigene10688_S2A | 9.95 | 2.71 | -1.88 | 8.82E-07 | 1.57E-05 | - |
| Unigene10765_S2A | 13.27 | 3.62 | -1.87 | 1.26E-08 | 2.97E-07 | Sterile alpha and TIR motif-containing protein 1 |
| Unigene3460_S2A | 6.63 | 1.81 | -1.87 | 6.47E-05 | 7.82E-04 | - |
| Unigene291_S2A | 102.48 | 28.22 | -1.86 | 1.79E-56 | 2.72E-54 | - |
| Unigene35545_S2A | 22.87 | 6.33 | -1.85 | 1.05E-13 | 4.52E-12 | - |
| Unigene25846_S2A | 428.60 | 118.69 | -1.85 | 4.59E-229 | 1.17E-226 | Small EDRK-rich factor 2, partial |
| Unigene26440_S2A | 25.49 | 7.06 | -1.85 | 4.05E-15 | 2.00E-13 | Leucine-rich repeat-containing protein 25 precursor |
| CL777.Contig1_S2A | 99.86 | 27.86 | -1.84 | 2.42E-54 | 3.56E-52 | Collagen alpha-1(I) chain precursor |
| Unigene10398_S2A | 78.21 | 21.89 | -1.84 | 8.74E-43 | 1.08E-40 | Dual specificity phosphatase 1 |
| Unigene71895_S2A | 51.68 | 14.47 | -1.84 | 8.59E-29 | 7.87E-27 | Tripartite motif-containing 6 and tripartite motif-containing 34 |
| Unigene20707_S2A | 11.00 | 3.08 | -1.84 | 3.30E-07 | 6.32E-06 | - |
| Unigene17059_S2A | 14.84 | 4.16 | -1.83 | 2.94E-09 | 7.47E-08 | - |
| Unigene66452_S2A | 38.06 | 10.67 | -1.83 | 1.41E-21 | 1.02E-19 | - |
| Unigene10961_S2A | 90.96 | 25.51 | -1.83 | 2.21E-49 | 3.11E-47 | - |
| CL2843.Contig1_S2A | 34.04 | 9.59 | -1.83 | 2.24E-19 | 1.46E-17 | Fc gamma 2 receptor precursor |
| Unigene42191_S2A | 9.60 | 2.71 | -1.82 | 2.17E-06 | 3.54E-05 | Glutamate [NMDA] receptor subunit epsilon-4-like |
| Unigene41432_S2A | 9.60 | 2.71 | -1.82 | 2.17E-06 | 3.54E-05 | - |
| Unigene67183_S2A | 9.60 | 2.71 | -1.82 | 2.17E-06 | 3.53E-05 | - |
| Unigene53681_S2A | 7.68 | 2.17 | -1.82 | 2.37E-05 | 3.16E-04 | - |
| Unigene49074_S2A | 7.68 | 2.17 | -1.82 | 2.37E-05 | 3.16E-04 | KIAA0542 protein-like |
| Unigene63105_S2A | 16.59 | 4.70 | -1.82 | 4.37E-10 | 1.20E-08 | Hypothetical protein EGM_04025, partial |
| Unigene12991_S2A | 6.98 | 1.99 | -1.81 | 6.15E-05 | 7.48E-04 | - |
| Unigene37359_S2A | 10.13 | 2.89 | -1.81 | 1.32E-06 | 2.25E-05 | Zinc finger protein 408 |
| Unigene66881_S2A | 13.27 | 3.80 | -1.80 | 2.95E-08 | 6.54E-07 | - |
| Unigene16728_S2A | 24.62 | 7.06 | -1.80 | 3.65E-14 | 1.66E-12 | - |
| Unigene14559_S2A | 9.43 | 2.71 | -1.80 | 3.38E-06 | 5.32E-05 | - |
| Unigene65690_S2A | 7.51 | 2.17 | -1.79 | 3.71E-05 | 4.76E-04 | Protein kinase C eta type isoform 1 |
| Unigene70824_S2A | 7.51 | 2.17 | -1.79 | 3.71E-05 | 4.75E-04 | - |
| Unigene80607_S2A | 65.12 | 18.82 | -1.79 | 1.00E-34 | 1.05E-32 | - |
| Unigene36111_S2A | 18.16 | 5.25 | -1.79 | 1.01E-10 | 3.04E-09 | - |
| Unigene29427_S2A | 18.16 | 5.25 | -1.79 | 1.01E-10 | 3.03E-09 | - |
| Unigene16303_S2A | 29.33 | 8.50 | -1.79 | 2.05E-16 | 1.11E-14 | - |
| Unigene71551_S2A | 60.41 | 17.55 | -1.78 | 3.84E-32 | 3.89E-30 | G protein-coupled receptor kinase GRK4, alpha splice-like |
| Unigene25879_S2A | 56.39 | 16.46 | -1.78 | 5.94E-30 | 5.69E-28 | - |
| Unigene16507_S2A | 12.40 | 3.62 | -1.78 | 1.16E-07 | 2.36E-06 | - |
| Unigene59_S2A | 20.43 | 5.97 | -1.77 | 9.22E-12 | 3.14E-10 | Inositol-trisphosphate 3-kinase C |
| Unigene51866_S2A | 12.92 | 3.80 | -1.77 | 7.08E-08 | 1.49E-06 | - |
| Unigene21414_S2A | 10.47 | 3.08 | -1.77 | 1.24E-06 | 2.14E-05 | - |
| Unigene10700_S2A | 39.28 | 11.58 | -1.76 | 4.06E-21 | 2.89E-19 | Calcium exchanger 6 precursor |
| Unigene11946_S2A | 21.47 | 6.33 | -1.76 | 3.46E-12 | 1.23E-10 | - |
| Unigene20917_S2A | 8.55 | 2.53 | -1.76 | 1.35E-05 | 1.89E-04 | Zinc finger protein 579-like |
| Unigene35388_S2A | 35.44 | 10.49 | -1.76 | 4.15E-19 | 2.65E-17 | - |
| Unigene9225_S2A | 359.99 | 106.56 | -1.76 | 3.45E-179 | 8.43E-177 | - |
| Unigene16193_S2A | 7.33 | 2.17 | -1.76 | 5.77E-05 | 7.07E-04 | - |
| Unigene10199_S2A | 198.33 | 58.80 | -1.75 | 2.13E-99 | 4.24E-97 | Endonuclease reverse transcriptase |
| Unigene41268_S2A | 27.41 | 8.14 | -1.75 | 4.67E-15 | 2.29E-13 | - |
| Unigene11143_S2A | 52.20 | 15.56 | -1.75 | 3.12E-27 | 2.71E-25 | SH3-domain binding protein 1-like |
| Unigene70297_S2A | 7.86 | 2.35 | -1.74 | 3.47E-05 | 4.47E-04 | - |
| Unigene11466_S2A | 18.16 | 5.43 | -1.74 | 2.26E-10 | 6.54E-09 | Protor-2 |
| Unigene11945_S2A | 26.54 | 7.96 | -1.74 | 1.79E-14 | 8.30E-13 | - |
| Unigene81687_S2A | 13.79 | 4.16 | -1.73 | 4.03E-08 | 8.75E-07 | - |
| Unigene41482_S2A | 86.94 | 26.23 | -1.73 | 1.09E-43 | 1.39E-41 | - |
| Unigene26684_S2A | 113.13 | 34.19 | -1.73 | 3.15E-56 | 4.73E-54 | - |
| Unigene66735_S2A | 21.47 | 6.51 | -1.72 | 7.65E-12 | 2.62E-10 | - |
| Unigene78061_S2A | 94.27 | 28.59 | -1.72 | 5.69E-47 | 7.77E-45 | - |
| Unigene25786_S2A | 144.38 | 43.96 | -1.72 | 1.12E-70 | 1.97E-68 | Human CMAP-like |
| CL1732.Contig1_S2A | 26.71 | 8.14 | -1.71 | 2.56E-14 | 1.17E-12 | Ubiquitin specific protease 9, Y-linked |
| Unigene76832_S2A | 11.87 | 3.62 | -1.71 | 4.26E-07 | 7.99E-06 | - |
| Unigene26514_S2A | 11.87 | 3.62 | -1.71 | 4.26E-07 | 7.98E-06 | HCG2007181 |
| Unigene71270_S2A | 146.82 | 44.87 | -1.71 | 1.52E-71 | 2.70E-69 | - |
| Unigene10391_S2A | 7.68 | 2.35 | -1.71 | 5.35E-05 | 6.60E-04 | - |
| Unigene65550_S2A | 10.65 | 3.26 | -1.71 | 1.79E-06 | 2.96E-05 | - |
| Unigene64713_S2A | 9.43 | 2.89 | -1.71 | 7.54E-06 | 1.10E-04 | - |
| Unigene26437_S2A | 9.43 | 2.89 | -1.71 | 7.54E-06 | 1.10E-04 | T-cell surface glycoprotein CD3 delta chain precursor |
| Unigene3657_S2A | 15.89 | 4.88 | -1.70 | 5.50E-09 | 1.36E-07 | - |
| Unigene78395_S2A | 12.92 | 3.98 | -1.70 | 1.56E-07 | 3.12E-06 | HCG2021286 |
| Unigene25233_S2A | 236.73 | 73.27 | -1.69 | 1.54E-112 | 3.23E-110 | L-threonine deaminase |
| Unigene72773_S2A | 9.95 | 3.08 | -1.69 | 4.54E-06 | 6.96E-05 | - |
| Unigene71031_S2A | 9.95 | 3.08 | -1.69 | 4.54E-06 | 6.95E-05 | - |
| Unigene36109_S2A | 49.06 | 15.20 | -1.69 | 1.29E-24 | 1.02E-22 | - |
| Unigene66088_S2A | 8.73 | 2.71 | -1.69 | 1.93E-05 | 2.63E-04 | - |
| Unigene72132_S2A | 15.71 | 4.88 | -1.69 | 8.39E-09 | 2.04E-07 | - |
| Unigene66716_S2A | 12.22 | 3.80 | -1.69 | 3.94E-07 | 7.46E-06 | Adenylate cyclase type 3 |
| Unigene59134_S2A | 12.22 | 3.80 | -1.69 | 3.94E-07 | 7.45E-06 | - |
| Unigene66114_S2A | 7.51 | 2.35 | -1.68 | 8.23E-05 | 9.63E-04 | Hypothetical protein EGK_20160 |
| Unigene36234_S2A | 7.51 | 2.35 | -1.68 | 8.23E-05 | 9.63E-04 | - |
| Unigene66419_S2A | 15.54 | 4.88 | -1.67 | 1.28E-08 | 3.00E-07 | - |
| Unigene26746_S2A | 158.00 | 49.75 | -1.67 | 3.52E-74 | 6.38E-72 | - |
| Unigene35405_S2A | 51.68 | 16.28 | -1.67 | 2.38E-25 | 1.93E-23 | - |
| Unigene16422_S2A | 18.33 | 5.79 | -1.66 | 6.94E-10 | 1.88E-08 | - |
| Unigene63020_S2A | 10.30 | 3.26 | -1.66 | 4.18E-06 | 6.45E-05 | - |
| Unigene16268_S2A | 10.30 | 3.26 | -1.66 | 4.18E-06 | 6.45E-05 | C5a anaphylatoxin chemotactic receptor C5L2 |
| Unigene39451_S2A | 8.55 | 2.71 | -1.66 | 2.95E-05 | 3.84E-04 | - |
| Unigene20893_S2A | 8.55 | 2.71 | -1.66 | 2.95E-05 | 3.84E-04 | Migration and invasion-inhibitory protein |
| Unigene71349_S2A | 8.55 | 2.71 | -1.66 | 2.95E-05 | 3.84E-04 | - |
| CL5194.Contig4_S2A | 15.36 | 4.88 | -1.65 | 1.94E-08 | 4.45E-07 | - |
| Unigene73107_S2A | 10.82 | 3.44 | -1.65 | 2.52E-06 | 4.06E-05 | - |
| CL185.Contig2_S2A | 14.14 | 4.52 | -1.65 | 8.02E-08 | 1.68E-06 | APG4 autophagy 4 homolog C |
| Unigene66833_S2A | 11.87 | 3.80 | -1.64 | 9.14E-07 | 1.62E-05 | - |
| Unigene41649_S2A | 45.74 | 14.65 | -1.64 | 3.49E-22 | 2.60E-20 | - |
| Unigene69314_S2A | 10.13 | 3.26 | -1.64 | 6.36E-06 | 9.40E-05 | - |
| Unigene77368_S2A | 7.86 | 2.53 | -1.64 | 7.51E-05 | 8.88E-04 | - |
| CL4586.Contig2_S2A | 7.86 | 2.53 | -1.64 | 7.51E-05 | 8.88E-04 | Myosin-Ig-like |
| Unigene61090_S2A | 13.44 | 4.34 | -1.63 | 2.01E-07 | 3.96E-06 | - |
| CL300.Contig1_S2A | 13.44 | 4.34 | -1.63 | 2.01E-07 | 3.95E-06 | - |
| Unigene77867_S2A | 21.30 | 6.88 | -1.63 | 5.28E-11 | 1.64E-09 | - |
| Unigene37079_S2A | 53.77 | 17.37 | -1.63 | 1.46E-25 | 1.21E-23 | - |
| Unigene27022_S2A | 22.87 | 7.42 | -1.62 | 1.19E-11 | 3.98E-10 | T-cell receptor gamma chain V-J-C region |
| Unigene65472_S2A | 77.51 | 25.15 | -1.62 | 5.93E-36 | 6.40E-34 | Leupaxin |
| Unigene21931_S2A | 8.90 | 2.89 | -1.62 | 2.69E-05 | 3.53E-04 | Acyl-CoA synthetase long-chain family member 6 isoform |
| Unigene66858_S2A | 77.34 | 25.15 | -1.62 | 8.88E-36 | 9.50E-34 | Polymerase 1 |
| CL513.Contig1_S2A | 988.66 | 321.68 | -1.62 | 0 | 0 | MHC class II antigen precursor |
| Unigene21309_S2A | 16.59 | 5.43 | -1.61 | 9.84E-09 | 2.35E-07 | - |
| Unigene71772_S2A | 68.44 | 22.43 | -1.61 | 1.57E-31 | 1.56E-29 | - |
| Unigene21338_S2A | 10.47 | 3.44 | -1.61 | 5.79E-06 | 8.64E-05 | - |
| Unigene59510_S2A | 11.00 | 3.62 | -1.60 | 3.48E-06 | 5.46E-05 | - |
| Unigene76373_S2A | 68.09 | 22.43 | -1.60 | 3.50E-31 | 3.46E-29 | Transposase |
| Unigene26354_S2A | 236.04 | 77.80 | -1.60 | 7.51E-104 | 1.53E-101 | - |
| Unigene59701_S2A | 11.52 | 3.80 | -1.60 | 2.09E-06 | 3.44E-05 | RCG60353 |
| Unigene7093_S2A | 39.46 | 13.03 | -1.60 | 1.14E-18 | 7.10E-17 | - |
| Unigene590_S2A | 12.05 | 3.98 | -1.60 | 1.26E-06 | 2.16E-05 | - |
| Unigene60091_S2A | 15.89 | 5.25 | -1.60 | 2.45E-08 | 5.48E-07 | - |
| Unigene26322_S2A | 87.47 | 28.95 | -1.60 | 2.24E-39 | 2.56E-37 | Galectin-9 isoform 2 |
| Unigene71242_S2A | 13.09 | 4.34 | -1.59 | 4.58E-07 | 8.55E-06 | - |
| Unigene26422_S2A | 48.01 | 15.92 | -1.59 | 2.84E-22 | 2.12E-20 | C-C motif chemokine 23 precursor |
| Unigene44764_S2A | 17.98 | 5.97 | -1.59 | 3.29E-09 | 8.30E-08 | - |
| Unigene42160_S2A | 27.76 | 9.23 | -1.59 | 1.86E-13 | 7.70E-12 | Indoleamine 2,3-dioxygenase 1 |
| Unigene15848_S2A | 9.25 | 3.08 | -1.59 | 2.43E-05 | 3.23E-04 | - |
| Unigene25625_S2A | 9.78 | 3.26 | -1.58 | 1.46E-05 | 2.03E-04 | - |
| Unigene60126_S2A | 30.38 | 10.13 | -1.58 | 1.55E-14 | 7.25E-13 | - |
| Unigene71697_S2A | 117.15 | 39.08 | -1.58 | 1.21E-51 | 1.75E-49 | - |
| Unigene77845_S2A | 20.60 | 6.88 | -1.58 | 2.69E-10 | 7.73E-09 | - |
| Unigene20998_S2A | 16.24 | 5.43 | -1.58 | 2.22E-08 | 5.01E-07 | - |
| Unigene5096_S2A | 11.35 | 3.80 | -1.58 | 3.15E-06 | 5.00E-05 | HCG2040658 |
| Unigene71827_S2A | 23.22 | 7.78 | -1.58 | 2.22E-11 | 7.21E-10 | CCR4-NOT transcription complex subunit 2 |
| Unigene66891_S2A | 12.40 | 4.16 | -1.58 | 1.14E-06 | 1.98E-05 | - |
| Unigene11312_S2A | 12.40 | 4.16 | -1.58 | 1.14E-06 | 1.98E-05 | BM-018 |
| Unigene70584_S2A | 18.33 | 6.15 | -1.58 | 2.98E-09 | 7.57E-08 | - |
| Unigene66548_S2A | 31.25 | 10.49 | -1.57 | 8.60E-15 | 4.13E-13 | Sphingosine kinase 1 |
| Unigene48875_S2A | 19.38 | 6.51 | -1.57 | 1.09E-09 | 2.92E-08 | - |
| Unigene10613_S2A | 13.97 | 4.70 | -1.57 | 2.50E-07 | 4.86E-06 | - |
| Unigene11119_S2A | 20.95 | 7.06 | -1.57 | 2.44E-10 | 7.05E-09 | Dickkopf-like protein 1 precursor |
| Unigene11211_S2A | 22.00 | 7.42 | -1.57 | 8.99E-11 | 2.73E-09 | Calcium channel, voltage-dependent, T type, alpha 1H subunit |
| Unigene66485_S2A | 8.55 | 2.89 | -1.56 | 6.13E-05 | 7.48E-04 | - |
| Unigene61561_S2A | 8.55 | 2.89 | -1.56 | 6.13E-05 | 7.48E-04 | - |
| Unigene16668_S2A | 8.55 | 2.89 | -1.56 | 6.13E-05 | 7.48E-04 | - |
| Unigene65260_S2A | 8.55 | 2.89 | -1.56 | 6.13E-05 | 7.47E-04 | - |
| Unigene26111_S2A | 8.55 | 2.89 | -1.56 | 6.13E-05 | 7.47E-04 | - |
| Unigene515_S2A | 44.87 | 15.20 | -1.56 | 2.23E-20 | 1.50E-18 | - |
| Unigene10874_S2A | 36.84 | 12.48 | -1.56 | 5.50E-17 | 3.06E-15 | - |
| CL2754.Contig1_S2A | 10.13 | 3.44 | -1.56 | 1.31E-05 | 1.85E-04 | Dual specificity protein kinase CLK4 |
| Unigene35498_S2A | 11.17 | 3.80 | -1.56 | 4.73E-06 | 7.22E-05 | Protein FAM122A-like |
| Unigene25239_S2A | 16.41 | 5.61 | -1.55 | 3.00E-08 | 6.64E-07 | AT rich interactive domain 1A-like |
| Unigene12115_S2A | 98.81 | 34.01 | -1.54 | 4.39E-42 | 5.39E-40 | - |
| Unigene65294_S2A | 26.19 | 9.05 | -1.53 | 3.33E-12 | 1.18E-10 | - |
| Unigene6274_S2A | 9.43 | 3.26 | -1.53 | 3.29E-05 | 4.26E-04 | - |
| Unigene7824_S2A | 10.47 | 3.62 | -1.53 | 1.18E-05 | 1.67E-04 | - |
| Unigene21464_S2A | 8.90 | 3.08 | -1.53 | 5.50E-05 | 6.75E-04 | - |
| Unigene42232_S2A | 90.96 | 31.48 | -1.53 | 1.36E-38 | 1.54E-36 | - |
| Unigene71775_S2A | 169.35 | 58.62 | -1.53 | 2.32E-70 | 4.03E-68 | - |
| Unigene25618_S2A | 58.49 | 20.26 | -1.53 | 2.37E-25 | 1.93E-23 | B-lymphocyte antigen CD20 |
| Unigene65737_S2A | 39.63 | 13.75 | -1.53 | 1.23E-17 | 7.24E-16 | - |
| Unigene5994_S2A | 35.44 | 12.30 | -1.53 | 6.47E-16 | 3.38E-14 | Tumor necrosis factor receptor superfamily member 6 precursor |
| Unigene60446_S2A | 15.54 | 5.43 | -1.52 | 1.10E-07 | 2.24E-06 | - |
| Unigene66375_S2A | 41.90 | 14.65 | -1.52 | 2.27E-18 | 1.38E-16 | - |
| Unigene66722_S2A | 13.44 | 4.70 | -1.52 | 8.29E-07 | 1.48E-05 | - |
| Unigene11886_S2A | 12.92 | 4.52 | -1.52 | 1.38E-06 | 2.35E-05 | Solute carrier family 12, member 7-like |
| Unigene36078_S2A | 12.40 | 4.34 | -1.51 | 2.29E-06 | 3.71E-05 | - |
| Unigene391_S2A | 38.76 | 13.57 | -1.51 | 4.41E-17 | 2.46E-15 | - |
| Unigene50960_S2A | 103.88 | 36.37 | -1.51 | 3.60E-43 | 4.53E-41 | - |
| Unigene35770_S2A | 11.87 | 4.16 | -1.51 | 3.80E-06 | 5.91E-05 | - |
| Unigene11265_S2A | 57.79 | 20.26 | -1.51 | 1.12E-24 | 8.94E-23 | GH3 domain-containing protein precursor |
| Unigene25839_S2A | 41.73 | 14.65 | -1.51 | 3.34E-18 | 2.01E-16 | Mediator of RNA polymerase II transcription subunit 15 |
| Unigene17207_S2A | 53.07 | 18.64 | -1.51 | 9.45E-23 | 7.18E-21 | - |
| Unigene66892_S2A | 10.30 | 3.62 | -1.51 | 1.76E-05 | 2.42E-04 | - |
| Unigene48464_S2A | 9.78 | 3.44 | -1.51 | 2.94E-05 | 3.84E-04 | Leucine-rich repeat-containing protein 39 |
| Unigene195_S2A | 9.78 | 3.44 | -1.51 | 2.94E-05 | 3.83E-04 | - |
| Unigene73878_S2A | 44.17 | 15.56 | -1.51 | 4.16E-19 | 2.65E-17 | - |
| Unigene16121_S2A | 9.25 | 3.26 | -1.50 | 4.91E-05 | 6.11E-04 | - |
| Unigene11260_S2A | 33.35 | 11.76 | -1.50 | 9.19E-15 | 4.40E-13 | - |
| Unigene37570_S2A | 8.73 | 3.08 | -1.50 | 8.21E-05 | 9.62E-04 | - |
| Unigene21710_S2A | 15.89 | 5.61 | -1.50 | 9.83E-08 | 2.02E-06 | - |
| Unigene25652_S2A | 30.73 | 10.86 | -1.50 | 1.10E-13 | 4.72E-12 | - |
| Unigene59960_S2A | 22.00 | 7.78 | -1.50 | 3.53E-10 | 9.90E-09 | - |
| Unigene26914_S2A | 69.83 | 24.79 | -1.49 | 5.11E-29 | 4.76E-27 | Immunoglobulin delta heavy chain constant region |
| Unigene36129_S2A | 64.60 | 22.98 | -1.49 | 6.99E-27 | 6.06E-25 | - |
| Unigene77685_S2A | 16.76 | 5.97 | -1.49 | 5.30E-08 | 1.14E-06 | - |
| Unigene26804_S2A | 10.65 | 3.80 | -1.49 | 1.57E-05 | 2.16E-04 | - |
| Unigene36237_S2A | 20.78 | 7.42 | -1.49 | 1.41E-09 | 3.71E-08 | - |
| Unigene66524_S2A | 25.84 | 9.23 | -1.49 | 1.42E-11 | 4.71E-10 | - |
| Unigene78733_S2A | 10.13 | 3.62 | -1.48 | 2.61E-05 | 3.44E-04 | - |
| Unigene77914_S2A | 15.19 | 5.43 | -1.48 | 2.40E-07 | 4.69E-06 | - |
| Unigene11099_S2A | 9.08 | 3.26 | -1.48 | 7.29E-05 | 8.66E-04 | - |
| Unigene10905_S2A | 48.88 | 17.55 | -1.48 | 1.82E-20 | 1.23E-18 | Signal transducer and activator of transcription 4 |
| Unigene10463_S2A | 21.65 | 7.78 | -1.48 | 7.66E-10 | 2.07E-08 | - |
| Unigene9784_S2A | 72.98 | 26.23 | -1.48 | 9.89E-30 | 9.41E-28 | - |
| Unigene27013_S2A | 34.22 | 12.30 | -1.48 | 9.68E-15 | 4.62E-13 | - |
| Unigene65564_S2A | 12.57 | 4.52 | -1.48 | 3.02E-06 | 4.81E-05 | N(G),N(G)-dimethylarginine dimethylaminohydrolase 2 |
| Unigene35893_S2A | 24.09 | 8.68 | -1.47 | 9.28E-11 | 2.81E-09 | - |
| Unigene4929_S2A | 11.52 | 4.16 | -1.47 | 8.35E-06 | 1.21E-04 | FAD-AMP lyase |
| Unigene65805_S2A | 18.51 | 6.69 | -1.47 | 1.54E-08 | 3.59E-07 | - |
| CL2135.Contig1_S2A | 22.52 | 8.14 | -1.47 | 4.14E-10 | 1.15E-08 | Rhomboid domain-containing protein 3 |
| Unigene36654_S2A | 19.03 | 6.88 | -1.47 | 9.34E-09 | 2.25E-07 | - |
| CL685.Contig1_S2A | 11.00 | 3.98 | -1.47 | 1.39E-05 | 1.95E-04 | - |
| Unigene36721_S2A | 14.49 | 5.25 | -1.46 | 5.87E-07 | 1.08E-05 | - |
| Unigene16331_S2A | 24.44 | 8.87 | -1.46 | 8.27E-11 | 2.52E-09 | - |
| Unigene11096_S2A | 10.47 | 3.80 | -1.46 | 2.32E-05 | 3.10E-04 | - |
| Unigene59243_S2A | 26.89 | 9.77 | -1.46 | 1.01E-11 | 3.41E-10 | Protein ELYS |
| Unigene36594_S2A | 12.92 | 4.70 | -1.46 | 2.68E-06 | 4.31E-05 | - |
| CL239.Contig1_S2A | 12.92 | 4.70 | -1.46 | 2.68E-06 | 4.30E-05 | - |
| Unigene14710_S2A | 9.95 | 3.62 | -1.46 | 3.86E-05 | 4.94E-04 | - |
| Unigene71954_S2A | 44.69 | 16.28 | -1.46 | 1.80E-18 | 1.10E-16 | - |
| Unigene72855_S2A | 19.38 | 7.06 | -1.46 | 8.31E-09 | 2.02E-07 | - |
| CL1634.Contig1_S2A | 9.43 | 3.44 | -1.45 | 6.45E-05 | 7.82E-04 | Ras-related GTP-binding protein B-like isoform 2 |
| Unigene13045_S2A | 9.43 | 3.44 | -1.45 | 6.45E-05 | 7.81E-04 | - |
| Unigene10773_S2A | 9.43 | 3.44 | -1.45 | 6.45E-05 | 7.81E-04 | Transcriptional repressor p66-alpha |
| Unigene66795_S2A | 60.41 | 22.07 | -1.45 | 2.49E-24 | 1.96E-22 | - |
| Unigene11263_S2A | 227.31 | 83.41 | -1.45 | 2.01E-86 | 3.80E-84 | - |
| Unigene26521_S2A | 10.82 | 3.98 | -1.44 | 2.05E-05 | 2.78E-04 | NADPH oxidase cytosolic protein p47phox |
| Unigene77360_S2A | 10.82 | 3.98 | -1.44 | 2.05E-05 | 2.78E-04 | - |
| Unigene14239_S2A | 12.74 | 4.70 | -1.44 | 3.93E-06 | 6.09E-05 | - |
| Unigene11838_S2A | 27.93 | 10.31 | -1.44 | 7.06E-12 | 2.43E-10 | PFOXic |
| Unigene16050_S2A | 12.22 | 4.52 | -1.43 | 6.53E-06 | 9.63E-05 | - |
| Unigene21732_S2A | 38.58 | 14.29 | -1.43 | 8.67E-16 | 4.49E-14 | - |
| Unigene11475_S2A | 26.36 | 9.77 | -1.43 | 3.13E-11 | 9.96E-10 | Zinc finger protein 385A-like |
| Unigene26211_S2A | 95.67 | 35.46 | -1.43 | 7.70E-37 | 8.36E-35 | - |
| Unigene26912_S2A | 396.83 | 147.09 | -1.43 | 3.13E-147 | 7.34E-145 | High affinity IgE receptor gamma subunit |
| Unigene10405_S2A | 30.73 | 11.40 | -1.43 | 7.65E-13 | 2.96E-11 | - |
| Unigene26069_S2A | 134.78 | 50.12 | -1.43 | 5.70E-51 | 8.13E-49 | Calpain 1 catalytic subunit |
| Unigene70518_S2A | 20.43 | 7.60 | -1.43 | 5.79E-09 | 1.43E-07 | Hypothetical protein I79_023472 |
| CL2807.Contig1_S2A | 13.62 | 5.07 | -1.43 | 2.09E-06 | 3.44E-05 | Neighbor of COX4 |
| Unigene70973_S2A | 19.38 | 7.24 | -1.42 | 1.57E-08 | 3.65E-07 | IKAROS family zinc finger 2 |
| Unigene16809_S2A | 10.65 | 3.98 | -1.42 | 3.01E-05 | 3.91E-04 | - |
| Unigene66295_S2A | 10.65 | 3.98 | -1.42 | 3.01E-05 | 3.91E-04 | - |
| Unigene76731_S2A | 15.01 | 5.61 | -1.42 | 6.74E-07 | 1.22E-05 | Parafibromin |
| CL4288.Contig1_S2A | 114.70 | 42.88 | -1.42 | 2.72E-43 | 3.44E-41 | Choline transporter-like protein 2 |
| Unigene60240_S2A | 65.82 | 24.61 | -1.42 | 1.56E-25 | 1.28E-23 | - |
| CL1405.Contig1_S2A | 91.31 | 34.19 | -1.42 | 1.04E-34 | 1.08E-32 | Vasodilator-stimulated phosphoprotein |
| Unigene41967_S2A | 14.49 | 5.43 | -1.42 | 1.12E-06 | 1.94E-05 | - |
| Unigene26010_S2A | 124.48 | 46.68 | -1.42 | 1.27E-46 | 1.71E-44 | Similar to hypothetical protein FLJ10154, isoform CRA_a |
| Unigene28066_S2A | 12.05 | 4.52 | -1.41 | 9.56E-06 | 1.37E-04 | - |
| Unigene52623_S2A | 20.25 | 7.60 | -1.41 | 8.43E-09 | 2.04E-07 | - |
| Unigene80890_S2A | 56.39 | 21.17 | -1.41 | 5.73E-22 | 4.21E-20 | Macrophage colony-stimulating factor 1 receptor precursor |
| Unigene11286_S2A | 65.99 | 24.79 | -1.41 | 2.01E-25 | 1.64E-23 | - |
| Unigene82134_S2A | 99.69 | 37.45 | -1.41 | 1.44E-37 | 1.60E-35 | - |
| Unigene25837_S2A | 24.09 | 9.05 | -1.41 | 3.32E-10 | 9.34E-09 | Collagen alpha-2(VI) chain, partial |
| Unigene25911_S2A | 378.32 | 142.39 | -1.41 | 2.93E-137 | 6.60E-135 | Solute carrier family 40 member 1 |
| Unigene21107_S2A | 26.89 | 10.13 | -1.41 | 3.56E-11 | 1.13E-09 | - |
| Unigene59836_S2A | 32.65 | 12.30 | -1.41 | 2.87E-13 | 1.16E-11 | - |
| Unigene59555_S2A | 15.36 | 5.79 | -1.41 | 5.94E-07 | 1.09E-05 | - |
| Unigene72726_S2A | 28.28 | 10.67 | -1.41 | 1.17E-11 | 3.92E-10 | - |
| Unigene4736_S2A | 23.92 | 9.05 | -1.40 | 4.81E-10 | 1.32E-08 | - |
| Unigene25234_S2A | 390.72 | 148.00 | -1.40 | 2.60E-140 | 5.97E-138 | - |
| Unigene53752_S2A | 21.47 | 8.14 | -1.40 | 3.99E-09 | 1.00E-07 | - |
| Unigene72082_S2A | 14.32 | 5.43 | -1.40 | 1.63E-06 | 2.71E-05 | - |
| Unigene71480_S2A | 22.87 | 8.68 | -1.40 | 1.30E-09 | 3.44E-08 | Transformer-2 protein homolog alpha |
| Unigene26831_S2A | 153.81 | 58.44 | -1.40 | 4.39E-56 | 6.51E-54 | TYRO protein tyrosine kinase-binding protein |
| Unigene646_S2A | 11.87 | 4.52 | -1.39 | 1.40E-05 | 1.95E-04 | - |
| CL1104.Contig1_S2A | 11.87 | 4.52 | -1.39 | 1.40E-05 | 1.95E-04 | - |
| Unigene37717_S2A | 16.59 | 6.33 | -1.39 | 2.79E-07 | 5.40E-06 | - |
| Unigene9142_S2A | 23.22 | 8.87 | -1.39 | 1.15E-09 | 3.05E-08 | - |
| Unigene71340_S2A | 44.52 | 17.01 | -1.39 | 3.15E-17 | 1.79E-15 | - |
| Unigene26955_S2A | 81.36 | 31.12 | -1.39 | 3.76E-30 | 3.63E-28 | Dynactin subunit 1 |
| Unigene26854_S2A | 41.55 | 15.92 | -1.38 | 4.16E-16 | 2.19E-14 | V-type proton ATPase subunit B, brain isoform |
| Unigene10403_S2A | 20.25 | 7.78 | -1.38 | 1.56E-08 | 3.63E-07 | HCG1988652 |
| Unigene77268_S2A | 12.22 | 4.70 | -1.38 | 1.22E-05 | 1.72E-04 | - |
| Unigene59507_S2A | 71.93 | 27.68 | -1.38 | 1.34E-26 | 1.14E-24 | - |
| CL3205.Contig1_S2A | 15.01 | 5.79 | -1.37 | 1.25E-06 | 2.15E-05 | - |
| Unigene4914_S2A | 20.60 | 7.96 | -1.37 | 1.37E-08 | 3.21E-07 | - |
| Unigene66563_S2A | 58.49 | 22.62 | -1.37 | 9.15E-22 | 6.70E-20 | - |
| Unigene11170_S2A | 350.39 | 135.69 | -1.37 | 9.45E-122 | 2.04E-119 | Galectin-3 |
| Unigene77715_S2A | 33.52 | 13.03 | -1.36 | 5.19E-13 | 2.05E-11 | - |
| Unigene10706_S2A | 117.67 | 45.77 | -1.36 | 8.84E-42 | 1.07E-39 | KIAA0476 protein-like |
| Unigene66908_S2A | 39.98 | 15.56 | -1.36 | 3.29E-15 | 1.63E-13 | - |
| Unigene59245_S2A | 35.79 | 13.93 | -1.36 | 9.19E-14 | 3.98E-12 | - |
| Unigene26223_S2A | 191.87 | 74.72 | -1.36 | 6.51E-67 | 1.08E-64 | Allograft inflammatory factor 1 |
| Unigene35705_S2A | 26.01 | 10.13 | -1.36 | 2.23E-10 | 6.48E-09 | - |
| Unigene21146_S2A | 23.22 | 9.05 | -1.36 | 2.09E-09 | 5.43E-08 | - |
| Unigene25463_S2A | 16.24 | 6.33 | -1.36 | 5.83E-07 | 1.07E-05 | - |
| Unigene71178_S2A | 12.05 | 4.70 | -1.36 | 1.77E-05 | 2.43E-04 | - |
| Unigene66909_S2A | 12.05 | 4.70 | -1.36 | 1.77E-05 | 2.43E-04 | - |
| Unigene13980_S2A | 19.90 | 7.78 | -1.35 | 3.25E-08 | 7.14E-07 | - |
| Unigene38883_S2A | 23.57 | 9.23 | -1.35 | 1.84E-09 | 4.78E-08 | - |
| Unigene71958_S2A | 14.32 | 5.61 | -1.35 | 2.99E-06 | 4.76E-05 | Lymphocyte antigen 96 precursor |
| Unigene59330_S2A | 70.18 | 27.50 | -1.35 | 2.71E-25 | 2.19E-23 | - |
| Unigene80351_S2A | 11.52 | 4.52 | -1.35 | 2.94E-05 | 3.83E-04 | Fructose-1,6-bisphosphatase 1 |
| Unigene65734_S2A | 11.52 | 4.52 | -1.35 | 2.94E-05 | 3.83E-04 | Coiled-coil domain-containing protein 85B |
| Unigene71648_S2A | 58.49 | 22.98 | -1.35 | 2.98E-21 | 2.12E-19 | - |
| Unigene11078_S2A | 13.79 | 5.43 | -1.34 | 4.94E-06 | 7.49E-05 | Lymphocyte cytosolic protein 2 |
| Unigene73509_S2A | 21.12 | 8.32 | -1.34 | 1.52E-08 | 3.54E-07 | - |
| Unigene11765_S2A | 33.00 | 13.03 | -1.34 | 1.53E-12 | 5.71E-11 | - |
| Unigene25938_S2A | 332.06 | 131.35 | -1.34 | 1.28E-111 | 2.67E-109 | Signal-induced proliferation-associated protein 1 |
| Unigene66310_S2A | 32.47 | 12.85 | -1.34 | 2.50E-12 | 8.96E-11 | - |
| Unigene77256_S2A | 15.54 | 6.15 | -1.34 | 1.38E-06 | 2.35E-05 | UBX domain-containing protein 8 |
| Unigene65415_S2A | 84.85 | 33.65 | -1.33 | 1.11E-29 | 1.05E-27 | - |
| Unigene59247_S2A | 32.30 | 12.85 | -1.33 | 3.57E-12 | 1.26E-10 | - |
| Unigene36770_S2A | 12.74 | 5.07 | -1.33 | 1.35E-05 | 1.89E-04 | - |
| Unigene73855_S2A | 11.35 | 4.52 | -1.33 | 4.24E-05 | 5.34E-04 | - |
| Unigene19294_S2A | 16.76 | 6.69 | -1.32 | 6.39E-07 | 1.16E-05 | - |
| Unigene5916_S2A | 16.76 | 6.69 | -1.32 | 6.39E-07 | 1.16E-05 | - |
| Unigene66541_S2A | 41.20 | 16.46 | -1.32 | 4.99E-15 | 2.45E-13 | - |
| Unigene21985_S2A | 38.93 | 15.56 | -1.32 | 2.81E-14 | 1.28E-12 | - |
| Unigene65717_S2A | 45.22 | 18.09 | -1.32 | 2.57E-16 | 1.36E-14 | - |
| Unigene25744_S2A | 27.58 | 11.04 | -1.32 | 1.65E-10 | 4.87E-09 | - |
| Unigene78209_S2A | 20.78 | 8.32 | -1.32 | 3.11E-08 | 6.87E-07 | - |
| Unigene71092_S2A | 15.36 | 6.15 | -1.32 | 1.99E-06 | 3.27E-05 | UPF0583 protein C15orf59 homolog |
| Unigene70634_S2A | 16.24 | 6.51 | -1.32 | 1.05E-06 | 1.83E-05 | - |
| Unigene42000_S2A | 18.51 | 7.42 | -1.32 | 1.80E-07 | 3.58E-06 | - |
| Unigene32225_S2A | 10.82 | 4.34 | -1.32 | 7.02E-05 | 8.38E-04 | 2'-5'-oligoadenylate synthase 2 |
| Unigene26429_S2A | 46.44 | 18.64 | -1.32 | 1.21E-16 | 6.64E-15 | SUN domain-containing protein 2 |
| Unigene26154_S2A | 19.38 | 7.78 | -1.32 | 9.58E-08 | 1.97E-06 | Insulin-like growth factor-binding protein 3 precursor |
| Unigene65993_S2A | 11.70 | 4.70 | -1.32 | 3.68E-05 | 4.73E-04 | - |
| Unigene66713_S2A | 201.82 | 81.24 | -1.31 | 1.21E-66 | 1.99E-64 | - |
| Unigene453_S2A | 124.83 | 50.30 | -1.31 | 8.21E-42 | 9.99E-40 | - |
| Unigene73481_S2A | 22.87 | 9.23 | -1.31 | 7.70E-09 | 1.88E-07 | - |
| Unigene36686_S2A | 26.89 | 10.86 | -1.31 | 3.83E-10 | 1.07E-08 | SH2 domain protein 2A |
| Unigene41363_S2A | 20.60 | 8.32 | -1.31 | 4.44E-08 | 9.61E-07 | - |
| Unigene9403_S2A | 40.68 | 16.46 | -1.31 | 1.44E-14 | 6.72E-13 | - |
| Unigene65604_S2A | 18.33 | 7.42 | -1.30 | 2.57E-07 | 5.00E-06 | Abnormal cell LINeage family member (lin-53)-like |
| Unigene59185_S2A | 43.30 | 17.55 | -1.30 | 2.23E-15 | 1.12E-13 | MAGUK p55 subfamily member 3-like |
| Unigene73496_S2A | 30.73 | 12.48 | -1.30 | 2.74E-11 | 8.82E-10 | - |
| Unigene25878_S2A | 78.39 | 31.84 | -1.30 | 1.62E-26 | 1.37E-24 | - |
| Unigene20980_S2A | 16.93 | 6.88 | -1.30 | 7.96E-07 | 1.42E-05 | - |
| Unigene26757_S2A | 13.79 | 5.61 | -1.30 | 8.85E-06 | 1.28E-04 | CD48 antigen precursor |
| Unigene25602_S2A | 23.57 | 9.59 | -1.30 | 5.85E-09 | 1.44E-07 | - |
| Unigene25812_S2A | 24.44 | 9.95 | -1.30 | 3.12E-09 | 7.91E-08 | RCG24770, isoform CRA_a |
| Unigene4764_S2A | 15.54 | 6.33 | -1.30 | 2.47E-06 | 3.99E-05 | GTP-binding protein REM 2 |
| Unigene59996_S2A | 42.60 | 17.37 | -1.29 | 5.13E-15 | 2.50E-13 | - |
| Unigene72632_S2A | 18.16 | 7.42 | -1.29 | 3.67E-07 | 6.97E-06 | - |
| Unigene66340_S2A | 18.16 | 7.42 | -1.29 | 3.67E-07 | 6.97E-06 | - |
| Unigene17056_S2A | 12.40 | 5.07 | -1.29 | 2.77E-05 | 3.64E-04 | - |
| Unigene71592_S2A | 53.95 | 22.07 | -1.29 | 1.61E-18 | 9.89E-17 | - |
| Unigene38183_S2A | 13.27 | 5.43 | -1.29 | 1.46E-05 | 2.03E-04 | - |
| Unigene65494_S2A | 40.15 | 16.46 | -1.29 | 4.08E-14 | 1.84E-12 | - |
| Unigene43948_S2A | 55.17 | 22.62 | -1.29 | 7.58E-19 | 4.75E-17 | - |
| CL4011.Contig1_S2A | 28.63 | 11.76 | -1.28 | 1.92E-10 | 5.62E-09 | - |
| Unigene10401_S2A | 28.63 | 11.76 | -1.28 | 1.92E-10 | 5.61E-09 | - |
| Unigene13128_S2A | 20.25 | 8.32 | -1.28 | 8.99E-08 | 1.86E-06 | - |
| Unigene36145_S2A | 20.25 | 8.32 | -1.28 | 8.99E-08 | 1.86E-06 | - |
| Unigene26565_S2A | 11.87 | 4.88 | -1.28 | 4.57E-05 | 5.74E-04 | Alternative |
| Unigene65562_S2A | 11.87 | 4.88 | -1.28 | 4.57E-05 | 5.74E-04 | - |
| Unigene78249_S2A | 11.87 | 4.88 | -1.28 | 4.57E-05 | 5.73E-04 | - |
| Unigene17220_S2A | 12.74 | 5.25 | -1.28 | 2.40E-05 | 3.19E-04 | - |
| Unigene71814_S2A | 19.73 | 8.14 | -1.28 | 1.47E-07 | 2.95E-06 | Thyroid adenoma-associated protein homolog, partial |
| Unigene25986_S2A | 81.53 | 33.65 | -1.28 | 8.27E-27 | 7.08E-25 | Tetraspanin-4 |
| Unigene12086_S2A | 21.47 | 8.87 | -1.28 | 4.15E-08 | 9.00E-07 | - |
| Unigene26134_S2A | 39.80 | 16.46 | -1.27 | 8.15E-14 | 3.54E-12 | Creatine kinase B-type |
| Unigene71711_S2A | 42.42 | 17.55 | -1.27 | 1.26E-14 | 5.92E-13 | - |
| Unigene77549_S2A | 11.35 | 4.70 | -1.27 | 7.54E-05 | 8.91E-04 | C-type lectin domain family 12 member A |
| Unigene63208_S2A | 15.71 | 6.51 | -1.27 | 3.05E-06 | 4.84E-05 | - |
| Unigene26424_S2A | 30.55 | 12.66 | -1.27 | 6.77E-11 | 2.07E-09 | - |
| Unigene59217_S2A | 13.09 | 5.43 | -1.27 | 2.08E-05 | 2.81E-04 | - |
| Unigene3854_S2A | 13.09 | 5.43 | -1.27 | 2.08E-05 | 2.80E-04 | Coronin-1C |
| Unigene20560_S2A | 84.15 | 34.92 | -1.27 | 2.24E-27 | 1.97E-25 | - |
| Unigene71476_S2A | 51.85 | 21.53 | -1.27 | 1.92E-17 | 1.12E-15 | - |
| Unigene22158_S2A | 51.33 | 21.35 | -1.27 | 3.11E-17 | 1.78E-15 | - |
| Unigene13126_S2A | 51.33 | 21.35 | -1.27 | 3.11E-17 | 1.77E-15 | - |
| Unigene59665_S2A | 23.04 | 9.59 | -1.26 | 1.67E-08 | 3.85E-07 | - |
| Unigene10921_S2A | 39.11 | 16.28 | -1.26 | 1.86E-13 | 7.71E-12 | - |
| Unigene11430_S2A | 19.55 | 8.14 | -1.26 | 2.08E-07 | 4.08E-06 | Tripartite motif-containing 47 |
| Unigene3881_S2A | 33.00 | 13.75 | -1.26 | 1.46E-11 | 4.85E-10 | Cardiotrophin-like cytokine factor 1-lik |
| Unigene59482_S2A | 31.25 | 13.03 | -1.26 | 5.11E-11 | 1.59E-09 | Uncharacterized protein LOC618591 |
| Unigene16235_S2A | 44.69 | 18.64 | -1.26 | 3.88E-15 | 1.92E-13 | Selenocysteine tRNA gene transcription-activating factor |
| Unigene36302_S2A | 38.58 | 16.10 | -1.26 | 3.02E-13 | 1.22E-11 | - |
| Unigene37499_S2A | 31.60 | 13.21 | -1.26 | 4.44E-11 | 1.39E-09 | - |
| Unigene11320_S2A | 19.90 | 8.32 | -1.26 | 1.80E-07 | 3.58E-06 | - |
| CL4781.Contig1_S2A | 58.31 | 24.42 | -1.26 | 3.76E-19 | 2.42E-17 | GTP-binding protein 2 isoform 1 |
| Unigene77087_S2A | 22.87 | 9.59 | -1.25 | 2.35E-08 | 5.29E-07 | - |
| Unigene26658_S2A | 329.96 | 138.41 | -1.25 | 2.00E-100 | 4.04E-98 | - |
| Unigene16957_S2A | 34.04 | 14.29 | -1.25 | 9.59E-12 | 3.25E-10 | - |
| Unigene44752_S2A | 12.92 | 5.43 | -1.25 | 2.95E-05 | 3.84E-04 | Complement component 4 binding protein, alpha chain precurso |
| Unigene81449_S2A | 30.55 | 12.85 | -1.25 | 1.17E-10 | 3.49E-09 | Autophagy-linked FYVE protein |
| Unigene26219_S2A | 39.11 | 16.46 | -1.25 | 3.20E-13 | 1.29E-11 | - |
| Unigene4526_S2A | 39.98 | 16.83 | -1.25 | 1.72E-13 | 7.13E-12 | - |
| Unigene71354_S2A | 61.28 | 25.87 | -1.24 | 8.73E-20 | 5.73E-18 | - |
| Unigene4088_S2A | 13.27 | 5.61 | -1.24 | 2.55E-05 | 3.37E-04 | - |
| Unigene73219_S2A | 113.83 | 48.13 | -1.24 | 2.62E-35 | 2.77E-33 | - |
| Unigene11719_S2A | 15.36 | 6.51 | -1.24 | 6.11E-06 | 9.05E-05 | - |
| Unigene44755_S2A | 19.20 | 8.14 | -1.24 | 4.15E-07 | 7.80E-06 | - |
| Unigene14671_S2A | 30.73 | 13.03 | -1.24 | 1.42E-10 | 4.23E-09 | - |
| Unigene12125_S2A | 23.04 | 9.77 | -1.24 | 2.87E-08 | 6.38E-07 | - |
| Unigene25580_S2A | 210.20 | 89.38 | -1.23 | 4.16E-63 | 6.71E-61 | CD2 antigen |
| Unigene26456_S2A | 37.01 | 15.74 | -1.23 | 2.19E-12 | 8.04E-11 | - |
| Unigene16672_S2A | 29.33 | 12.48 | -1.23 | 4.31E-10 | 1.19E-08 | - |
| Unigene26181_S2A | 37.36 | 15.92 | -1.23 | 1.90E-12 | 7.02E-11 | Protein ITFG3 |
| Unigene25384_S2A | 156.60 | 66.76 | -1.23 | 3.04E-47 | 4.17E-45 | Ferritin L subunit |
| Unigene2159_S2A | 17.81 | 7.60 | -1.23 | 1.27E-06 | 2.18E-05 | - |
| Unigene9597_S2A | 17.81 | 7.60 | -1.23 | 1.27E-06 | 2.18E-05 | - |
| CL5392.Contig2_S2A | 100.39 | 42.88 | -1.23 | 8.85E-31 | 8.67E-29 | PAOX protein |
| Unigene10591_S2A | 71.58 | 30.58 | -1.23 | 2.11E-22 | 1.59E-20 | - |
| Unigene45340_S2A | 13.97 | 5.97 | -1.23 | 1.89E-05 | 2.59E-04 | - |
| Unigene26073_S2A | 170.22 | 73.09 | -1.22 | 1.53E-50 | 2.16E-48 | Cholesteryl ester hydrolase precursor |
| Unigene11576_S2A | 14.32 | 6.15 | -1.22 | 1.63E-05 | 2.25E-04 | Zinc finger protein 358 |
| Unigene35767_S2A | 28.63 | 12.30 | -1.22 | 9.76E-10 | 2.61E-08 | - |
| CL592.Contig1_S2A | 59.36 | 25.51 | -1.22 | 1.21E-18 | 7.48E-17 | Putative p150 |
| Unigene72123_S2A | 18.51 | 7.96 | -1.22 | 9.49E-07 | 1.67E-05 | Integrin alpha 2b |
| Unigene4773_S2A | 16.41 | 7.06 | -1.22 | 3.92E-06 | 6.08E-05 | - |
| CL1989.Contig1_S2A | 24.79 | 10.67 | -1.22 | 1.39E-08 | 3.25E-07 | Protein LSM14 homolog A |
| Unigene51672_S2A | 65.12 | 28.04 | -1.22 | 3.10E-20 | 2.08E-18 | - |
| Unigene17385_S2A | 13.44 | 5.79 | -1.21 | 3.10E-05 | 4.02E-04 | - |
| CL1585.Contig1_S2A | 13.44 | 5.79 | -1.21 | 3.10E-05 | 4.01E-04 | Interferon-induced very large GTPase 1-like |
| CL232.Contig2_S2A | 23.92 | 10.31 | -1.21 | 2.60E-08 | 5.81E-07 | Tetraspanin-3-like isoform 1 |
| CL1345.Contig1_S2A | 14.66 | 6.33 | -1.21 | 1.40E-05 | 1.96E-04 | Lens-specific calpain Lp82 |
| Unigene25365_S2A | 59.88 | 25.87 | -1.21 | 1.26E-18 | 7.79E-17 | Tropomyosin alpha-3 chain |
| Unigene35952_S2A | 38.06 | 16.46 | -1.21 | 2.40E-12 | 8.66E-11 | CRE-PQN-75 protein |
| Unigene26879_S2A | 174.76 | 75.63 | -1.21 | 4.02E-51 | 5.78E-49 | CD82 antigen |
| CL241.Contig1_S2A | 21.30 | 9.23 | -1.21 | 1.72E-07 | 3.43E-06 | Collagen alpha-2(I) chain precursor |
| Unigene51487_S2A | 30.90 | 13.39 | -1.21 | 2.92E-10 | 8.30E-09 | - |
| Unigene5078_S2A | 45.92 | 19.90 | -1.21 | 1.50E-14 | 7.03E-13 | E3 ubiquitin-protein ligase RNF13 |
| Unigene58874_S2A | 17.11 | 7.42 | -1.21 | 2.91E-06 | 4.65E-05 | - |
| Unigene72058_S2A | 15.01 | 6.51 | -1.21 | 1.21E-05 | 1.71E-04 | B-cell CLL/lymphoma 9-like protein |
| Unigene8845_S2A | 12.92 | 5.61 | -1.20 | 5.06E-05 | 6.29E-04 | - |
| Unigene59705_S2A | 14.14 | 6.15 | -1.20 | 2.29E-05 | 3.07E-04 | - |
| CL2661.Contig3_S2A | 40.33 | 17.55 | -1.20 | 7.20E-13 | 2.81E-11 | Tyrosine-protein kinase HCK isoform 1 |
| Unigene21626_S2A | 13.27 | 5.79 | -1.20 | 4.35E-05 | 5.47E-04 | Uncharacterized protein C11orf84 homolo |
| Unigene25363_S2A | 141.24 | 61.70 | -1.19 | 6.08E-41 | 7.20E-39 | Actin-related protein 2/3 complex subunit 5 |
| Unigene73021_S2A | 53.42 | 23.34 | -1.19 | 1.84E-16 | 9.98E-15 | - |
| Unigene3827_S2A | 22.35 | 9.77 | -1.19 | 1.10E-07 | 2.25E-06 | - |
| Unigene16682_S2A | 16.93 | 7.42 | -1.19 | 4.07E-06 | 6.30E-05 | - |
| CL2745.Contig2_S2A | 13.62 | 5.97 | -1.19 | 3.74E-05 | 4.78E-04 | - |
| Unigene16098_S2A | 96.02 | 42.16 | -1.19 | 4.17E-28 | 3.76E-26 | - |
| Unigene11604_S2A | 66.34 | 29.13 | -1.19 | 6.72E-20 | 4.45E-18 | - |
| Unigene77148_S2A | 16.06 | 7.06 | -1.19 | 7.69E-06 | 1.12E-04 | POZ domain-containing protein 10 |
| CL509.Contig2_S2A | 17.28 | 7.60 | -1.19 | 3.50E-06 | 5.49E-05 | Histone H2A.J |
| Unigene43547_S2A | 12.74 | 5.61 | -1.18 | 7.10E-05 | 8.47E-04 | - |
| Unigene25532_S2A | 147.70 | 65.13 | -1.18 | 5.41E-42 | 6.61E-40 | Tartrate-resistant acid phosphatase type 5 precursor |
| Unigene10756_S2A | 56.56 | 24.97 | -1.18 | 4.96E-17 | 2.77E-15 | Poly [ADP-ribose] polymerase 14 |
| Unigene66558_S2A | 72.45 | 32.02 | -1.18 | 2.47E-21 | 1.76E-19 | - |
| Unigene11541_S2A | 35.61 | 15.74 | -1.18 | 3.10E-11 | 9.90E-10 | - |
| Unigene56980_S2A | 17.98 | 7.96 | -1.18 | 2.59E-06 | 4.17E-05 | - |
| Unigene35939_S2A | 16.76 | 7.42 | -1.18 | 5.68E-06 | 8.50E-05 | DIS3-like exonuclease 1 |
| Unigene27008_S2A | 174.23 | 77.25 | -1.17 | 9.94E-49 | 1.38E-46 | Transmembrane 9 superfamily member 2 |
| CL4103.Contig1_S2A | 60.75 | 26.96 | -1.17 | 5.18E-18 | 3.10E-16 | Adaptor-related protein complex 1, beta 1 subunit |
| Unigene66015_S2A | 28.11 | 12.48 | -1.17 | 4.40E-09 | 1.10E-07 | - |
| Unigene66807_S2A | 18.33 | 8.14 | -1.17 | 2.23E-06 | 3.63E-05 | - |
| Unigene11555_S2A | 47.66 | 21.17 | -1.17 | 2.00E-14 | 9.24E-13 | Transmembrane protein 189 |
| Unigene25974_S2A | 2177.22 | 969.94 | -1.17 | 0 | 0 | Envelope glycoprotein |
| Unigene78609_S2A | 13.79 | 6.15 | -1.16 | 4.48E-05 | 5.63E-04 | - |
| Unigene4289_S2A | 19.03 | 8.50 | -1.16 | 1.64E-06 | 2.74E-05 | - |
| Unigene59351_S2A | 19.03 | 8.50 | -1.16 | 1.64E-06 | 2.74E-05 | - |
| Unigene11876_S2A | 39.28 | 17.55 | -1.16 | 5.10E-12 | 1.77E-10 | - |
| Unigene9670_S2A | 27.06 | 12.12 | -1.16 | 1.14E-08 | 2.69E-07 | - |
| Unigene11348_S2A | 42.42 | 19.00 | -1.16 | 8.16E-13 | 3.14E-11 | - |
| Unigene25425_S2A | 103.00 | 46.14 | -1.16 | 6.09E-29 | 5.60E-27 | Rho GTPase activating protein 30 isoform 1 |
| Unigene25522_S2A | 25.84 | 11.58 | -1.16 | 2.47E-08 | 5.53E-07 | Tyrosine-protein kinase receptor UFO-like isoform 2 |
| Unigene35954_S2A | 12.92 | 5.79 | -1.16 | 8.50E-05 | 9.93E-04 | Polycomb protein EED-like |
| Unigene71362_S2A | 73.32 | 32.93 | -1.15 | 5.88E-21 | 4.12E-19 | - |
| Unigene26027_S2A | 15.71 | 7.06 | -1.15 | 1.49E-05 | 2.07E-04 | Ubiquitin-like protein 4A |
| Unigene81390_S2A | 13.27 | 5.97 | -1.15 | 7.28E-05 | 8.64E-04 | - |
| Unigene60626_S2A | 26.54 | 11.94 | -1.15 | 1.83E-08 | 4.21E-07 | Fibrosin-1-like protein |
| Unigene65639_S2A | 26.54 | 11.94 | -1.15 | 1.83E-08 | 4.20E-07 | - |
| Unigene14435_S2A | 29.68 | 13.39 | -1.15 | 2.88E-09 | 7.32E-08 | - |
| Unigene60343_S2A | 21.65 | 9.77 | -1.15 | 4.10E-07 | 7.71E-06 | - |
| Unigene71521_S2A | 39.28 | 17.73 | -1.15 | 8.34E-12 | 2.85E-10 | - |
| Unigene72613_S2A | 17.63 | 7.96 | -1.15 | 4.99E-06 | 7.56E-05 | - |
| Unigene72038_S2A | 17.63 | 7.96 | -1.15 | 4.99E-06 | 7.56E-05 | - |
| Unigene11405_S2A | 20.43 | 9.23 | -1.15 | 8.94E-07 | 1.59E-05 | - |
| Unigene25745_S2A | 60.06 | 27.14 | -1.15 | 3.05E-17 | 1.75E-15 | Echinoderm microtubule-associated protein-like 3 |
| Unigene35690_S2A | 13.97 | 6.33 | -1.14 | 5.33E-05 | 6.58E-04 | Coiled-coil domain-containing protein 86 |
| Unigene65464_S2A | 22.35 | 10.13 | -1.14 | 3.02E-07 | 5.83E-06 | - |
| Unigene73337_S2A | 25.14 | 11.40 | -1.14 | 5.48E-08 | 1.17E-06 | - |
| Unigene11243_S2A | 72.98 | 33.11 | -1.14 | 1.80E-20 | 1.22E-18 | AIP protein |
| Unigene77753_S2A | 33.87 | 15.38 | -1.14 | 2.89E-10 | 8.25E-09 | - |
| Unigene25790_S2A | 28.28 | 12.85 | -1.14 | 8.58E-09 | 2.08E-07 | Ras and Rab interactor 3 |
| Unigene71965_S2A | 99.86 | 45.41 | -1.14 | 2.59E-27 | 2.26E-25 | - |
| CL3335.Contig1_S2A | 2716.33 | 1237.35 | -1.13 | 0 | 0 | 60S ribosomal protein L18a-like |
| Unigene35851_S2A | 24.62 | 11.22 | -1.13 | 8.78E-08 | 1.83E-06 | Pre-mRNA-processing factor 39 |
| Unigene65714_S2A | 37.71 | 17.19 | -1.13 | 3.40E-11 | 1.08E-09 | Uncharacterized methyltransferase WBSCR22 |
| CL14.Contig1_S2A | 101.61 | 46.32 | -1.13 | 1.24E-27 | 1.10E-25 | Prostaglandin F synthase 2 |
| Unigene11915_S2A | 26.19 | 11.94 | -1.13 | 3.47E-08 | 7.60E-07 | - |
| Unigene60152_S2A | 23.39 | 10.67 | -1.13 | 1.91E-07 | 3.77E-06 | - |
| Unigene26638_S2A | 61.45 | 28.04 | -1.13 | 2.72E-17 | 1.57E-15 | - |
| Unigene16458_S2A | 20.60 | 9.41 | -1.13 | 1.06E-06 | 1.85E-05 | - |
| Unigene26952_S2A | 162.36 | 74.18 | -1.13 | 5.62E-43 | 6.98E-41 | Synaptogyrin-2 |
| Unigene65664_S2A | 17.81 | 8.14 | -1.13 | 5.92E-06 | 8.80E-05 | Zinc finger protein 33B |
| Unigene73607_S2A | 81.53 | 37.27 | -1.13 | 2.29E-22 | 1.72E-20 | - |
| Unigene71631_S2A | 76.64 | 35.10 | -1.13 | 4.79E-21 | 3.39E-19 | Influenza virus NS1A-binding protein |
| Unigene36189_S2A | 54.12 | 24.79 | -1.13 | 2.64E-15 | 1.32E-13 | - |
| Unigene25431_S2A | 51.68 | 23.70 | -1.12 | 1.22E-14 | 5.76E-13 | Nuclear mitotic apparatus protein 1 |
| Unigene26783_S2A | 44.17 | 20.26 | -1.12 | 1.02E-12 | 3.87E-11 | - |
| Unigene66845_S2A | 13.79 | 6.33 | -1.12 | 7.38E-05 | 8.75E-04 | - |
| Unigene26070_S2A | 823.68 | 378.31 | -1.12 | 2.76E-208 | 6.89E-206 | 40S ribosomal protein S5-like |
| Unigene25685_S2A | 52.37 | 24.06 | -1.12 | 9.02E-15 | 4.33E-13 | UPF0451 protein C17orf61 homolog isoform 2 |
| Unigene11917_S2A | 26.71 | 12.30 | -1.12 | 3.51E-08 | 7.68E-07 | - |
| CL4735.Contig1_S2A | 61.28 | 28.22 | -1.12 | 5.98E-17 | 3.31E-15 | Unnamed protein product |
| Unigene70502_S2A | 42.42 | 19.54 | -1.12 | 3.49E-12 | 1.24E-10 | Lysyl oxidase-like 1 |
| Unigene6355_S2A | 28.63 | 13.21 | -1.12 | 1.19E-08 | 2.82E-07 | - |
| Unigene27085_S2A | 16.06 | 7.42 | -1.11 | 2.09E-05 | 2.82E-04 | H(+) exchange regulatory cofactor NHE-RF1 |
| Unigene78281_S2A | 34.04 | 15.74 | -1.11 | 5.51E-10 | 1.50E-08 | - |
| Unigene35132_S2A | 30.90 | 14.29 | -1.11 | 3.49E-09 | 8.81E-08 | - |
| Unigene66246_S2A | 68.44 | 31.66 | -1.11 | 1.36E-18 | 8.34E-17 | - |
| Unigene17230_S2A | 27.76 | 12.85 | -1.11 | 2.22E-08 | 5.01E-07 | Synaptosomal-associated protein 23 |
| CL1520.Contig1_S2A | 16.41 | 7.60 | -1.11 | 1.79E-05 | 2.45E-04 | Family with sequence similarity 38, member A |
| Unigene66546_S2A | 67.56 | 31.30 | -1.11 | 2.50E-18 | 1.51E-16 | - |
| Unigene73291_S2A | 56.22 | 26.05 | -1.11 | 1.72E-15 | 8.72E-14 | - |
| CL365.Contig1_S2A | 46.44 | 21.53 | -1.11 | 4.84E-13 | 1.92E-11 | Apolipoprotein L, 3-like |
| Unigene4001_S2A | 46.44 | 21.53 | -1.11 | 4.84E-13 | 1.92E-11 | - |
| Unigene11574_S2A | 14.84 | 6.88 | -1.11 | 4.61E-05 | 5.78E-04 | Spermine synthase |
| Unigene50944_S2A | 18.33 | 8.50 | -1.11 | 5.97E-06 | 8.88E-05 | - |
| Unigene27023_S2A | 30.03 | 13.93 | -1.11 | 6.48E-09 | 1.59E-07 | - |
| CL2847.Contig2_S2A | 328.22 | 152.34 | -1.11 | 2.21E-82 | 4.13E-80 | - |
| Unigene71481_S2A | 33.52 | 15.56 | -1.11 | 8.77E-10 | 2.35E-08 | - |
| Unigene20542_S2A | 25.31 | 11.76 | -1.11 | 1.05E-07 | 2.14E-06 | Mediator of RNA polymerase II transcription subunit 30 |
| Unigene20485_S2A | 18.68 | 8.68 | -1.11 | 5.11E-06 | 7.72E-05 | - |
| CL2230.Contig2_S2A | 15.19 | 7.06 | -1.11 | 3.94E-05 | 5.02E-04 | Retinoic acid receptor alpha |
| Unigene25816_S2A | 1219.11 | 566.84 | -1.10 | 1.47E-299 | 4.09E-297 | Selenoprotein P precursor |
| Unigene72459_S2A | 20.60 | 9.59 | -1.10 | 1.71E-06 | 2.85E-05 | - |
| Unigene73950_S2A | 15.54 | 7.24 | -1.10 | 3.37E-05 | 4.35E-04 | Tetraspanin-11 |
| Unigene5045_S2A | 29.50 | 13.75 | -1.10 | 1.03E-08 | 2.45E-07 | CD1D antigen, d polypeptide-like |
| Unigene66316_S2A | 54.30 | 25.33 | -1.10 | 7.96E-15 | 3.84E-13 | - |
| Unigene15994_S2A | 26.36 | 12.30 | -1.10 | 6.58E-08 | 1.39E-06 | - |
| Unigene11178_S2A | 29.85 | 13.93 | -1.10 | 8.85E-09 | 2.14E-07 | Fatty acid desaturase 6 |
| Unigene25505_S2A | 45.74 | 21.35 | -1.10 | 1.05E-12 | 3.96E-11 | Inc finger CCCH domain-containing protein 11A |
| Unigene35294_S2A | 19.38 | 9.05 | -1.10 | 3.74E-06 | 5.82E-05 | - |
| Unigene27011_S2A | 1774.46 | 829.36 | -1.10 | 0 | 0 | Heme oxygenase 1 |
| Unigene5104_S2A | 30.55 | 14.29 | -1.10 | 6.51E-09 | 1.59E-07 | - |
| Unigene375_S2A | 35.96 | 16.83 | -1.10 | 3.01E-10 | 8.56E-09 | - |
| Unigene66444_S2A | 36.31 | 17.01 | -1.09 | 2.58E-10 | 7.45E-09 | - |
| CL1708.Contig1_S2A | 49.41 | 23.16 | -1.09 | 1.68E-13 | 6.99E-12 | Band 4.1-like protein 3 isoform 1 |
| Unigene10441_S2A | 16.59 | 7.78 | -1.09 | 2.10E-05 | 2.83E-04 | - |
| Unigene60344_S2A | 26.54 | 12.48 | -1.09 | 7.70E-08 | 1.62E-06 | Zinc finger CCHC domain-containing protein 7 |
| Unigene25712_S2A | 147.70 | 69.48 | -1.09 | 5.09E-37 | 5.58E-35 | LIM domain-binding protein 1 |
| Unigene35203_S2A | 30.73 | 14.47 | -1.09 | 7.60E-09 | 1.86E-07 | - |
| Unigene71764_S2A | 117.15 | 55.18 | -1.09 | 1.28E-29 | 1.21E-27 | Hypothetical protein PANDA_004549 |
| Unigene11849_S2A | 71.40 | 33.65 | -1.09 | 1.19E-18 | 7.45E-17 | Actin-related protein 2 isoform |
| Unigene66653_S2A | 36.84 | 17.37 | -1.08 | 2.59E-10 | 7.45E-09 | - |
| Unigene35686_S2A | 109.29 | 51.56 | -1.08 | 1.19E-27 | 1.06E-25 | - |
| Unigene80285_S2A | 21.47 | 10.13 | -1.08 | 1.47E-06 | 2.47E-05 | - |
| Unigene65964_S2A | 17.63 | 8.32 | -1.08 | 1.31E-05 | 1.84E-04 | - |
| Unigene29485_S2A | 23.39 | 11.04 | -1.08 | 4.94E-07 | 9.20E-06 | - |
| Unigene65530_S2A | 25.66 | 12.12 | -1.08 | 1.43E-07 | 2.87E-06 | - |
| Unigene1544_S2A | 27.58 | 13.03 | -1.08 | 4.84E-08 | 1.04E-06 | - |
| Unigene25612_S2A | 321.41 | 151.98 | -1.08 | 1.21E-77 | 2.21E-75 | T-cell surface glycoprotein CD3 epsilon chain precursor |
| Unigene43797_S2A | 20.25 | 9.59 | -1.08 | 3.20E-06 | 5.05E-05 | - |
| CL2774.Contig2_S2A | 2921.99 | 1384.44 | -1.08 | 0 | 0 | Hypothetical protein TTHERM_02141640 |
| Unigene71822_S2A | 139.67 | 66.22 | -1.08 | 1.57E-34 | 1.62E-32 | - |
| Unigene75963_S2A | 20.60 | 9.77 | -1.08 | 2.74E-06 | 4.38E-05 | - |
| Unigene71718_S2A | 74.37 | 35.28 | -1.08 | 4.13E-19 | 2.65E-17 | - |
| Unigene4673_S2A | 18.68 | 8.87 | -1.07 | 8.17E-06 | 1.18E-04 | - |
| Unigene76426_S2A | 31.60 | 15.02 | -1.07 | 6.50E-09 | 1.59E-07 | - |
| Unigene26845_S2A | 143.86 | 68.39 | -1.07 | 2.56E-35 | 2.71E-33 | Heterogeneous nuclear ribonucleoprotein M, partial |
| Unigene81880_S2A | 19.03 | 9.05 | -1.07 | 6.98E-06 | 1.02E-04 | - |
| Unigene527_S2A | 53.25 | 25.33 | -1.07 | 4.96E-14 | 2.20E-12 | T-cell immunoglobulin and mucin domain-containing protein 4 precursor |
| Unigene71441_S2A | 94.27 | 44.87 | -1.07 | 1.19E-23 | 9.16E-22 | - |
| Unigene23122_S2A | 28.11 | 13.39 | -1.07 | 4.83E-08 | 1.04E-06 | - |
| Unigene70967_S2A | 43.30 | 20.63 | -1.07 | 1.21E-11 | 4.03E-10 | Leucine-rich repeat-containing protein 8D |
| Unigene71717_S2A | 69.48 | 33.11 | -1.07 | 8.54E-18 | 5.05E-16 | Para-hydroxybenzoate--polyprenyltransferase, mitochondrial precurso |
| Unigene26273_S2A | 41.73 | 19.90 | -1.07 | 3.01E-11 | 9.63E-10 | - |
| Unigene25788_S2A | 26.54 | 12.66 | -1.07 | 1.22E-07 | 2.47E-06 | Ras-related protein Rab-14 |
| Unigene66780_S2A | 28.81 | 13.75 | -1.07 | 3.54E-08 | 7.73E-07 | Chaperone, ABC1 activity of bc1 complex like |
| CL2847.Contig1_S2A | 53.07 | 25.33 | -1.07 | 6.71E-14 | 2.93E-12 | - |
| Unigene25835_S2A | 82.93 | 39.62 | -1.07 | 7.96E-21 | 5.48E-19 | NADH dehydrogenase |
| Unigene11735_S2A | 60.93 | 29.13 | -1.06 | 1.10E-15 | 5.66E-14 | Prolylcarboxypeptidase precursor |
| Unigene12021_S2A | 56.39 | 26.96 | -1.06 | 1.25E-14 | 5.90E-13 | HCG2033193 |
| Unigene70909_S2A | 38.58 | 18.45 | -1.06 | 1.89E-10 | 5.54E-09 | - |
| Unigene35764_S2A | 61.63 | 29.49 | -1.06 | 8.09E-16 | 4.21E-14 | - |
| Unigene66491_S2A | 27.23 | 13.03 | -1.06 | 8.93E-08 | 1.85E-06 | - |
| Unigene25241_S2A | 29.85 | 14.29 | -1.06 | 2.22E-08 | 5.00E-07 | Semaphorin-4A precursor |
| Unigene11061_S2A | 60.41 | 28.95 | -1.06 | 1.73E-15 | 8.73E-14 | - |
| Unigene26977_S2A | 96.20 | 46.14 | -1.06 | 1.01E-23 | 7.78E-22 | Transcription factor PU.1-like |
| Unigene73631_S2A | 16.59 | 7.96 | -1.06 | 3.34E-05 | 4.32E-04 | STAM-binding protein |
| Unigene65228_S2A | 47.14 | 22.62 | -1.06 | 2.23E-12 | 8.14E-11 | - |
| Unigene59554_S2A | 17.28 | 8.32 | -1.05 | 2.43E-05 | 3.23E-04 | - |
| Unigene26057_S2A | 32.30 | 15.56 | -1.05 | 7.49E-09 | 1.83E-07 | - |
| Unigene25907_S2A | 293.65 | 141.48 | -1.05 | 2.73E-68 | 4.64E-66 | Tristetraprolin |
| Unigene75976_S2A | 132.16 | 63.69 | -1.05 | 1.14E-31 | 1.14E-29 | - |
| Unigene71809_S2A | 17.63 | 8.50 | -1.05 | 2.08E-05 | 2.81E-04 | - |
| Unigene48705_S2A | 22.87 | 11.04 | -1.05 | 1.24E-06 | 2.13E-05 | - |
| Unigene21251_S2A | 22.87 | 11.04 | -1.05 | 1.24E-06 | 2.13E-05 | Zinc finger with UFM1-specific peptidase domain protein ZUP1 |
| Unigene25635_S2A | 17.98 | 8.68 | -1.05 | 1.77E-05 | 2.43E-04 | - |
| Unigene66289_S2A | 28.81 | 13.93 | -1.05 | 5.54E-08 | 1.18E-06 | - |
| Unigene71795_S2A | 23.57 | 11.40 | -1.05 | 9.05E-07 | 1.61E-05 | - |
| Unigene4986_S2A | 15.71 | 7.60 | -1.05 | 6.25E-05 | 7.59E-04 | Heat shock-related 70 kDa protein 2-like |
| Unigene60439_S2A | 56.74 | 27.50 | -1.04 | 2.61E-14 | 1.19E-12 | - |
| Unigene5026_S2A | 16.41 | 7.96 | -1.04 | 4.54E-05 | 5.70E-04 | - |
| Unigene12092_S2A | 41.38 | 20.08 | -1.04 | 8.57E-11 | 2.61E-09 | - |
| Unigene71996_S2A | 77.51 | 37.63 | -1.04 | 6.16E-19 | 3.90E-17 | Inc finger CCCH domain-containing protein 7A |
| Unigene26021_S2A | 38.76 | 18.82 | -1.04 | 3.41E-10 | 9.58E-09 | - |
| Unigene71831_S2A | 103.18 | 50.12 | -1.04 | 1.15E-24 | 9.13E-23 | Probable E3 ubiquitin-protein ligase HERC3 |
| Unigene65548_S2A | 37.19 | 18.09 | -1.04 | 8.53E-10 | 2.29E-08 | - |
| Unigene4267_S2A | 20.08 | 9.77 | -1.04 | 6.84E-06 | 1.01E-04 | - |
| Unigene10931_S2A | 17.46 | 8.50 | -1.04 | 2.81E-05 | 3.69E-04 | - |
| Unigene71256_S2A | 17.46 | 8.50 | -1.04 | 2.81E-05 | 3.69E-04 | - |
| Unigene10855_S2A | 23.04 | 11.22 | -1.04 | 1.43E-06 | 2.42E-05 | Surfeit 1-like |
| Unigene5202_S2A | 20.78 | 10.13 | -1.04 | 4.99E-06 | 7.56E-05 | Protein GAPT |
| Unigene37713_S2A | 20.78 | 10.13 | -1.04 | 4.99E-06 | 7.55E-05 | - |
| Unigene16036_S2A | 23.74 | 11.58 | -1.04 | 1.05E-06 | 1.83E-05 | - |
| Unigene51024_S2A | 30.03 | 14.65 | -1.04 | 4.00E-08 | 8.69E-07 | - |
| Unigene20615_S2A | 21.12 | 10.31 | -1.03 | 4.26E-06 | 6.55E-05 | - |
| Unigene11205_S2A | 21.12 | 10.31 | -1.03 | 4.26E-06 | 6.54E-05 | - |
| Unigene65836_S2A | 18.16 | 8.87 | -1.03 | 2.05E-05 | 2.78E-04 | - |
| Unigene25483_S2A | 70.36 | 34.38 | -1.03 | 4.22E-17 | 2.37E-15 | Chromosome 6 open reading frame 86-like |
| Unigene11242_S2A | 52.20 | 25.51 | -1.03 | 4.61E-13 | 1.83E-11 | - |
| CL3941.Contig1_S2A | 30.73 | 15.02 | -1.03 | 2.93E-08 | 6.51E-07 | - |
| Unigene26461_S2A | 77.34 | 37.81 | -1.03 | 1.27E-18 | 7.86E-17 | Glutathione S-transferase P |
| Unigene58466_S2A | 18.51 | 9.05 | -1.03 | 1.75E-05 | 2.40E-04 | - |
| Unigene25226_S2A | 1218.94 | 596.15 | -1.03 | 3.28E-268 | 8.80E-266 | 40S ribosomal protein S7-like |
| CL5129.Contig3_S2A | 34.39 | 16.83 | -1.03 | 4.58E-09 | 1.14E-07 | Reverse transcriptase-like |
| Unigene70537_S2A | 28.46 | 13.93 | -1.03 | 1.01E-07 | 2.06E-06 | - |
| Unigene35440_S2A | 31.42 | 15.38 | -1.03 | 2.14E-08 | 4.88E-07 | - |
| Unigene35382_S2A | 31.42 | 15.38 | -1.03 | 2.14E-08 | 4.88E-07 | - |
| Unigene36992_S2A | 18.85 | 9.23 | -1.03 | 1.49E-05 | 2.07E-04 | - |
| Unigene66215_S2A | 19.20 | 9.41 | -1.03 | 1.27E-05 | 1.79E-04 | - |
| CL3519.Contig2_S2A | 416.73 | 204.26 | -1.03 | 1.56E-92 | 2.99E-90 | Galectin 1 |
| Unigene70451_S2A | 22.52 | 11.04 | -1.03 | 2.26E-06 | 3.68E-05 | - |
| Unigene11035_S2A | 43.12 | 21.17 | -1.03 | 6.10E-11 | 1.88E-09 | NEDD8-conjugating enzyme UBE2F |
| Unigene11542_S2A | 110.16 | 54.10 | -1.03 | 1.28E-25 | 1.07E-23 | - |
| Unigene26441_S2A | 37.19 | 18.27 | -1.03 | 1.32E-09 | 3.47E-08 | 14-3-3 protein beta |
| Unigene7917_S2A | 41.20 | 20.26 | -1.02 | 1.78E-10 | 5.24E-09 | - |
| Unigene71428_S2A | 20.60 | 10.13 | -1.02 | 6.73E-06 | 9.90E-05 | - |
| Unigene59320_S2A | 20.60 | 10.13 | -1.02 | 6.73E-06 | 9.89E-05 | Circumsporozoite protei |
| Unigene66448_S2A | 34.22 | 16.83 | -1.02 | 6.15E-09 | 1.51E-07 | Dyslexia susceptibility 2-like |
| Unigene73795_S2A | 24.27 | 11.94 | -1.02 | 1.03E-06 | 1.80E-05 | 60S ribosomal protein L34-like |
| CL96.Contig1_S2A | 280.90 | 138.41 | -1.02 | 3.06E-62 | 4.86E-60 | RNA binding motif protein, X-linked 2-like protein |
| Unigene26460_S2A | 166.55 | 82.14 | -1.02 | 1.56E-37 | 1.73E-35 | Peroxiredoxin-1 |
| Unigene70558_S2A | 25.66 | 12.66 | -1.02 | 5.47E-07 | 1.01E-05 | - |
| Unigene59127_S2A | 40.33 | 19.90 | -1.02 | 3.25E-10 | 9.19E-09 | - |
| Unigene35312_S2A | 219.97 | 108.56 | -1.02 | 5.93E-49 | 8.30E-47 | - |
| Unigene65243_S2A | 29.68 | 14.65 | -1.02 | 7.23E-08 | 1.52E-06 | - |
| Unigene66764_S2A | 23.39 | 11.58 | -1.01 | 1.89E-06 | 3.13E-05 | - |
| Unigene10416_S2A | 24.09 | 11.94 | -1.01 | 1.38E-06 | 2.35E-05 | - |
| Unigene4032_S2A | 36.49 | 18.09 | -1.01 | 2.76E-09 | 7.06E-08 | - |
| Unigene11675_S2A | 20.43 | 10.13 | -1.01 | 9.05E-06 | 1.30E-04 | - |
| Unigene4899_S2A | 33.17 | 16.46 | -1.01 | 1.51E-08 | 3.53E-07 | Hypothetical protein LOC100427008 |
| Unigene27107_S2A | 816.52 | 405.27 | -1.01 | 2.43E-174 | 5.81E-172 | - |
| Unigene25634_S2A | 272.87 | 135.51 | -1.01 | 1.92E-59 | 3.02E-57 | Cystatin-B |
| Unigene25830_S2A | 323.50 | 160.66 | -1.01 | 3.94E-70 | 6.80E-68 | Myosin-IXb |
| Unigene71065_S2A | 17.11 | 8.50 | -1.01 | 5.13E-05 | 6.35E-04 | - |
| Unigene11268_S2A | 42.60 | 21.17 | -1.01 | 1.46E-10 | 4.35E-09 | OGDH protein |
| Unigene36130_S2A | 34.22 | 17.01 | -1.01 | 9.44E-09 | 2.27E-07 | - |
| Unigene26747_S2A | 91.31 | 45.41 | -1.01 | 6.40E-21 | 4.45E-19 | Calmodulin |
| CL141.Contig3_S2A | 30.20 | 15.02 | -1.01 | 7.08E-08 | 1.49E-06 | P97Bcnt protein |
| Unigene35614_S2A | 57.09 | 28.41 | -1.01 | 1.24E-13 | 5.29E-12 | - |
| Unigene66393_S2A | 35.96 | 17.91 | -1.01 | 4.32E-09 | 1.08E-07 | - |
| Unigene59425_S2A | 22.52 | 11.22 | -1.01 | 3.49E-06 | 5.47E-05 | - |
| CL90.Contig3_S2A | 579.44 | 288.94 | -1.00 | 5.82E-123 | 1.27E-120 | Actin, aortic smooth muscle-like isoform 1 |
| Unigene26168_S2A | 18.85 | 9.41 | -1.00 | 2.30E-05 | 3.08E-04 | Heterogeneous nuclear ribonucleoprotein G |
| Unigene17119_S2A | 28.98 | 14.47 | -1.00 | 1.52E-07 | 3.04E-06 | Carboxy-terminal domain RNA polymerase II polypeptide A small phosphatase 1-like, partial |
| Unigene245_S2A | 123.78 | 61.88 | -1.00 | 1.75E-27 | 1.54E-25 | - |
| Unigene80457_S2A | 19.90 | 9.95 | -1.00 | 1.43E-05 | 1.99E-04 | - |

Note*: “-” indicates that there is no gene name.
